# Cooperative NHC and Photoredox Catalysis for the Synthesis of β‐Trifluoromethylated Alkyl Aryl Ketones

**DOI:** 10.1002/anie.202008040

**Published:** 2020-09-01

**Authors:** Qing‐Yuan Meng, Nadine Döben, Armido Studer

**Affiliations:** ^1^ Organisch-Chemisches Institut Westfälische Wilhelms-Universität Corrensstrasse 40 48149 Münster Germany

**Keywords:** NHC catalysis, trifluoromethylation, visible light catalysis

## Abstract

Despite the great potential of radical chemistry in organic synthesis, N‐heterocyclic carbene (NHC)‐catalyzed reactions involving radical intermediates are not well explored. This communication reports the three‐component coupling of aroyl fluorides, styrenes and the Langlois reagent (CF_3_SO_2_Na) to give various β‐trifluoromethylated alkyl aryl ketones with good functional group tolerance in moderate to high yields by cooperative photoredox/NHC catalysis. The alkene acyltrifluoromethylation proceeds via radical/radical cross coupling of ketyl radicals with benzylic C‐radicals. The ketyl radicals are generated via SET reduction of in situ formed acylazolium ions whereas the benzylic radicals derive from trifluoromethyl radical addition onto styrenes.

## Graphical abstract

Photoredox and NHC catalysis in concert: A novel strategy for the preparation of β‐trifluoromethylketones via radical alkene acyltrifluoromethylation has been developed. The three‐component cascades proceed under mild conditions with readily available aroyl fluorides, styrenes and the Langlois reagent as the reaction partners.

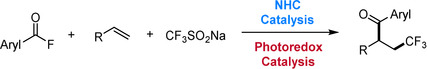

Due to their unique Lewis basicity and nucleophilicity, N‐heterocyclic carbenes (NHCs) have been widely used as organocatalysts in organic synthesis.^[1]^ Generally, NHC‐catalyzed reactions proceed via the formation of Breslow,^[2]^ π‐extended Breslow^[3]^ or azolium intermediates^[4]^ that react with electrophiles or nucleophiles in ionic pathways. In contrast, NHC‐catalysis that proceeds via single electron transfer (SET) processes involving the coupling of radical intermediates is far less explored.

In 2008, we developed an NHC‐catalyzed oxidation of aldehydes into TEMPO‐esters (TEMPO=2,2,6,6‐tetramethylpiperidine‐N‐oxyl) and showed that Breslow intermediates are readily SET‐oxidized by the TEMPO radical to the corresponding radical cations.^[5]^ Considering SET‐oxidation of π‐extended Breslow intermediates, the radical cations derived therefrom were found to express radical reactivity at the β‐position that was successfully used for β‐C−O and β‐C−C bond formation via radical/radical cross coupling and homo coupling reactions (Scheme 1 a, path a).^[6]^ On the other hand, enals bearing γ‐leaving groups react with NHCs to dienol or trienol intermediates, that act as acceptors for electron‐deficient alkyl radicals at the γ‐ or ϵ‐position (Scheme 1 a, path b).^[7]^ Although Fukuzumi and co‐workers disclosed the redox behavior of Breslow intermediates by electrolysis as early as 1997,^[8]^ utilization of SET‐oxidized Breslow intermediates in radical cross coupling remained unexplored. Rehbein and co‐workers suggested that NHC‐catalyzed benzoin condensation may proceed via coupling of radical pairs generated via SET‐oxidation of Breslow intermediates.^[9]^ Very recently, Nagao and Ohmiya reported the trapping of such radical cations by tertiary alkyl radicals (Scheme 1 b).^[10]^ Following this protocol, different radical addition/cross coupling cascades were designed which allow the preparation of various β‐substituted alkyl aryl ketones.^[11]^ Conversely, reduction of an acylazolium intermediate via SET should provide a neutral persistent ketyl‐type radical that might express similar radical reactivity as its protonated congener generated by SET‐oxidation of the corresponding Breslow intermediate. However, application of this “reductive” strategy to realize the cross coupling between ketyl radicals and alkyl radicals is little explored (Scheme 1 c).^[12]^

**Scheme 1:**
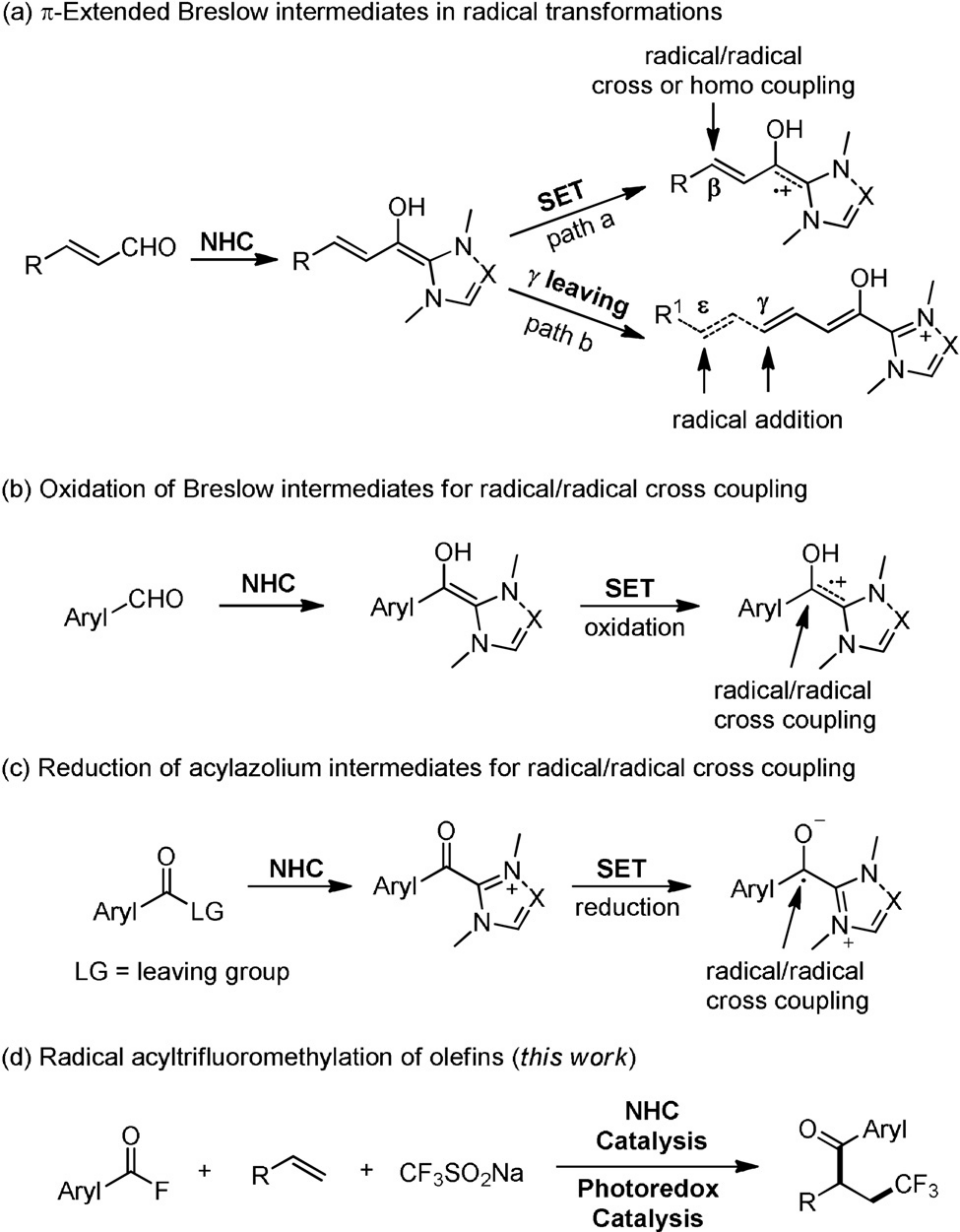
NHC catalyzed radical transformations.

Photoredox catalysis has matured to an important field in organic chemistry.^[13]^ In particular, the combination of photocatalysis with metal catalysis, organocatalysis, as well as enzymatic catalysis has opened new synthetic avenues. However, only few examples on cooperative photoredox/NHC catalysis have been disclosed to date.[^7^, ^14^, ^15^] In 2012, Rovis and DiRocco combined visible light redox catalysis with chiral NHC catalysis to realize a highly efficient asymmetric α‐acylation of *N*‐phenyltetrahydroisoquinolines with aliphatic aldehydes.^[14a]^ In this context and our continuous interests in NHC catalysis^[16]^ and photoredox catalysis,^[17]^ we sought to develop a redox‐neutral method for the radical acyltrifluoromethylation of alkenes with acyl fluorides and the Langlois reagent (CF_3_SO_2_Na) by cooperative photoredox/NHC catalysis, that should allow accessing β‐trifluoromethylated α‐substituted alkyl aryl ketones (Scheme 1 d).

We commenced our investigations with styrene (**1 a**) and benzoyl fluoride (**2 a**, 2 equiv) as the additional reaction components along with CF_3_SO_2_Na (1.3 equiv) to target ketone **3 aa** (Table 1). Cascades were run in dichloromethane in the presence of Cs_2_CO_3_ as the base (1.3 equiv). NHC (13 mol‐%) and redox catalyst (1.3 mol‐%) were varied and also the effect of the light source on the reaction outcome was addressed. Of note, ketones of type **3 aa** represent an important structural motif that can be found in drug candidates.^[18]^

**Table 1:** Optimization of the reaction conditions^[a]^ 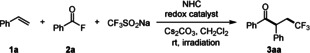

Entry	NHC Precursor	Photoredox Catalyst	Light Source	Yield^[b]^ [%]
1	**A**	4CzIPN	Blue LEDs	trace
2	**B**	4CzIPN	Blue LEDs	4
3	**C**	4CzIPN	Blue LEDs	2
4	**D**	4CzIPN	Blue LEDs	3
5	**E**	4CzIPN	Blue LEDs	15
6	**F**	4CzIPN	Blue LEDs	36
7	**G**	4CzIPN	Blue LEDs	7
8	**H**	4CzIPN	Blue LEDs	11
9	**F**	[Ir(dF(CF_3_)ppy)_2_(dtbbpy)]PF_6_	Blue LEDs	32
10	**F**	[Ir(ppy)_2_(dtbbpy)]PF_6_	Blue LEDs	39
11	**F**	[Ir(ppy)_2_(dtbbpy)]PF_6_	CFL	44
12	**F**	[Ir(ppy)_2_(dtbbpy)]PF_6_	CFL	69^[c]^
13	**F**	[Ir(ppy)_2_(dtbbpy)]PF_6_	CFL	80(74)^[d]^
14	**F**	[Ir(ppy)_2_(dtbbpy)]PF_6_	–	0
15	–	[Ir(ppy)_2_(dtbbpy)]PF_6_	CFL	0
16	**F**	–	CFL	0
				
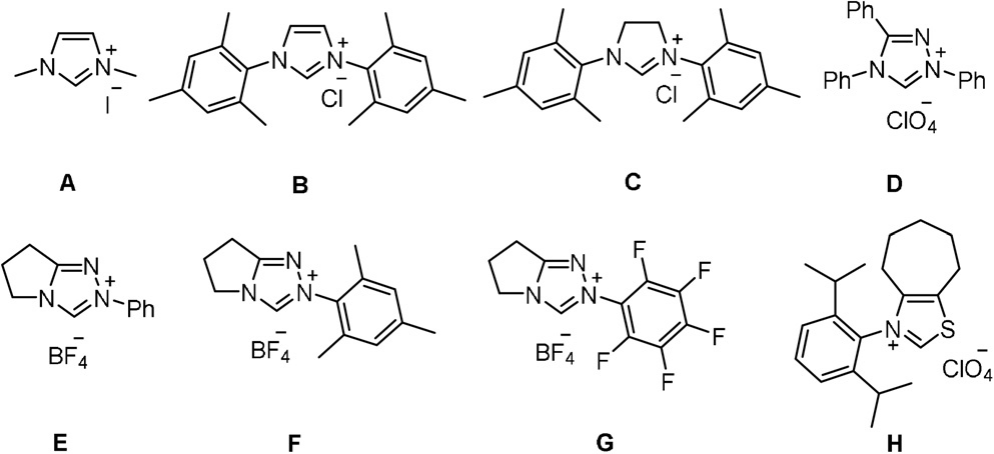				

[a] Unless otherwise noted, all the reactions were carried out with benzoyl fluoride (0.3 mmol), styrene (0.15 mmol), CF_3_SO_2_Na (0.2 mmol), NHC (0.02 mmol), base (0.2 mmol), and 4CzIPN (0.002 mmol) in anhydrous CH_2_Cl_2_ (2 mL), irradiation with blue LEDs at room temperature for 24 h. [b] GC‐FID yield using 1,3,5‐trimethoxybenzene as an internal standard, yield of isolated product is given in parentheses. [c] 0.1 mmol of styrene was used. [d] 0.4 mmol of benzoyl fluoride was used. NHC=*N*‐Heterocyclic carbenes. 4CzIPN=2,4,5,6‐tetra(carbazol‐9‐yl)isophthalonitrile. CFL=compact fluorescent lamp.

Initial experiments were conducted using 4CzIPN as the redox catalyst upon blue LED irradiation. Traces of the desired product **3 aa** were observed when the simplest imidazolium salt **A** was chosen as the precatalyst (Table 1, entry 1). NHC‐screening revealed that the triazolium salt **F** provides the best result and the targeted ketone **3 aa** was formed in 36 % yield (Table 1, entries 2–7). Solvent optimization showed that comparable yields can be obtained in acetonitrile and acetone, but other typical solvents gave inferior yields (Table S1, entries 1–7). Next, iridium based photocatalysts were used in place of 4CzIPN. [Ir(dF(CF_3_)ppy)_2_(dtbbpy)]PF_6_ provided a slightly lower yield but a measurable improvement was achieved with [Ir(ppy)_2_(dtbbpy)]PF_6_ (Table 1, entries 9 and 10). A further increase in yield was noted upon changing the light source (Table 1, entry 11). However, reaction did not go to completion. Therefore, the amount of **2 a** and Langlois reagent were increased to 3 and 2 equivalents, respectively. At the same time catalysts loadings were increased (20 mol‐% NHC and 2 mol‐% Ir‐cat) and a significantly improved yield was obtained (69 %, Table 1, entry 12). The best result was achieved upon using 4 equivalents of the fluoride providing **3 aa** in 80 % yield (Table 1, 13). Notably, with benzoyl chloride in place of benzoyl fluoride under otherwise identical conditions, targeted **3 aa** was formed in traces only. Control experiments revealed that the cascade does not occur in the absence of light, carbene or photoredox catalyst (Table 1, entries 14–16).

With optimized conditions in hand, the scope with respect to the acyl fluoride was explored, keeping styrene as the alkene component. We found that various aryl (β‐trifluoromethyl‐α‐phenyl)ethyl ketones **3 ab**–**3 aq** could be obtained in moderate to excellent yields (Scheme 2). Electronic effects in the aroyl fluoride are rather small and yields ranging from 67–88 % were obtained for systems bearing electron withdrawing or donating substituents at the *para*‐position (see **3 ab**–**3 ah**). As expected, *meta*‐substituents at the aryl group do not influence reaction outcome to a large extent (**3 ai**–**3 ak**). However, steric effects can be observed for *ortho*‐substituted aroyl fluorides (**3 al**, 73 %; **3 am**, 41 % and **3 an**, 46 %). Notably, a range of functional groups including fluoro, chloro, bromo, iodo, cyano and trifluoromethyl are tolerated, providing the basis for subsequent conversion of the corresponding products into more complex compounds. Particularly, the product bearing the medicinally relevant trifluoromethoxy group was formed in a good yield (**3 ag**, 74 %). Heteroaroyl fluorides containing the furan and thiophene moieties engage in the three‐component cascade, albeit lower yields were obtained for these two cases (**3 ap**, 46 % and **3 aq**, 40 %).

**Scheme 2:**
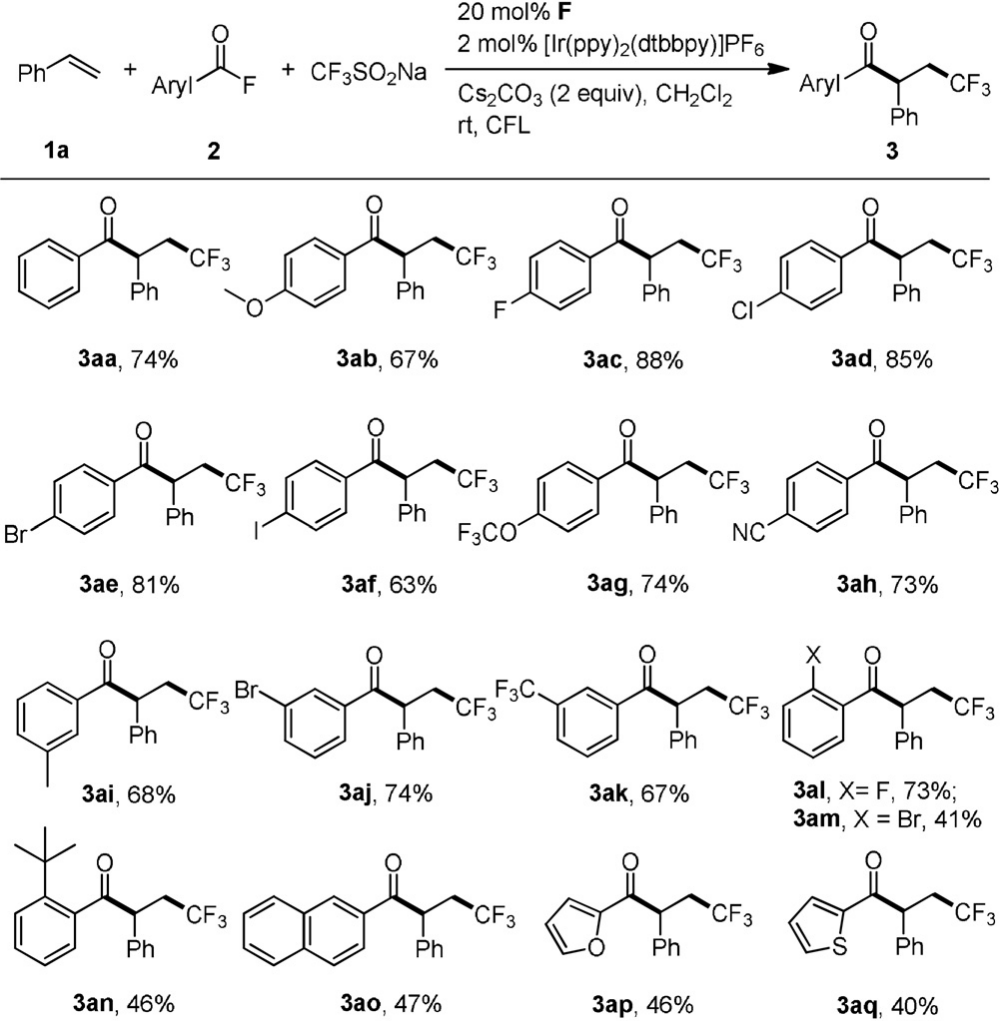
Reaction of styrene and CF_3_SO_2_Na with various acyl fluorides (Unless otherwise noted, all the reactions were carried out with acyl fluoride (0.4 mmol), styrene (0.1 mmol), CF_3_SO_2_Na (0.2 mmol), **F** (0.02 mmol), Cs_2_CO_3_ (0.2 mmol), and [Ir(ppy)_2_(dtbbpy)]PF_6_ (0.002 mmol) in CH_2_Cl_2_ (2 mL) under Ar, irradiation with CFL for 24 h. Isolated yields).

Next, we explored the reaction scope by varying the alkene component using benzoyl fluoride **2 a** as the coupling partner under the standard conditions (Scheme 3). Styrenes with methyl substituents in *ortho*‐, *meta*‐ and *para*‐position proceeded well, affording the corresponding products **3 ba**–**3 da** in 56–67 % yields. The radical acyltrifluoromethylation worked also well with 4‐methoxy‐ and 4‐phenylstyrene as the radical acceptors to provide the ketones **3 ea** and **3 ja** in 75 % and 73 % yields, respectively. Halogenated styrene derivatives were also tolerated to give the β‐trifluoromethylketones **3 fa**–**3 ia** in moderate to good yields (41–60 %). The lower yields generally observed for the electron‐poorer styrenes might be caused by the slower addition of the electrophilic CF_3_‐radical to such alkenes. 2‐Vinylnaphthalene and 2‐vinylpyridine engaged in the cascade to afford the corresponding products **3 ka** and **3 la** (52–53 %). However, nonactivated alkenes such as allylbenzene and 1‐octene did not work well, and the corresponding products **3 ma** and **3 na** were isolated in 15 % and 20 % yields, respectively. Conversion of the starting materials was low for these examples. Hence, the cascade is obviously very sensitive towards the rate constant for the CF_3_‐radical addition to the alkene component (see suggested mechanism below).^[19]^ Indene reacted with excellent diastereoselectivity to give *trans*‐**3 oa** as a single isomer in 55 % yield. However, reaction of α‐methylstyrene, *trans*‐β‐methylstyrene and *cis*‐β‐methylstyrene did not work. Finally, dearomative difunctionalization of benzofuran was achieved and the targeted product **3 pa** was obtained in 36 % yield with complete regioselectivity and excellent diastereoselectivity.

**Scheme 3:**
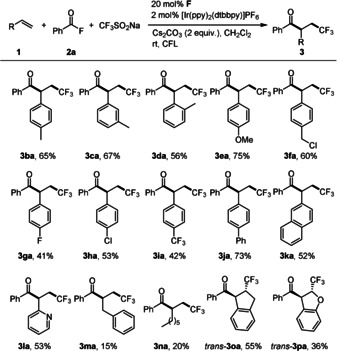
Reaction of benzoyl fluoride and CF_3_SO_2_Na with various alkenes (Unless otherwise noted, all the reactions were carried out with benzoyl fluoride (0.4 mmol), olefins (0.1 mmol), CF_3_SO_2_Na (0.2 mmol), **F** (0.02 mmol), Cs_2_CO_3_ (0.2 mmol), and [Ir(ppy)_2_(dtbbpy)]PF_6_ (0.002 mmol) in CH_2_Cl_2_ (2 mL) under Ar, irradiation with CFL for 24 h. Isolated yields).

Mechanistic studies were conducted next (Scheme 4). Using the acylazolium ion **5** as the substrate in combination with the Langlois reagent and styrene under Ir‐photoredox catalysis provided ketone **3 aa** in 83 % yield. This result strongly indicates that acyl azoliums, derived from acyl fluorides and **F** are competent intermediates in these cascades (Scheme 4, equation 1). Moreover, the reaction is suppressed in the presence of TEMPO supporting the radical nature of the transformation (Scheme 4, equation 2), which was further documented by the acyltrifluoromethylation of (2‐vinylcyclopropyl)benzene **6** to give exclusively the ring opening product **7** in 55 % yield (Scheme 4, equation 3). Based on these results and the fact that both catalysts and light are required (see Table 1), a possible mechanism involving cooperative NHC and photoredox catalysis for the radical alkene acyltrifluoromethylation is proposed in Scheme 5. Upon visible light irradiation, the excited state of [Ir(ppy)_2_(dtbbpy)]PF_6_[^13b^, ^20^] undergoes known reductive quenching by the trifluoromethanesulfinate anion^[21]^ to give the corresponding Ir^II^‐complex [*E*_1/2_(Ir^III^/Ir^II^)=−1.51 V vs. SCE][^13b^, ^20^] and the trifluoromethylsulfonyl radical that fragments SO_2_ to give the trifluoromethyl radical.^[22a]^ The Ir^II^‐complex then reduces via SET the acylazolium intermediate **I** (*E*_1/2_=−1.29 V vs. SCE),[^12^, ^23^] itself generated in situ from benzoyl fluoride and the NHC to provide the persistent ketyl radical **II** along with the starting Ir^III^‐complex closing the photoredox cycle. Meanwhile, the trifluoromethyl radical can add to the double bond of styrene to generate the transient benzylic radical **III**. Subsequently, a radical/radical cross coupling between the persistent ketyl radical **II** and transient C‐radical **III** steered by the persistent radical effect^[24]^ leads to the NHC‐bound intermediate **IV**. NHC‐fragmentation eventually affords the isolated product ketone, thereby closing the NHC catalysis cycle. We currently exclude an alternative mechanism where the benzylic radical **III** gets reduced by the photo catalyst to the corresponding anion that is acylated with the azolium **I** in an ionic process, because the cascade does not proceed upon replacing benzoyl fluoride and **F** by acetone that should react via the benzylic anion if formed to the alcohol **8**, that was not identified (see Scheme 4, equation 4).^[25]^

**Scheme 4:**
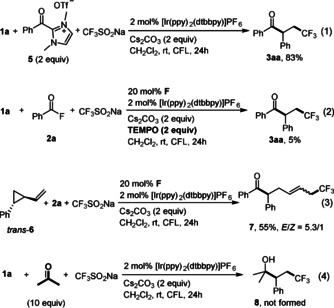
Mechanistic investigations.

**Scheme 5:**
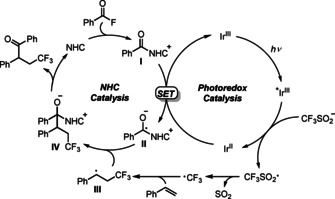
Plausible reaction mechanism.

In summary, a radical alkene acyltrifluoromethylation cascade was developed that operates by cooperative photoredox/NHC catalysis. SET‐*reduction* of readily generated acylazolium intermediates to give ketyl‐type radicals has not been well explored in synthetic radical chemistry.^[12]^ In contrast, *oxidation* of Breslow intermediates to provide similar ketyl‐type intermediates is meanwhile rather well investigated. We show our complementary “reductive” approach can be applied to realize radical acyltrifluoromethylation of styrenes with readily available starting materials. The method allows the preparation of a variety of useful β‐trifluoromethyl‐α‐substituted ketones with good tolerance of functional groups under mild conditions. Since the overall cascade represents a redox‐neutral process, neither an oxidant nor a reductant is necessary. Importantly, since our complementary “reductive” strategy provides the ketyls via SET‐reduction the involved C‐radicals are generated via SET‐oxidation. Considering the many options for oxidative radical generation, our approach opens novel avenues to conduct radical NHC‐catalysis.

## Conflict of interest

The authors declare no conflict of interest.

## Supporting information

As a service to our authors and readers, this journal provides supporting information supplied by the authors. Such materials are peer reviewed and may be re‐organized for online delivery, but are not copy‐edited or typeset. Technical support issues arising from supporting information (other than missing files) should be addressed to the authors.

Supplementary
