# Supplementary material for: Cooperative NHC and Photoredox Catalysis for the Synthesis of β‐Trifluoromethylated Alkyl Aryl Ketones
Source: Angew Chem Int Ed Engl. 2020 Sep 1;59(45):19956–60. doi: 10.1002/anie.202008040 (PMC7693039; doi:10.1002/anie.202008040)
Supplement: Supplementary file 1 — Supplementary [file ANIE-59-19956-s001.pdf]

Angewandte Chemie
Eine Zeitschrift der Gesellschaft Deutscher Chemiker

Supporting Information

Cooperative NHC and Photoredox Catalysis for the Synthesis of β-Trifluoromethylated Alkyl Aryl Ketones

*Qing-Yuan Meng+, Nadine Döben+, and Armido Studer**

anie_202008040_sm_miscellaneous_information.pdf

Table of Contents

1. General information S-2

2. Experimental procedures S-3

3. Optimization conditions S-7

4. Synthesis of substrates and characterization data S-8

5. Characterization data for all products S-14

6. ^{1}H, ^{13}C and ^{19}F NMR spectra S-29

7. References S-80

1. General information

All reactions involving air- or moisture-sensitive reagents or intermediates were carried out in pre-heated glassware under an argon atmosphere using standard *Schlenk* techniques. All styrene derivatives were distilled prior to use, other commercially available reagents were purchased from TCI, Sigma-Aldrich, Alfa Aesar, Acros, ABCR or BLD pharm in the highest purity grade and used directly without further purification. Thin layer chromatography (TLC) was performed on Merck silica gel 60 F-254 plates and visualized by fluorescence quenching under UV light or staining with the standard solution of $KMnO_4$. Column chromatography was performed on Merck or Fluka silica gel 60 (40-63 µm) using a forced flow of 0.5 bar. ^{1}H NMR, ^{13}C NMR and ^{19}F NMR spectra were recorded on *DPX* 300, *AV 400 or 600* at 300 K. Chemical shifts were expressed in parts per million (ppm) with respect to the residual solvent peak. Coupling constants were reported as Hertz (Hz), signal shapes and splitting patterns were indicated as follows: s, singlet; brs, broad singlet; d, doublet; t, triplet; q, quartet; m, multiplet. Mass spectra were recorded on a *Finnigan MAT 4200S*, a *Bruker Daltonics Micro Tof*, a *Waters-Micromass Quatro LCZ* (ESI); peaks are given in *m/z* (% of basis peak). Reactions were performed with compact fluorescent lamp (23 W, Philips). IR spectra were recorded on a Digilab 3100 FT-IR Excalibur Series spectrometer, signal intensities are categorized in strong (s), middle (m) and weak (w). Melting points were measured on a Stuart SMP10 and are uncorrected

2. Experimental procedures

Synthesis of triazolium salt F

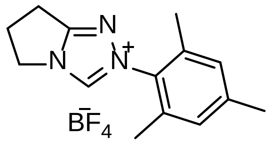

Following a reported procedure,[1] to a stirred solution of pyrrolidin-2-one (1.04 mL, 13.6 mmol) in anhydrous CH_2Cl_2 (50 mL) was added trimethyloxonium tetrafluoroborate (2.06 g, 13.92 mmol) and the mixture stirred at room temperature for 18 hours. Mesitylhydrazine (2.12 g, 13.92 mmol) was then added and the resulting orange solution stirred for a further 18 hours at room temperature. The mixture was then concentrated in vacuo and EtOAc (50 mL) was added. The solid was collected by filtration, washed with EtOAc (3 x 20 mL), then dried under vacuum to give the hydrazone as an off-white solid (2.03 g, 49%). To the hydrazone was added chlorobenzene (14 mL) and triethylorthoformate (6.0 mL, 36.12 mmol) and the mixture then heated at 120 °C for 72 hours. The mixture was concentrated in vacuo then EtOAc (30 mL) was added and the solid collected by filtration, washed with EtOAc (3 x 20 mL), then dried under vacuum to give 2-mesityl-6,7-dihydro-5H-pyrrolo[2,1-*c*][1,2,4]triazol-2-ium tetrafluoroborate as a an off-white solid (1.57 g, 75%). ^{1}H NMR (300 MHz, $CDCl_3$) δ (ppm) 9.39 (s, 1H), 6.96 (s, 2H), 4.73 – 4.49 (m, 2H), 3.21 (t, *J* = 7.7 Hz, 2H), 2.85 (p, *J* = 7.6 Hz, 2H), 2.34 (s, 3H), 2.04 (s, 6H). ^{13}C NMR (75 MHz, $CDCl_3$) δ (ppm) 163.0, 141.9, 141.3, 135.4, 132.0, 129.7, 47.9, 26.7, 22.0, 21.3, 17.2. Spectroscopic data are in accordance with those described in literature.[1]

Synthesis of 2-benzoyl-1,3-dimethyl-1H-imidazol-3-ium trifluoromethanesulfonate

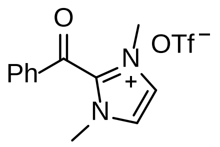

Following a reported procedure,[2,3] a round-bottom flask was charged with 1-methylimidazole (820 mg, 10 mmol) and acetonitrile (10 mL). The stirred solution was cooled to 0 °C and benzoyl chloride (1.41 g, 10 mmol) was added from a dropping funnel within a period of 5 minutes, followed by addition of triethylamine (1.01 g, 10 mmol). The mixture was stirred overnight at ambient temperature, resulting in a yellow solution and a white precipitate of triethylammonium chloride. The ammonium salt was filtered off, solvents and other volatile materials were removed on a rotary evaporator. After that, distillation under vacuum (0.1 – 0.5 mbar) gave a yellow viscous liquid, which was used directly for the next step. To a solution of imidazole (558 mg, 3.0 mmol, 1 equiv) in anhydrous diethyl ether (20 mL) was added methyl triflate (541 mg, 3.3 mmol) at room temperature and the reaction mixture was stirred overnight. The resulting white precipitate

was filtered off and washed with diethyl ether (3 x 10 mL). Drying under vacuum afforded the corresponding product as a white solid (956 mg, 91%). ^{1}H NMR (300 MHz, DMSO-d_6) δ (ppm) 7.99 (s, 2H), 7.97 – 7.91 (m, 2H), 7.87 (ddt, J = 8.8, 7.0, 1.3 Hz, 1H), 7.73 – 7.62 (m, 2H), 3.80 (s, 6H). ^{13}C NMR (75 MHz, DMSO) δ (ppm) 180.4, 138.4, 136.0, 134.7, 130.2, 129.5, 125.4, 37.2. Spectroscopic data are in accordance with those described in literature.[4]

Synthesis of (2-vinylcyclopropyl)benzene (6)

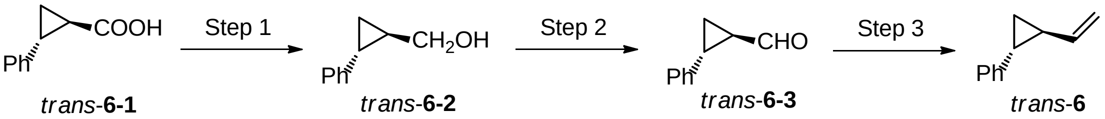

Following a reported procedure,[5] **Step 1**: An oven-dried 100 mL three-necked flask equipped with a stir bar was charged with *trans*-2-phenylcyclopropane-1-carboxylic acid (*trans*-**6-1**) (1 equiv, 10.0 mmol, 1.62 g). Anhydrous THF (20 mL) was added and the suspension was cooled to 0 °C. BH_3•THF (1.22 equiv, 12.2 mmol, 1.0 M solution in THF, 12.2 mL) was added very slowly in a dropwise fashion. The mixture was stirred at 0 °C for 1 hour, after which point it was stirred at room temperature for 1 hour. TLC indicated starting material remained and additional BH_3•THF (4.42 mL, 4.42 mmol, 0.440 equiv) was thus added at room temperature and the mixture was allowed to stir an additional 1.5 hours. The reaction was then quenched at 0 °C with the addition of MeOH in a slow, dropwise fashion. Deionized water was next added until two layers form. The mixture was transferred to a separatory funnel with Et_2O. Brine was added, and the aqueous layer was washed with Et_2O (3 x 25 mL). The combined organic layers were washed with brine (2 x 20 mL). The combined organic layers were then dried with anhydrous sodium sulfate, filtered, and the solvent was removed under reduced pressure with the aid of a rotary evaporator to provide the crude product (*trans*-**6-2**), which was used directly in the next step without further purification.

Step 2: A 250-mL round-bottomed flask equipped with a stir bar was charged with crude *trans*-2-phenylcyclopropyl)methanol (*trans*-**6-2**) from the previous step CH_2Cl_2 (25 mL). The mixture was cooled to 0 °C and TEMPO (0.1 mmol, 16 mg) was added. An aqueous solution of KBr and $NaHCO_3$ (KBr, 11.0 mmol, 1.30 g; $NaHCO_3$, 33.0 mmol, 2.77 g; in 56 mL H_2O) was added and stirring was engaged. Sodium hypochlorite solution (14%, 14 mL) was next added and the red biphasic mixture was stirred 5 min. Then an additional 5 mL sodium hypochlorite solution was added. The mixture was stirred for 1 hour at 0 °C and then quenched with saturated sodium thiosulfate. The layers were separated with the aid of a separatory funnel and the aqueous layer was washed with CH_2Cl_2 (3 x 25 mL). The combined organic layers were dried with anhydrous magnesium sulfate, filtered, and the solvent was removed under reduced pressure with a rotary evaporator to afford the crude product (*trans*-**6-3**) which was used in the next step without further purification.

Step 3: A 250-mL oven-dried round-bottomed flask equipped with a stir bar was charged with

methyltriphenylphosphonium bromide (10.0 mmol, 3.57 g). The flask was placed under an atmosphere of nitrogen using standard Schlenk technique and anhydrous THF (72 mL) was added through the septum. The suspension was cooled to 0 °C with stirring and n-butyllithium (10.0 mmol, 1.6 M in hexanes, 6.32 mL) was added in a dropwise fashion. The cooling bath was removed, and the mixture was stirred at room temperature for 30 min. Next, a solution of crude *trans*-2- phenylcyclopropane-1-carbaldehyde (*trans*-**6-3**) (10.0 mmol, 1.46 g) in THF (9 mL) was added through the septum at room temperature. The mixture was stirred at room temperature for 20 hours. The mixture was quenched at room temperature with saturated aqueous ammonium chloride. The aqueous layer was washed with CH_2Cl_2 (3 x 50 mL). The combined organic layers are dried with anhydrous sodium sulfate, filtered, and the solvent was removed under reduced pressure with the aid of a rotary evaporator. The crude product was purified via flash column chromatography on silica (100% pentane) to provide pure *trans*-(2-vinylcyclopropyl)benzene (*trans*-**6**) (937 mg, 6.5 mmol, 65% over 3 steps) as a colorless liquid. ^{1}H NMR (300 MHz, $CDCl_3$) δ (ppm) 7.38 – 7.27 (m, 2H), 7.25 – 7.17 (m, 1H), 7.16 – 7.09 (m, 2H), 5.60 (ddd, *J* = 17.0, 10.2, 8.4 Hz, 1H), 5.16 (dd, *J* = 17.0, 1.6 Hz, 1H), 4.99 (dd, *J* = 10.2, 1.6 Hz, 1H), 1.98 (ddd, *J* = 8.8, 5.7, 4.3 Hz, 1H), 1.76 (tdd, *J* = 8.5, 5.6, 4.3 Hz, 1H), 1.25 (ddd, *J* = 8.5, 5.7, 5.0 Hz, 1H), 1.16 (dt, *J* = 8.7, 5.3 Hz, 1H). ^{13}C NMR (75 MHz, $CDCl_3$) δ (ppm) 142.4, 140.8, 128.5, 125.8, 125.8, 112.7, 27.5, 25.4, 16.9. Spectroscopic data are in accordance with those described in literature.[5]

General procedure A for the synthesis of acyl fluorides from acyl chlorides[6]

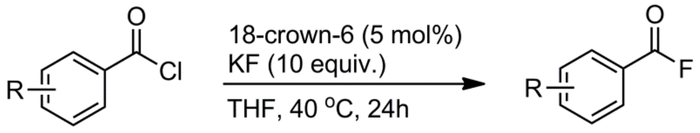

To a 100-mL Schlenk tube with a magnetic stir bar, were successively added acyl chlorides (1 equiv.), 18-crown-6 (5 mol %), KF (10 equiv) and THF (0.2 M). After the reaction was stirred at 40 ºC for 24 hours, the insoluble inorganic solid (KF or KCl) was filtered, and the volatiles were removed using a rotary evaporator. The crude product was purified by bulb-to-bulb distillation to afford the corresponding acyl fluorides.

General procedure B for the synthesis of β-trifluoromethylketones

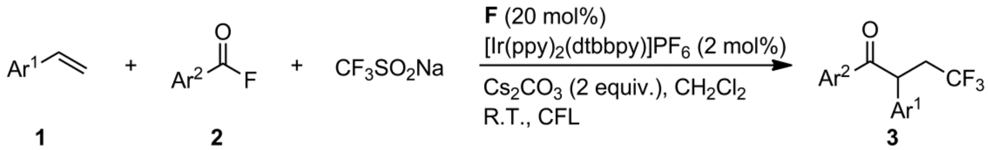

1 **2** **3**

To a Schlenk tube were added Langlois reagent (0.20 mmol), carbene catalyst **F** (6.3 mg, 0.02 mmol), $[Ir(ppy)_2(dtbbpy)]PF_6$ (1.8 mg, 0.002) and Cs_2CO_3 (65.2 mg, 0.2 mmol). Then the reaction tube was evacuated and backfilled with argon for two times. Subsequently, a styrene (0.10 mmol),

an acyl fluoride (0.40 mmol, if solid, it should be added at the beginning) and CH_2Cl_2 (2 mL) were added. The resulting mixture was degassed under vacuum for two times and then it was irradiated with CFL at room temperature for 24 hours. After that, the residue was purified by silica gel chromatography using the mixture of n-hexane and ethyl acetate or pentane and diethylether as an eluent to get the desired product. Each reaction was carried out twice and the average value was used as the final yield.

3. Optimization conditions

Table S1. Solvent screen for the synthesis of 4,4,4-trifluoro-1,2-diphenylbutan-1-one[a]

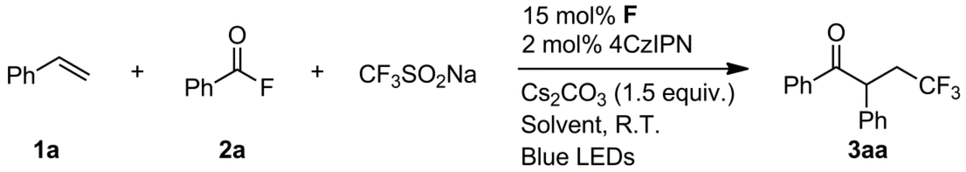

Entry	Solvent	Product (%)[b]
1	THF	9
2	CH_3CN	35
3	DMSO	14
4	DMF	21
5	1,4-dioxane	12
6	acetone	34
7	toluene	3

[a] Unless otherwise noted, all the reactions were carried out with benzoyl fluoride (0.3 mmol), styrene (0.15 mmol), CF_3SO_2Na (0.2 mmol), **F** (0.018 mmol), Cs_2CO_3 (0.225 mmol), and 4CzIPN (0.003 mmol) in the corresponding anhydrous solvent (2 mL), irradiation with blue LEDs at room temperature for 24 hours. **[**b] GC-FID yield using 1,3,5-trimethoxybenzene as an internal standard. **F** = 2-mesityl-6,7-dihydro-5H-pyrrolo[2,1-c][1,2,4]triazol-2-ium tetrafluoroborate. 4CzIPN = 2,4,5,6-tetra(carbazol-9-yl)isophthalonitrile.

4. Synthesis of substrates and characterization data

4-Methoxybenzoyl fluoride

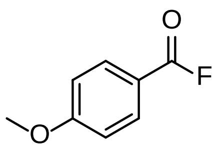

The reaction was performed according to general procedure A with 4-methoxybenzoyl chloride (1.70 g, 10 mmol), 18-crown-6 (132 mg, 0.5 mmol) and KF (5.8 g, 100 mmol) in THF (50 mL). After purification by bulb-to-bulb distillation, 4-methoxybenzoyl fluoride was obtained as a colorless oil (1.16 g, 75% yield). ^{1}H NMR (300 MHz, $CDCl_3$) δ (ppm) 8.09 – 7.84 (m, 2H), 7.09 – 6.81 (m, 2H), 3.88 (s, 3H). ^{13}C NMR (75 MHz, $CDCl_3$) δ (ppm) 165.3, 157.3 (d, $J_{C\text{-}F}$ = 337.5 Hz), 133.8 (d, $J_{C\text{-}F}$ = 4.1 Hz), 117.0 (d, $J_{C\text{-}F}$ = 61.6 Hz), 114.5 (d, $J_{C\text{-}F}$ = 1.6 Hz), 55.7. ^{19}F NMR (282 MHz, $CDCl_3$) δ (ppm) 15.90. Spectroscopic data are in accordance with those described in literature.[6]

4-Fluorobenzoyl fluoride

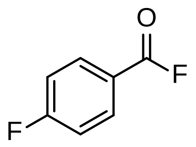

4-Fluorobenzoyl fluoride was prepared starting from the corresponding acid. 4-Fluorobenzoic acid (1.40 g, 10 mmol) and thionyl chloride (1.45 mL, 20 mmol) were heated at reflux in dry toluene (40 mL). After evaporation under vacuum the crude product was used in general procedure A with 18-crown-6 (132 mg, 0.5 mmol) and KF (5.8 g, 100 mmol) in THF (50 mL). After purification by bulb-to-bulb distillation, 4-fluorobenzoyl fluoride was obtained as a colorless oil (527 mg, 37% yield). ^{1}H NMR (300 MHz, $CDCl_3$) δ (ppm) 8.14 – 8.07 (m, 2H), 7.28 – 7.19 (m, 2H). ^{13}C NMR (75 MHz, $CDCl_3$) δ (ppm) 167.3 (d, $J_{C\text{-}F}$ = 255.8 Hz), 156.5 (d, $J_{C\text{-}F}$ = 341.2 Hz), 134.4 (dd, $J_{C\text{-}F}$ = 4.1, 9.9 Hz), 121.0 (dd, $J_{C\text{-}F}$ = 3.4, 62.3 Hz), 116.5 (dd, $J_{C\text{-}F}$ = 22.6 Hz). ^{19}F NMR (282 MHz, $CDCl_3$) δ (ppm) 17.86, -100.73. Spectroscopic data are in accordance with those described in literature.[7]

4-Chlorobenzoyl fluoride

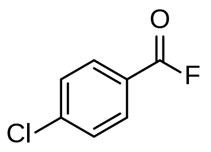

The reaction was performed according to general procedure A with 4-chlorobenzoyl chloride (1.74 g, 10 mmol), 18-crown-6 (132 mg, 0.5 mmol) and KF (5.8 g, 100 mmol) in THF (50 mL).

After purification by bulb-to-bulb distillation, 4-chlorobenzoyl fluoride was obtained as a white solid (1.20 g, 76% yield). ^{1}H NMR (300 MHz, $CDCl_3$) δ (ppm) 8.20 – 7.82 (m, 2H), 7.50 (dd, *J* = 8.6, 1.4 Hz, 2H). ^{13}C NMR (75 MHz, $CDCl_3$) δ (ppm) 156.6 (d, $J_{C\text{-}F}$ = 341.3 Hz), 142.3, 132.8 (d, $J_{C\text{-}F}$ = 3.8 Hz), 129.7, 123.5 (d, $J_{C\text{-}F}$ = 47.3 Hz). ^{19}F NMR (282 MHz, $CDCl_3$) δ (ppm) 18.40. Spectroscopic data are in accordance with those described in literature.[8]

4-Bromobenzoyl fluoride

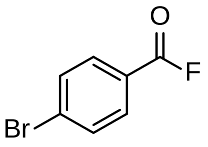

The reaction was performed according to general procedure A with 4-bromobenzoyl chloride (2.18 g, 10 mmol), 18-crown-6 (132 mg, 0.5 mmol) and KF (5.8 g, 100 mmol) in THF (50 mL). After purification by bulb-to-bulb distillation, 4-bromobenzoyl fluoride was obtained as a white solid (1.73 g, 86% yield). ^{1}H NMR (300 MHz, $CDCl_3$) δ (ppm) 7.96 – 7.83 (m, 2H), 7.74 – 7.61 (m, 2H). ^{13}C NMR (75 MHz, $CDCl_3$) δ (ppm) 156.8 (d, $J_{C\text{-}F}$ = 341.6 Hz), 132.8 (d, $J_{C\text{-}F}$ = 4.0 Hz), 132.7, 131.1, 124.0 (d, $J_{C\text{-}F}$ = 62.2 Hz). ^{19}F NMR (282 MHz, $CDCl_3$) δ (ppm) 18.43. Spectroscopic data are in accordance with those described in literature.[9]

4-Iodobenzoyl fluoride

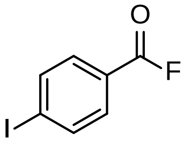

The reaction was performed according to general procedure A with 4-iodobenzoyl chloride (2.66 g, 10 mmol), 18-crown-6 (132 mg, 0.5 mmol) and KF (5.8 g, 100 mmol) in THF (50 mL). After purification by bulb-to-bulb distillation, 4-iodobenzoyl fluoride was obtained as a white solid (1.31 g, 52% yield). ^{1}H NMR (300 MHz, $CDCl_3$) δ (ppm) 7.99 – 7.83 (m, 2H), 7.79 – 7.66 (m, 2H). ^{13}C NMR (75 MHz, $CDCl_3$) δ (ppm) 157.1 (d, $J_{C\text{-}F}$ = 341.6 Hz), 138.7, 132.6 (d, $J_{C\text{-}F}$ = 3.8 Hz), 124. 5 (d, $J_{C\text{-}F}$ = 62.1 Hz), 104.1. ^{19}F NMR (282 MHz, $CDCl_3$) δ (ppm) 18.31.

4-(Trifluoromethoxy)benzoyl fluoride

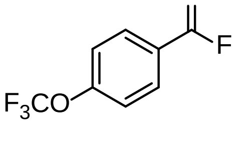

The reaction was performed according to general procedure A with 4-(trifluoromethoxy)benzoyl chloride (2.24 g, 10 mmol), 18-crown-6 (132 mg, 0.5 mmol) and KF (5.8 g, 100 mmol) in THF (50 mL). After purification by bulb-to-bulb distillation, 4-(trifluoromethoxy)benzoyl fluoride was

obtained as a colorless oil (1.08 g, 52% yield). ^{1}H NMR (300 MHz, $CDCl_3$) δ (ppm) 8.11 (d, J = 8.8 Hz, 2H), 7.36 (dq, J = 7.8, 1.2 Hz, 2H). ^{13}C NMR (75 MHz, $CDCl_3$) δ (ppm) 156.3 (d, J_{C-F} = 341.3 Hz), 154.5, 133.7 (d, J_{C-F} = 3.8 Hz), 123.4 (d, J_{C-F} = 62.7 Hz), 120.8, 120.4 (q, J_{C-F} = 258.1 Hz). ^{19}F NMR (282 MHz, $CDCl_3$) δ (ppm) 18.66, -57.65.

4-Cyanobenzoyl fluoride

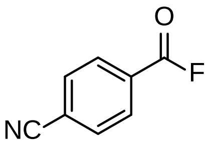

The reaction was performed according to general procedure A with 4-cyanobenzoyl chloride (1.65 g, 10 mmol), 18-crown-6 (132 mg, 0.5 mmol) and KF (5.8 g, 100 mmol) in THF (50 mL). After purification by bulb-to-bulb distillation, 4-cyanobenzoyl fluoride was obtained as a white solid (1.18 g, 79% yield). ^{1}H NMR (300 MHz, $CDCl_3$) δ (ppm) 8.26 – 8.09 (m, 2H), 7.92 – 7.75 (m, 2H). ^{13}C NMR (75 MHz, $CDCl_3$) δ (ppm) 155.8 (d, J_{C-F} = 343.8 Hz), 132.9, 131.9 (d, J_{C-F} = 4.1 Hz), 128.9 (d, J_{C-F} = 63.2 Hz), 118.9, 117.3. ^{19}F NMR (282 MHz, $CDCl_3$) δ (ppm) 20.22. Spectroscopic data are in accordance with those described in literature.[6]

3-Methylbenzoyl fluoride

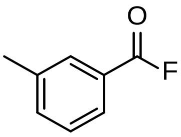

The reaction was performed according to general procedure A with 3-methylbenzoyl chloride (1.54 g, 10 mmol), 18-crown-6 (132 mg, 0.5 mmol) and KF (5.8 g, 100 mmol) in THF (50 mL). After purification by bulb-to-bulb distillation, 3-methylbenzoyl fluoride was obtained as a colorless oil (0.77 g, 56% yield). ^{1}H NMR (300 MHz, $CDCl_3$) δ (ppm) 7.85 (d, J = 6.7 Hz, 2H), 7.51 (d, J = 7.4 Hz, 1H), 7.41 (t, J = 7.4 Hz, 1H), 2.43 (s, 3H). ^{13}C NMR (75 MHz, $CDCl_3$) δ (ppm) 157.7 (d, J_{C-F} = 342.1 Hz), 139.2, 136.2, 132.0 (d, J_{C-F} = 4.1 Hz), 129.0 (d, J_{C-F} = 1.1 Hz), 128.7 (d, J_{C-F} = 3.8 Hz), 125.0 (d, J_{C-F} = 59.8 Hz), 21.3. ^{19}F NMR (282 MHz, $CDCl_3$) δ (ppm) 18.25. Spectroscopic data are in accordance with those described in literature.[7]

3-Bromobenzoyl fluoride

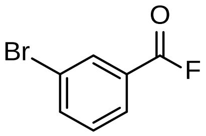

The reaction was performed according to general procedure A with 3-bromobenzoyl chloride (2.18 g, 10 mmol), 18-crown-6 (132 mg, 0.5 mmol) and KF (5.8 g, 100 mmol) in THF (50 mL).

After purification by bulb-to-bulb distillation, 3-bromobenzoyl fluoride was obtained as a white solid (1.26 g, 62% yield). ^{1}H NMR (300 MHz, $CDCl_3$) δ (ppm) 8.16 (t, J = 1.9 Hz, 1H), 7.98 (dt, J = 7.8, 1.3 Hz, 1H), 7.82 (ddd, J = 8.0, 2.0, 1.1 Hz, 1H), 7.42 (td, J = 7.9, 1.4 Hz, 1H). ^{13}C NMR (75 MHz, $CDCl_3$) δ (ppm) 156.1 (d, J_{C-F} = 342.9 Hz), 138.4, 134.3 (d, J_{C-F} = 3.8 Hz), 130.7, 130.0 (d, J_{C-F} = 3.8 Hz), 127.0 (d, J_{C-F} = 62.1 Hz), 123.2. ^{19}F NMR (282 MHz, $CDCl_3$) δ (ppm) 19.07.

2-Fluorobenzoyl fluoride

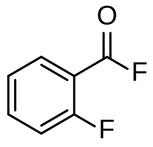

The reaction was performed according to general procedure A with 2-fluorobenzoyl chloride (1.58 g, 10 mmol), 18-crown-6 (132 mg, 0.5 mmol) and KF (5.8 g, 100 mmol) in THF (50 mL). After purification by bulb-to-bulb distillation, 2-fluorobenzoyl fluoride was obtained as a colorless oil (1.16 g, 82% yield). ^{1}H NMR (300 MHz, $CDCl_3$) δ (ppm) 8.05 – 7.89 (m, 1H), 7.74 – 7.67 (m, 1H), 7.41 – 7.15 (m, 2H). ^{13}C NMR (75 MHz, $CDCl_3$) δ (ppm) 163.3 (dd, J_{C-F} = 1.8, 263.4 Hz), 154.0 (dd, J_{C-F} = 3.5, 341.1 Hz), 137.5 (d, J_{C-F} = 9.3 Hz), 133.5 (d, J_{C-F} = 3.3 Hz), 124.8 (d, J_{C-F} = 3.6 Hz), 117.6 (dd, J_{C-F} = 2.3, 21.3 Hz), 113.5 (dd, J_{C-F} = 9.0, 61.8 Hz). ^{19}F NMR (282 MHz, $CDCl_3$) δ (ppm) 31.51 (d, J_{F-F} = 40.8 Hz), -106.41 (d, J_{F-F} = 40.8 Hz).

2-Bromobenzoyl fluoride

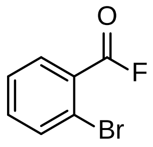

The reaction was performed according to general procedure A with 2-bromobenzoyl chloride (2.18 g, 10 mmol), 18-crown-6 (132 mg, 0.5 mmol) and KF (5.8 g, 100 mmol) in THF (50 mL). After purification by bulb-to-bulb distillation, 2-bromobenzoyl fluoride was obtained as a white solid (1.70 g, 84% yield). ^{1}H NMR (300 MHz, $CDCl_3$) δ (ppm) 8.08 – 7.96 (m, 1H), 7.85 – 7.71 (m, 1H), 7.56 – 7.40 (m, 2H). ^{13}C NMR (75 MHz, $CDCl_3$) δ (ppm) 154.9 (d, J_{C-F} = 342.6 Hz), 135.6 (d, J_{C-F} = 3.5 Hz), 135.4, 133.8 (d, J_{C-F} = 2.1 Hz), 127.8, 125.7 (d, J_{C-F} = 61.2 Hz), 124.9 (d, J_{C-F} = 4.6 Hz). ^{19}F NMR (282 MHz, $CDCl_3$) δ (ppm) 31.59.

2-(*tert*-butyl)benzoyl fluoride

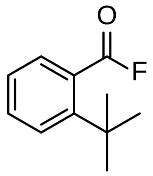

The reaction was performed according to general procedure A with 2-(*tert*-butyl)benzoyl chloride (980 mg, 5 mmol),[10] 18-crown-6 (66 mg, 0.25 mmol) and KF (2.9 g, 50 mmol) in THF (25 mL). After purification by bulb-to-bulb distillation, 2-(*tert*-butyl)benzoyl fluoride was obtained as a colorless oil (567 mg, 63% yield). ^{1}H NMR (300 MHz, $CDCl_3$) δ (ppm) 7.63 – 7.55 (m, 2H), 7.51 (ddd, J = 8.3, 7.1, 1.5 Hz, 1H), 7.34 – 7.27 (m, 1H), 1.48 (s, 9H). ^{13}C NMR (75 MHz, $CDCl_3$) δ (ppm) 160.2 (d, J_{C-F} = 350.1 Hz), 150.4 (d, J_{C-F} = 3.6 Hz), 132.5, 130.6, 127.6 (d, J_{C-F} =2.4 Hz), 126.6 (d, J_{C-F} = 58.5 Hz), 125.9, 36.0, 31.3. ^{19}F NMR (282 MHz, $CDCl_3$) δ (ppm) -248.5.

2-Naphthoyl fluoride

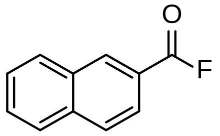

The reaction was performed according to general procedure A with 2-naphthoyl chloride (1.90 g, 10 mmol), 18-crown-6 (132 mg, 0.5 mmol) and KF (5.8 g, 100 mmol) in THF (50 mL). After purification by bulb-to-bulb distillation, 2-naphthoyl fluoride was obtained as a white solid (1.27 g, 73% yield). ^{1}H NMR (300 MHz, $CDCl_3$) δ (ppm) 8.64 (d, J = 1.8 Hz, 1H), 8.08 – 7.86 (m, 4H), 7.71 – 7.59 (m, 2H). ^{13}C NMR (75 MHz, $CDCl_3$) (ppm) 157.8 (d, J_{C-F} = 342.7 Hz), 136.6, 134.2 (d, J_{C-F} = 3.3 Hz), 132.5, 129.82, 129.79, 129.2 (d, J_{C-F} = 1.6 Hz), 128.1, 127.5, 125.8 (d, J_{C-F} = 4.1 Hz), 122.2 (d, J_{C-F} = 60.3 Hz). ^{19}F NMR (282 MHz, $CDCl_3$) δ (ppm) 18.09. Spectroscopic data are in accordance with those described in literature.[9]

Furan-2-carbonyl fluoride

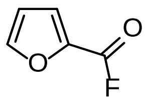

Furan-2-carbonyl fluoride was prepared starting from the corresponding acid. Furan-2-carboxylic acid (1.12 g, 10 mmol) and thionyl chloride (1.45 mL, 20 mmol) were heated at reflux in dry toluene (40 mL). After evaporation under vacuum the crude product was used in general procedure A with 18-crown-6 (132 mg, 0.5 mmol) and KF (5.8 g, 100 mmol) in THF (50 mL). After purification by bulb-to-bulb distillation, furan-2-carbonyl fluoride was obtained as a canary yellow oil (383 mg, 34% yield). ^{1}H NMR (300 MHz, $CDCl_3$) δ (ppm) 7.77 – 7.71 (m, 1H), 7.42 (dd, J = 0.8, 3.7 Hz, 1H), 6.65 – 6.61 (m, 1H). ^{13}C NMR (75 MHz, $CDCl_3$) (ppm) 149.6 (d, J_{C-F} = 2.7 Hz), 148.5 (d, J_{C-F} = 328.0 Hz), 139.4 (d, J_{C-F} = 90.6 Hz), 123.5, 112.9. ^{19}F NMR (282 MHz, $CDCl_3$) δ (ppm) 15.26.

Thiophene-2-carbonyl fluoride

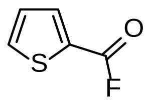

The reaction was performed according to general procedure A with thiophene-2-carbonyl chloride (733 mg, 5.0 mmol), 18-crown-6 (66.1 mg, 0.25 mmol) and KF (2.9 g, 50 mmol) in THF (20 mL). After purification by bulb-to-bulb distillation, thiophene-2-carbonyl fluoride was obtained as a canary yellow oil (424 mg, 65% yield). ^{1}H NMR (300 MHz, $CDCl_3$) δ (ppm) 7.94 (dd, J = 1.3, 3.9 Hz, 1H), 7.81 (ddd, J = 1.3, 2.3, 5.0 Hz, 1H), 7.21 (ddd, J = 2.0, 3.8, 5.0 Hz, 1H). ^{13}C NMR (75 MHz, $CDCl_3$) (ppm) 153.0 (d, J_{C-F} = 2.7 Hz), 137.8 (d, J_{C-F} = 2.7 Hz), 136.8, 128.7, 127.3 (d, J_{C-F} = 2.7 Hz). ^{19}F NMR (282 MHz, $CDCl_3$) δ (ppm) 24.34. Spectroscopic data are in accordance with those described in literature.[11]

5. Characterization data for products

4,4,4-Trifluoro-1,2-diphenylbutan-1-one (3aa)

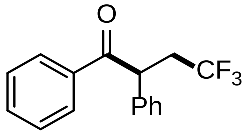

The reaction was performed according to general procedure B with styrene (10.4 g, 0.1 mmol) and benzoyl fluoride (49.6 mg, 0.4 mmol). After purification by flash chromatography (*n*-pentane/ethyl acetate = 50:1), the desired product was obtained as a colorless oil (20.6 mg, 74% yield). ^{1}H NMR (300 MHz, $CDCl_3$) δ (ppm) 7.99 (dd, *J* = 8.4, 1.2 Hz, 2H), 7.57 – 7.48 (m, 1H), 7.48 – 7.38 (m, 2H), 7.34 (d, *J* = 4.4 Hz, 4H), 7.31 – 7.24 (m, 1H), 4.94 (dd, *J* = 7.6, 5.5 Hz, 1H), 3.47 – 3.20 (m, 1H), 2.70 – 2.45 (m, 1H). ^{13}C NMR (75 MHz, $CDCl_3$) δ (ppm) 196.9, 137.6, 135.9, 133.5, 132.1, 129.5, 129.0, 128.8, 128.2, 128.0, 126.6 (q, $J_{C\text{-}F}$ = 275.4 Hz), 47.4 (q, $J_{C\text{-}F}$ = 2.4 Hz), 37.6 (q, $J_{C\text{-}F}$ = 28.0 Hz). ^{19}F {^{1}H} NMR (282 MHz, $CDCl_3$) δ (ppm) -64.59. HRMS (ESI) Calcd. for $C_{16}H_{13}F_3NaO$ $[M+Na]^+$: 301.0816. Found: 301.1411. Spectroscopic data are in accordance with those described in literature.[12]

4,4,4-Trifluoro-1-(4-methoxyphenyl)-2-phenylbutan-1-one (3ab)

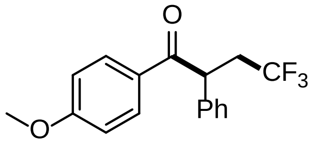

The reaction was performed according to general procedure B with styrene (10.4 g, 0.1 mmol) and 4-methoxybenzoyl fluoride (61.6 mg, 0.4 mmol). After purification by flash chromatography (*n*-pentane/ethyl acetate = 50:1), the desired product was obtained as a colorless oil (20.6 mg, 67% yield). ^{1}H NMR (300 MHz, $CDCl_3$) δ (ppm) 8.05 – 7.89 (m, 2H), 7.41 – 7.19 (m, 5H), 6.97 – 6.83 (m, 2H), 4.88 (dd, *J* = 7.5, 5.7 Hz, 1H), 3.84 (s, 3H), 3.42 – 3.19 (m, 1H), 2.66 – 2.43 (m, 1H). ^{13}C NMR (75 MHz, $CDCl_3$) δ (ppm) 195.3, 163.9, 138.1, 131.3, 129.4, 128.8, 128.1, 127.9, 126.6 (q, $J_{C\text{-}F}$ = 275.5 Hz), 114.0, 55.6, 47.0 (q, $J_{C\text{-}F}$ = 2.5 Hz), 37.5 (q, $J_{C\text{-}F}$ = 27.3 Hz). ^{19}F {^{1}H} NMR (282 MHz, $CDCl_3$) δ (ppm) -64.58. HRMS (ESI) Calcd. for $C_{17}H_{15}F_3NaO_2$ $[M+Na]^+$: 331.0922. Found: 331.0917. Spectroscopic data are in accordance with those described in literature.[12]

4,4,4-Trifluoro-1-(4-fluorophenyl)-2-phenylbutan-1-one (3ac)

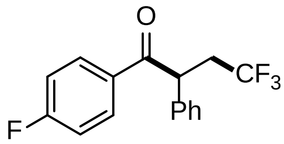

The reaction was performed according to general procedure B with styrene (10.4 g, 0.1 mmol) and

4-fluorobenzoyl fluoride (56.8 mg, 0.4 mmol). After purification by flash chromatography (*n*-pentane/ethyl acetate = 50:1), the desired product was obtained as a colorless oil (26.0 mg, 88% yield). ^{1}H NMR (300 MHz, $CDCl_3$) δ (ppm) 8.07 – 7.93 (m, 2H), 7.29 (qd, J = 6.7, 2.2 Hz, 5H), 7.14 – 7.00 (m, 2H), 4.85 (dd, J = 7.7, 5.4 Hz, 1H), 3.40 – 3.22 (m, 1H), 2.63 – 2.45 (m, 1H). ^{13}C NMR (75 MHz, $CDCl_3$) δ (ppm) 195.3, 165.9 (d, J_{C-F} = 254.0 Hz), 137.5, 132.2 (d, J_{C-F} = 2.9 Hz), 131.6 (d, J_{C-F} = 9.3 Hz), 129.6, 129.3, 128.1, 126.5 (q, J_{C-F} = 275.5 Hz), 116.0 (d, J_{C-F} = 21.9 Hz), 47.4, 37.5 (q, J_{C-F} = 28.1 Hz). ^{19}F {^{1}H} NMR (282 MHz, $CDCl_3$) δ (ppm) -64.62, -104.42. HRMS (ESI) Calcd. for $C_{16}H_{13}ClF_3O$ [M+H]$^+$: 297.0903. Found: 297.0897. Spectroscopic data are in accordance with those described in literature.[12]

1-(4-Chlorophenyl)-4,4,4-trifluoro-2-phenylbutan-1-one (3ad)

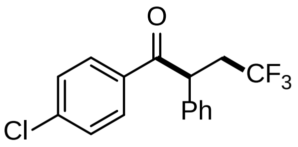

The reaction was performed according to general procedure B with styrene (10.4 g, 0.1 mmol) and 4-chlorobenzoyl fluoride (63.2 mg, 0.4 mmol). After purification by flash chromatography (*n*-pentane/ethyl acetate = 50:1), the desired product was obtained as a white solid (26.5 mg, 85% yield). ^{1}H NMR (300 MHz, $CDCl_3$) δ (ppm) 7.98 – 7.82 (m, 2H), 7.46 – 7.17 (m, 7H), 4.84 (dd, J = 7.7, 5.5 Hz, 1H), 3.40 – 3.21 (m, 1H), 2.63 – 2.45 (m, 1H). ^{13}C NMR (75 MHz, $CDCl_3$) δ (ppm) 195.7, 140.0, 137.3, 134.1, 130.4, 129.6, 129.2, 128.2, 128.1, 126.5 (q, J_{C-F} = 275.3 Hz), 47.5, 37.5 (q, J_{C-F} = 28.1 Hz). ^{19}F {^{1}H} NMR (282 MHz, $CDCl_3$) δ (ppm) -64.59. HRMS (ESI) Calcd. for $C_{16}H_{12}ClF_3NaO$ [M+Na]$^+$: 335.0426. Found: 335.0422. Spectroscopic data are in accordance with those described in literature.[12]

1-(4-Bromophenyl)-4,4,4-trifluoro-2-phenylbutan-1-one (3ae)

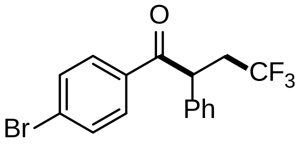

The reaction was performed according to general procedure B with styrene (10.4 g, 0.1 mmol) and 4-bromobenzoyl fluoride (80.8 mg, 0.4 mmol). After purification by flash chromatography (*n*-pentane/ethyl acetate = 50:1), the desired product was obtained as a white solid (28.8 mg, 81% yield). ^{1}H NMR (300 MHz, $CDCl_3$) δ (ppm) 7.87 – 7.75 (m, 2H), 7.61 – 7.50 (m, 2H), 7.35 – 7.21 (m, 5H), 4.83 (dd, J = 7.7, 5.4 Hz, 1H), 3.39 – 3.21 (m, 1H), 2.63 – 2.45 (m, 1H). ^{13}C NMR (75 MHz, $CDCl_3$) δ (ppm) 195.8, 137.2, 134.6, 132.2, 130.4, 129.6, 128.8, 128.2, 128.1, 126.5 (q, J_{C-F} = 275.3 Hz), 47.5 (q, J_{C-F} = 2.5 Hz), 37.4 (q, J_{C-F} = 28.2 Hz). ^{19}F {^{1}H} NMR (282 MHz, $CDCl_3$) δ (ppm) -64.58. HRMS (ESI) Calcd. for $C_{16}H_{12}BrF_3NaO$ [M+Na]$^+$: 378.9921. Found: 378.9917. Spectroscopic data are in accordance with those described in literature.[12]

4,4,4-Trifluoro-1-(4-iodophenyl)-2-phenylbutan-1-one (3af)

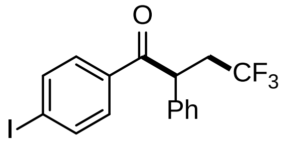

The reaction was performed according to general procedure B with styrene (10.4 g, 0.1 mmol) and 4-iodobenzoyl fluoride (100.0 mg, 0.4 mmol). After purification by flash chromatography (*n*-pentane/ethyl acetate = 50:1), the desired product was obtained as a colorless oil (25.5 mg, 63% yield). ^{1}H NMR (300 MHz, $CDCl_3$) δ (ppm) 7.76 (d, *J* = 8.6 Hz, 2H), 7.65 (d, *J* = 8.6 Hz, 2H), 7.35 – 7.21 (m, 5H), 4.82 (dd, *J* = 7.7, 5.5 Hz, 1H), 3.39 – 3.20 (m, 1H), 2.62 – 2.44 (m, 1H). ^{13}C NMR (75 MHz, $CDCl_3$) δ (ppm) 196.2 138.2, 137.2, 135.1, 130.3, 129.6, 128.2, 128.1, 126.5 (q, J_{C-F} = 275.3 Hz), 101.6, 47.4, 37.5 (q, J_{C-F} = 28.1 Hz). ^{19}F {^{1}H} NMR (282 MHz, $CDCl_3$) δ (ppm) -64.59. HRMS (ESI) Calcd. for $C_{16}H_{12}F_3INaO$ $[M+Na]^+$: 426.9783. Found: 426.9775. IR (neat): 1686, 1581, 1562, 1494, 1455, 1433, 1394, 1383, 1337, 1257, 1212, 1175, 1134, 1113, 1103, 1074, 1058, 1031, 1007, 974, 936, 877, 841, 805, 781, 752, 699, 634, 597.

4,4,4-Trifluoro-2-phenyl-1-(4-(trifluoromethoxy)phenyl)butan-1-one (3ag)

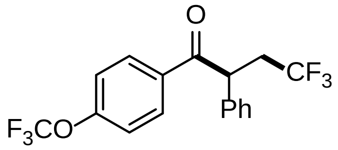

The reaction was performed according to general procedure B with styrene (10.4 g, 0.1 mmol) and 4-(trifluoromethoxy)benzoyl fluoride (83.2 mg, 0.4 mmol). After purification by flash chromatography (*n*-pentane/ethyl acetate = 50:1), the desired product was obtained as a colorless oil (26.8 mg, 74% yield). ^{1}H NMR (300 MHz, $CDCl_3$) δ (ppm) 8.07 – 7.96 (m, 2H), 7.41 – 7.16 (m, 7H), 4.85 (dd, *J* = 7.8, 5.3 Hz, 1H), 3.40 – 3.22 (m, 1H), 2.65 – 2.38 (m, 1H). ^{13}C NMR (75 MHz, $CDCl_3$) δ (ppm) 195.3, 152.9, 137.2, 134.0, 131.0, 129.7, 128.2, 128.1, 126.5 (q, J_{C-F} = 275.3 Hz), 120.5, 120.4 (q, J_{C-F} = 257.9 Hz), 47.6, 37.5 (q, J_{C-F} = 28.1 Hz). ^{19}F {^{1}H} NMR (282 MHz, $CDCl_3$) δ (ppm) -57.64, -64.67. HRMS (ESI) Calcd. for $C_{17}H_{12}F_6NaO_2$ $[M+Na]^+$: 385.0639. Found: 385.0633. IR (neat): 1689, 1603, 1508, 1456, 1384, 1338, 1253, 1208, 1165, 1137, 1111, 1075, 1060, 1031, 1017, 976, 927, 879, 851, 810, 777, 752, 699, 655, 632, 598.

4-(4,4,4-Trifluoro-2-phenylbutanoyl)benzonitrile (3ah)

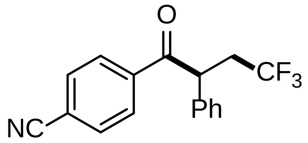

The reaction was performed according to general procedure B with styrene (10.4 g, 0.1 mmol) and 4-cyanobenzoyl fluoride (59.6 mg, 0.4 mmol). After purification by flash chromatography

(*n*-pentane/ethyl acetate = 50:1), the desired product was obtained as a colorless oil (22.1 mg, 73% yield). ^{1}H NMR (300 MHz, $CDCl_3$) δ (ppm) 8.08 – 7.93 (m, 2H), 7.76 – 7.63 (m, 2H), 7.40 – 7.23 (m, 5H), 4.85 (dd, *J* = 7.7, 5.4 Hz, 1H), 3.41 – 3.23 (m, 1H), 2.65 – 2.47 (m, 1H). ^{13}C NMR (75 MHz, $CDCl_3$) δ (ppm) 195.6, 138.9, 136.5, 132.6, 129.8, 129.3, 128.4, 128.1, 126.5 (q, $J_{C\text{-}F}$ = 275.3 Hz), 117.8, 116.7, 47.9 (q, $J_{C\text{-}F}$ = 2.5 Hz), 37.4 (q, $J_{C\text{-}F}$ = 28.3 Hz). ^{19}F {^{1}H} NMR (282 MHz, $CDCl_3$) δ (ppm) -64.60. HRMS (ESI) Calcd. for $C_{17}H_{12}F_3NNaO$ $[M+Na]^+$: 326.0769. Found: 326.0763. IR (neat): 2233, 1690, 1607, 1567, 1493, 1455, 1434, 1407, 1384, 1338, 1287, 1257, 1235, 1212, 1136, 1105, 1075, 1059, 1031, 1018, 976, 938, 880, 849, 820, 767, 746, 700, 638, 600.

4,4,4-Trifluoro-2-phenyl-1-(m-tolyl)butan-1-one (3ai)

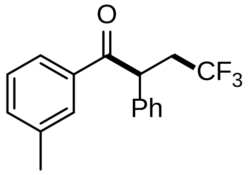

The reaction was performed according to general procedure B with styrene (10.4 g, 0.1 mmol) and 3-methylbenzoyl fluoride (55.2 mg, 0.4 mmol). After purification by flash chromatography (*n*-pentane/ethyl acetate = 50:1), the desired product was obtained as a colorless oil (19.9 mg, 68% yield). ^{1}H NMR (300 MHz, $CDCl_3$) δ (ppm) 7.82 – 7.71 (m, 2H), 7.38 – 7.19 (m, 7H), 4.91 (dd, *J* = 7.8, 5.4 Hz, 1H), 3.40 – 3.22 (m, 1H), 2.63 – 2.45 (m, 1H), 2.36 (s, 3H). ^{13}C NMR (75 MHz, $CDCl_3$) δ (ppm) 197.1, 138.7, 137.7, 135.9, 134.3, 129.5, 129.4, 128.6, 128.2, 127.9, 126.6 (q, $J_{C\text{-}F}$ = 275.3 Hz), 126.2, 47.3, 37.6 (q, $J_{C\text{-}F}$ = 28.0 Hz), 21.5. ^{19}F {^{1}H} NMR (282 MHz, $CDCl_3$) δ (ppm) -64.61. HRMS (ESI) Calcd. for $C_{17}H_{15}F_3NaO$ $[M+Na]^+$: 315.0973. Found: 315.0967. Spectroscopic data are in accordance with those described in literature.[12]

1-(3-Bromophenyl)-4,4,4-trifluoro-2-phenylbutan-1-one (3aj)

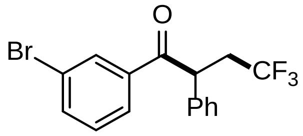

The reaction was performed according to general procedure B with styrene (10.4 g, 0.1 mmol) and 3-bromobenzoyl fluoride (80.8 mg, 0.4 mmol). After purification by flash chromatography (*n*-pentane/ethyl acetate = 50:1), the desired product was obtained as a colorless oil (26.3 mg, 74% yield). ^{1}H NMR (300 MHz, $CDCl_3$) δ (ppm) 8.08 (t, *J* = 1.9 Hz, 1H), 7.86 (dt, *J* = 7.8, 1.4 Hz, 1H), 7.63 (ddd, *J* = 8.0, 2.1, 1.0 Hz, 1H), 7.41 – 7.16 (m, 6H), 4.83 (dd, *J* = 7.8, 5.4 Hz, 1H), 3.39 – 3.21 (m, 1H), 2.63 – 2.45 (m, 1H). ^{13}C NMR (75 MHz, $CDCl_3$) δ (ppm) 195.5, 137.6, 137.0, 136.3, 132.0, 130.3, 129.6, 128.2, 128.1, 127.4, 126.4 (q, $J_{C\text{-}F}$ = 275.5 Hz), 123.2, 47.6 (q, $J_{C\text{-}F}$ = 2.5 Hz), 37.5 (q, $J_{C\text{-}F}$ = 28.1 Hz). ^{19}F {^{1}H} NMR (282 MHz, $CDCl_3$) δ (ppm) -64.60. HRMS (ESI) Calcd.

for $C_{16}H_{13}BrF_3O$ $[M+H]^+$: 357.0102. Found: 357.0096. Spectroscopic data are in accordance with those described in literature.[12]

4,4,4-Trifluoro-2-phenyl-1-(3-(trifluoromethyl)phenyl)butan-1-one (3ak)

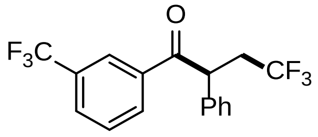

The reaction was performed according to general procedure B with styrene (10.4 g, 0.1 mmol) and 3-(trifluoromethyl)benzoyl fluoride (76.8 mg, 0.4 mmol). After purification by flash chromatography (*n*-pentane/ethyl acetate = 50:1), the desired product was obtained as a colorless oil (23.2 mg, 67% yield). ^{1}H NMR (300 MHz, $CDCl_3$) δ (ppm) 8.21 (d, *J* = 1.8 Hz, 1H), 8.11 (dt, *J* = 7.9, 1.4 Hz, 1H), 7.81 – 7.71 (m, 1H), 7.54 (t, *J* = 7.8 Hz, 1H), 7.42 – 7.24 (m, 5H), 4.87 (dd, *J* = 7.7, 5.4 Hz, 1H), 3.42 – 3.23 (m, 1H), 2.70 – 2.44 (m, 1H). ^{13}C NMR (150 MHz, $CDCl_3$) δ (ppm) 195.5, 136.9, 136.3, 132.0 (d, $J_{C\text{-}F}$ = 1.4 Hz), 131.5 (q, $J_{C\text{-}F}$ = 33.0 Hz), 129.9 (q, $J_{C\text{-}F}$ = 3.6 Hz), 129.7, 129.5, 128.3, 128.1, 126.4 (q, $J_{C\text{-}F}$ = 277.7 Hz), 125.8 (q, $J_{C\text{-}F}$ = 3.9 Hz), 123.7 (q, $J_{C\text{-}F}$ = 273.1 Hz), 47.7 (q, $J_{C\text{-}F}$ = 2.6 Hz), 37.4 (q, $J_{C\text{-}F}$ = 28.4 Hz). ^{13}C {^{19}F} NMR (151 MHz, $CDCl_3$) δ (ppm) 195.5, 136.9, 136.3, 132.0, 131.5, 129.9, 129.7, 129.5, 128.3, 128.1, 126.4, 125.8, 123.7, 47.7, 37.4. ^{19}F {^{1}H} NMR (282 MHz, $CDCl_3$) δ (ppm) -63.01, -64.60. HRMS (ESI) Calcd. for $C_{17}H_{12}F_6NaO$ $[M+Na]^+$: 369.0690. Found: 369.0683. IR (neat): 1692, 1611, 1495, 1436, 1384, 1328, 1257, 1233, 1206, 1170, 1126, 1073, 1032, 993, 980, 947, 872, 835, 798, 751, 692, 652, 599, 579.

4,4,4-Trifluoro-1-(2-fluorophenyl)-2-phenylbutan-1-one (3al)

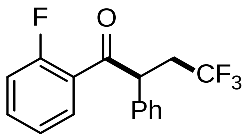

The reaction was performed according to general procedure B with styrene (10.4 g, 0.1 mmol) and 2-fluorobenzoyl fluoride (56.8 mg, 0.4 mmol). After purification by flash chromatography (*n*-pentane/ethyl acetate = 50:1), the desired product was obtained as a colorless oil (21.6 mg, 73% yield). ^{1}H NMR (300 MHz, $CDCl_3$) δ (ppm) 7.77 (td, *J* = 7.6, 1.9 Hz, 1H), 7.50 – 7.41 (m, 1H), 7.36 – 7.21 (m, 5H), 7.17 (td, *J* = 7.6, 1.1 Hz, 1H), 7.07 (ddd, *J* = 11.3, 8.3, 1.1 Hz, 1H), 4.90 (dd, *J* = 7.6, 5.7 Hz, 1H), 3.48 – 3.20 (m, 1H), 2.67 – 2.37 (m, 1H). ^{13}C NMR (75 MHz, $CDCl_3$) δ (ppm) 195.8, 161.2 (d, $J_{C\text{-}F}$ = 253.0 Hz), 136.7, 135.0, 134.8, 131.5, 129.2, 128.6, 128.0, 126.6 (q, $J_{C\text{-}F}$ = 275.3 Hz), 124.7 (d, $J_{C\text{-}F}$ = 3.4 Hz), 116.8 (d, $J_{C\text{-}F}$ = 23.4 Hz), 51.1, 37.2 (q, $J_{C\text{-}F}$ = 28.2 Hz). ^{19}F {^{1}H} NMR (282 MHz, $CDCl_3$) δ (ppm) -64.61, -109.45 (d, $J_{C\text{-}F}$ = 2.5 Hz). HRMS (ESI) Calcd. for $C_{16}H_{12}F_4NaO$ $[M+Na]^+$: 319.0722. Found: 319.0714. IR (neat): 1685, 1610, 1579, 1482, 1452, 1435, 1383, 1382, 1255, 1234, 1208, 1135, 1117, 1075, 1031, 977, 939, 880, 833, 761, 709, 657,

601.

1-(2-Bromophenyl)-4,4,4-trifluoro-2-phenylbutan-1-one (3am)

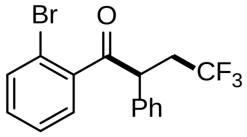

The reaction was performed according to general procedure B with styrene (10.4 g, 0.1 mmol) and 2-bromobenzoyl fluoride (80.8 mg, 0.4 mmol). After purification by flash chromatography (*n*-pentane/ethyl acetate = 50:1), the desired product was obtained as a colorless oil (14.6 mg, 41% yield). ^{1}H NMR (300 MHz, $CDCl_3$) δ (ppm) 7.60 – 7.51 (m, 1H), 7.35 – 7.15 (m, 7H), 7.07 (dt, *J* = 5.2, 3.5 Hz, 1H), 4.78 (t, *J* = 6.6 Hz, 1H), 3.40 – 3.22 (m, 1H), 2.79 – 2.43 (m, 1H). ^{13}C NMR (75 MHz, $CDCl_3$) δ (ppm) 200.1, 140.4, 135.6, 133.8, 131.8, 129.3, 129.2, 128.7, 128.3, 127.2, 126.6 (q, $J_{C\text{-}F}$ = 275.4 Hz), 119.1, 51.5, 36.1 (q, $J_{C\text{-}F}$ = 28.4 Hz). ^{19}F {^{1}H} NMR (282 MHz, $CDCl_3$) δ (ppm) -64.17. HRMS (ESI) Calcd. for $C_{16}H_{12}BrF_3NaO$ $[M+Na]^+$: 378.9921. Found: 378.9919. IR (neat): 1704, 1588, 1493, 1467, 1455, 1430, 1384, 1328, 1285, 1256, 1233, 1209, 1137, 1118, 1065, 1048, 1029, 973, 937, 877, 825, 760, 731, 699, 600.

1-(2-(*tert*-butyl)phenyl)-4,4,4-trifluoro-2-phenylbutan-1-one (3an)

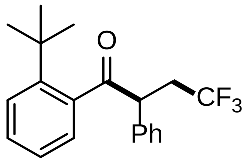

The reaction was performed according to general procedure B with styrene (10.4 g, 0.1 mmol) and 2-(*tert*-butyl)benzoyl fluoride (72.0 mg, 0.4 mmol). After purification by flash chromatography (*n*-pentane/ethyl acetate = 100:1), the desired product was obtained as a colorless oil (15.4 mg, 46% yield). ^{1}H NMR (300 MHz, $CDCl_3$) δ (ppm) 7.48 (dd, *J* = 8.1, 1.2 Hz, 1H), 7.35 – 7.26 (m, 4H), 7.20 (dt, *J* = 6.7, 2.2 Hz, 2H), 7.06 (ddd, *J* = 7.7, 7.3, 1.2 Hz, 1H), 6.81 (dd, *J* = 7.7, 1.5 Hz, 1H), 4.62 (dd, *J* = 7.9, 5.4 Hz, 1H), 3.31 – 3.14 (m, 1H), 2.93 – 2.75 (m, 1H), 1.21 (s, 9H). ^{13}C NMR (75 MHz, $CDCl_3$) δ (ppm) 205.4, 148.5, 139.3, 136.2, 130.0, 129.2, 129.0, 128.2, 128.1, 127.9, 126.6 (q, $J_{C\text{-}F}$ = 275.4 Hz), 125.1, 53.7, 36.7 (q, $J_{C\text{-}F}$ = 28.0 Hz), 36.2, 32.0. ^{19}F {^{1}H} NMR (282 MHz, $CDCl_3$) δ (ppm) -64.10. GC-MS (EI) Calcd. for $C_{20}H_{21}F_3O$ $[M]^+$: 334.15. Found: 334.10. IR (neat): 1703, 1587, 1484, 1381, 1366, 1383, 1253, 1134, 1117, 1073, 1056, 1030, 963, 934, 877, 758, 698, 602.

4,4,4-Trifluoro-2-(naphthalen-2-yl)-1-phenylbutan-1-one (3ao)

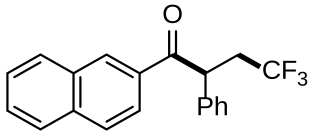

The reaction was performed according to general procedure B with styrene (10.4 g, 0.1 mmol) and 2-naphthoyl fluoride (69.6 mg, 0.4 mmol). After purification by flash chromatography (*n*-pentane/ethyl acetate = 50:1), the desired product was obtained as a colorless oil (15.4 mg, 47% yield). ^{1}H NMR (300 MHz, $CDCl_3$) δ (ppm) 8.50 (d, *J* = 1.7 Hz, 1H), 8.01 (dd, *J* = 8.7, 1.8 Hz, 1H), 7.93 (dd, *J* = 7.8, 1.6 Hz, 1H), 7.87 – 7.77 (m, 2H), 7.55 (dqd, *J* = 8.3, 6.9, 1.5 Hz, 2H), 7.43 – 7.17 (m, 5H), 5.08 (dd, *J* = 7.7, 5.5 Hz, 1H), 3.47 – 3.29 (m, 1H), 2.77 – 2.41 (m, 1H). ^{13}C NMR (75 MHz, $CDCl_3$) δ (ppm) 196.8, 137.7, 135.8, 133.2, 132.6, 130.8, 129.8, 129.5, 128.9, 128.7, 128.2, 128.0, 127.9, 127.0, 126.6 (q, $J_{C\text{-}F}$ = 275.3 Hz), 124.5, 47.4, 37.42 (q, $J_{C\text{-}F}$ = 28.1 Hz). ^{19}F {^{1}H} NMR (282 MHz, $CDCl_3$) δ (ppm) -64.51. HRMS (ESI) Calcd. for $C_{20}H_{15}F_3NaO$ $[M+Na]^+$: 351.0973. Found: 351.0967. Spectroscopic data are in accordance with those described in literature.[12]

4,4,4-Trifluoro-1-(furan-2-yl)-2-phenylbutan-1-one (3ap)

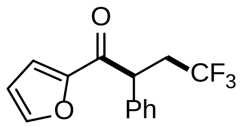

The reaction was performed according to general procedure B with styrene (10.4 g, 0.1 mmol) and furan-2-carbonyl fluoride (45.6 mg, 0.4 mmol). After purification by flash chromatography (*n*-pentane/ethyl acetate = 50:1), the desired product was obtained as a colorless oil (12.3 mg, 46% yield). ^{1}H NMR (300 MHz, $CDCl_3$) δ (ppm) 7.59 – 7.51 (m, 1H), 7.38 – 7.25 (m, 5H), 7.22 (dd, *J* = 3.6, 0.8 Hz, 1H), 6.49 (dd, *J* = 3.6, 1.7 Hz, 1H), 4.73 (dd, *J* = 8.1, 5.4 Hz, 1H), 3.37 – 3.18 (m, 1H), 2.62 – 2.44 (m, 1H). ^{13}C NMR (75 MHz, $CDCl_3$) δ (ppm) 185.8, 151.8, 147.0, 137.2, 129.3, 128.3, 128.0, 126.4 (q, $J_{C\text{-}F}$ = 275.5 Hz), 118.7, 112.7, 47.4, 36.6 (q, $J_{C\text{-}F}$ = 28.3 Hz). ^{19}F {^{1}H} NMR (282 MHz, $CDCl_3$) δ (ppm) -64.83. HRMS (ESI) Calcd. for $C_{14}H_{11}F_3NaO_2$ $[M+Na]^+$: 291.0609. Found: 291.0600. Spectroscopic data are in accordance with those described in literature.[12]

4,4,4-Trifluoro-2-phenyl-1-(thiophen-2-yl)butan-1-one (3aq)

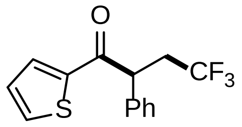

The reaction was performed according to general procedure B with styrene (10.4 g, 0.1 mmol) and thiophene-2-carbonyl fluoride (52.0 mg, 0.4 mmol). After purification by flash chromatography (*n*-pentane/ethyl acetate = 50:1), the desired product was obtained as a colorless oil (11.4 mg, 40% yield). ^{1}H NMR (300 MHz, $CDCl_3$) δ (ppm) 7.74 (dd, *J* = 3.9, 1.1 Hz, 1H), 7.61 (dd, *J* = 4.9, 1.1 Hz, 1H), 7.40 – 7.25 (m, 5H), 7.07 (dd, *J* = 4.9, 3.8 Hz, 1H), 4.70 (dd, *J* = 7.7, 5.5 Hz, 1H), 3.38 – 3.19 (m, 1H), 2.63 – 2.46 (m, 1H). ^{13}C NMR (75 MHz, $CDCl_3$) δ (ppm) 189.7, 142.8, 137.7, 134.6, 133.1, 129.4, 128.4, 128.1, 126.4 (q, $J_{C\text{-}F}$ = 275.5 Hz), 48.8, 37.3 (q, $J_{C\text{-}F}$ = 28.2 Hz). ^{19}F {^{1}H} NMR (282 MHz, $CDCl_3$) δ (ppm) -64.67. HRMS (ESI) Calcd. for $C_{14}H_{11}F_3NaOS$ $[M+Na]^+$:

307.0380. Found: 307.0375. Spectroscopic data are in accordance with those described in literature.[12]

4,4,4-Trifluoro-1-phenyl-2-(p-tolyl)butan-1-one (3ba)

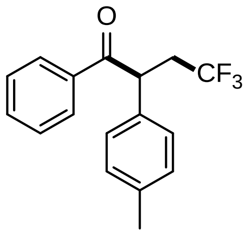

The reaction was performed according to general procedure B with 4-methyl styrene (11.8 mg, 0.1 mmol) and benzoyl fluoride (49.6 mg, 0.4 mmol). After purification by flash chromatography (*n*-pentane/diethyl ether = 40:1), the desired product was obtained as a colorless oil (19.1 mg, 65% yield). ^{1}H NMR (300 MHz, $CDCl_3$, 300 K) δ (ppm) 8.00 – 7.93 (m, 2H), 7.54 – 7.47 (m, 1H), 7.44 – 7.37 (m, 2H), 7.20 (d, J = 8.2 Hz, 2H), 7.12 (d, J = 7.9 Hz, 2H), 4.88 (dd, J = 7.7, 5.5 Hz, 1H), 3.41 – 3.19 (m, 1H), 2.63 – 2.43 (m, 1H), 2.29 (s, 3H). ^{13}C NMR (75 MHz, $CDCl_3$, 300 K) δ (ppm) 197.0, 137.8, 136.0, 134.6, 133.4, 130.2, 129.0, 128.8, 128.1, 126.6 (q, $J_{C\text{-}F}$ = 278.0 Hz), 47.0 (q, $J_{C\text{-}F}$ = 2.5 Hz), 37.6 (q, $J_{C\text{-}F}$ = 28.1 Hz), 21.1. ^{19}F {^{1}H} NMR (282 MHz, $CDCl_3$, 300 K) δ (ppm) -64.57. MS (EI) Calcd. for $C_{17}H_{15}F_3O$ M^+: 292.11. Found: 292.1. Spectroscopic data are in accordance with those described in literature.[13]

4,4,4-Trifluoro-1-phenyl-2-(m-tolyl)butan-1-one (3ca)

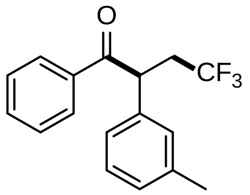

The reaction was performed according to general procedure B with 3-methyl styrene (11.8 mg, 0.1 mmol) and benzoyl fluoride (49.6 mg, 0.4 mmol). After purification by flash chromatography (*n*-pentane/diethyl ether = 40:1), the desired product was obtained as a colorless oil (19.7 mg, 67% yield). ^{1}H NMR (300 MHz, $CDCl_3$, 300 K) δ (ppm) 8.01 – 7.94 (m, 2H), 7.55 – 7.47 (m, 1H), 7.45 – 7.36 (m, 2H), 7.24 – 7.17 (m, 1H), 7.12 (d, J = 5.7 Hz, 2H), 7.05 (d, J = 7.5 Hz, 1H), 4.88 (dd, J = 7.9, 5.1 Hz, 1H), 3.43 – 3.25 (m, 1H), 2.62 – 2.43 (m, 1H), 2.31 (s, 3H). ^{13}C NMR (75 MHz, $CDCl_3$, 300 K) δ (ppm) 196.9, 139.3, 137.5, 136, 133.4, 129.3, 129.0, 128.79, 128.77, 128.7, 126.6 (q, $J_{C\text{-}F}$ = 276.5 Hz), 125.6, 47.3 (q, $J_{C\text{-}F}$ = 2.4 Hz), 37.6 (q, $J_{C\text{-}F}$ = 28.6 Hz), 21.5. ^{19}F {^{1}H} NMR (282 MHz, $CDCl_3$, 300 K) δ (ppm) -64.67. MS (EI) Calcd. for $C_{17}H_{15}F_3O$ M^+: 292.11. Found: 292.1. Spectroscopic data are in accordance with those described in literature.[12]

4,4,4-Trifluoro-1-phenyl-2-(o-tolyl)butan-1-one (3da)

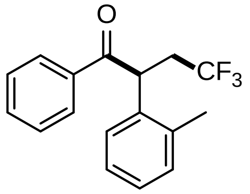

The reaction was performed according to general procedure B with 2-methyl styrene (11.8 mg, 0.1 mmol) and benzoyl fluoride (49.6 mg, 0.4 mmol). After purification by flash chromatography (*n*-pentane/diethyl ether = 40:1), the desired product was obtained as a colorless oil (17.1 mg, 59% yield). ^{1}H NMR (300 MHz, $CDCl_3$, 300 K) δ (ppm) 7.86 – 7.81 (m, 2H), 7.53 – 7.46 (m, 1H), 7.42 – 7.34 (m, 2H), 7.27 – 7.21 (m, 1H), 7.18 – 7.04 (m, 3H), 5.04 (dd, J = 8.5, 4.0 Hz, 1H), 3.51 – 3.29 (m, 1H), 2.56 (s, 3H), 2.44 – 2.25 (m, 1H). ^{13}C NMR (75 MHz, $CDCl_3$, 300 K) δ (ppm) ^{13}C NMR (75 MHz, $CDCl_3$) δ 197.4, 136.4, 136.2, 135.2, 133.3, 131.7, 128.8, 128.6, 128.0, 127.3, 127.1, 126.5 (q, $J_{C\text{-}F}$ = 278.4 Hz) 43.7 (q, $J_{C\text{-}F}$ = 2.5 Hz), 36.8 (q, $J_{C\text{-}F}$ = 28.8 Hz), 19.7. ^{19}F {^{1}H} NMR (282 MHz, $CDCl_3$, 300 K) δ (ppm) -65.13. MS (ESI) Calcd. for $C_{17}H_{15}F_3ONa$ $[M+Na]^+$: 315.0967. Found: 315.0968. Spectroscopic data are in accordance with those described in literature.[12]

4,4,4-Trifluoro-2-(4-methoxyphenyl)-1-phenylbutan-1-one (3ea)

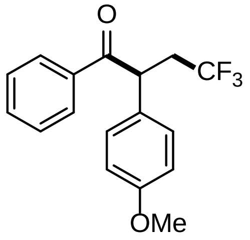

The reaction was performed according to general procedure B with 4-methoxy styrene (13.4 mg, 0.1 mmol) and benzoyl fluoride (49.6 mg, 0.4 mmol). After purification by flash chromatography (*n*-pentane/diethyl ether = 40:1), the desired product was obtained as a colorless oil (23.1 mg, 75% yield). ^{1}H NMR (300 MHz, $CDCl_3$, 300 K) δ (ppm) 8.01 – 7.91 (m, 2H), 7.55 – 7.46 (m, 1H), 7.43 – 7.34 (m, 2H), 7.24 – 7.17 (m, 2H), 6.87 – 6.79 (m, 2H), 4.86 (dd, J = 7.6, 5.6 Hz, 1H), 3.75 (s, 3H), 3.37 – 3.17 (m, 1H), 2.61 – 2.44 (m, 1H). ^{13}C NMR (75 MHz, $CDCl_3$, 300 K) δ (ppm) 197.1, 159.3, 136.0, 133.4, 129.5, 129.3, 128.9, 128.8, 126.6 (q, $J_{C\text{-}F}$ = 276.9 Hz) 114.9, 55.6, 46.5 (q, $J_{C\text{-}F}$ = 2.5 Hz), 37,6 (q, $J_{C\text{-}F}$ = 27.9 Hz). ^{19}F {^{1}H} NMR (282 MHz, $CDCl_3$, 300 K) δ (ppm) -64.52. MS (EI) Calcd. for $C_{17}H_{15}F_3O_2$ M^+: 308.10. Found: 308.1. Spectroscopic data are in accordance with those described in literature.[12]

2-(4-(Chloromethyl)phenyl)-4,4,4-trifluoro-1-phenylbutan-1-one (3fa)

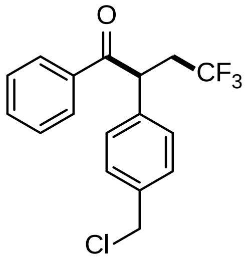

The reaction was performed according to general procedure B with 1-(chloromethyl)-4-vinylbenzene (15.3 mg, 0.1 mmol) and benzoyl fluoride (49.6 mg, 0.4 mmol). After purification by flash chromatography (*n*-pentane/diethyl ether = 40:1), the desired product was obtained as a colorless oil (19.7 mg, 60% yield). ^{1}H NMR (300 MHz, $CDCl_3$, 300 K) δ (ppm) 7.98 – 7.92 (m, 2H), 7.56 – 7.48 (m, 1H), 7.41 (t, J = 7.5 Hz, 2H), 7.37 – 7.28 (m, 4H), 4.92 (dd, J = 7.4, 5.7 Hz, 1H), 4.52 (s, 2H), 3.40 – 3.18 (m, 1H), 2.63 – 2.44 (m, 1H). ^{13}C NMR (75 MHz, $CDCl_3$, 300 K) δ (ppm) 196.7, 137.8, 137.3, 135.8, 133.6, 129.6, 129.0, 128.9, 128.6, 126.5 (q, $J_{C\text{-}F}$ = 277.3 Hz) 47.0 (q, $J_{C\text{-}F}$ = 2.5 Hz), 45.7, 37.5 (q, $J_{C\text{-}F}$ = 28.3 Hz). ^{19}F {^{1}H} NMR (282 MHz, $CDCl_3$, 300 K) δ (ppm) -64.56. HRMS (ESI) Calcd. for $C_{17}H_{14}OClF_3Na$ $[M+Na]^+$: 349.0578. Found: 349.0579. IR (neat): 1683, 1597, 1581, 1514, 1449, 1381, 1343, 1328, 1258, 1216, 1177, 1134, 1098, 1056, 1021, 1002, 973, 938, 878, 806, 784, 746, 688, 586.

4,4,4-Trifluoro-2-(4-fluorophenyl)-1-phenylbutan-1-one (3ga)

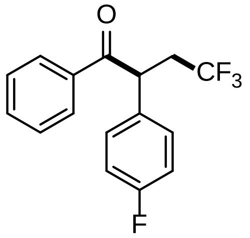

The reaction was performed according to general procedure B with 4-fluoro styrene (12.2 mg, 0.1 mmol) and benzoyl fluoride (49.6 mg, 0.4 mmol). After purification by flash chromatography (*n*-pentane/diethyl ether = 40:1), the desired product was obtained as a colorless oil (12.2 mg, 41% yield). ^{1}H NMR (300 MHz, $CDCl_3$, 300 K) δ (ppm) δ 7.99 – 7.89 (m, 2H), 7.56 – 7.48 (m, 1H), 7.46 – 7.37 (m, 2H), 7.32 – 7.24 (m, 2H), 7.05 – 6.96 (m, 2H), 4.91 (dd, J = 7.4, 5.9 Hz, 1H), 3.36 – 3.16 (m, 1H), 2.54 (dqd, J = 15.1, 10.7, 5.9 Hz, 1H). ^{13}C NMR (75 MHz, $CDCl_3$, 300 K) δ (ppm) 196.8, 162.5 (d, $J_{C\text{-}F}$ = 247.0 Hz), 135.7, 133.6, 133.3 (d, $J_{C\text{-}F}$ = 3.2 Hz), 129.9 (d, $J_{C\text{-}F}$ = 8.0 Hz), 128.93, 128.90, 126.5 (q, $J_{C\text{-}F}$ = 277.3 Hz), 116.5 (d, $J_{C\text{-}F}$ = 21.6 Hz), 46.5 (q, $J_{C\text{-}F}$ = 2.1 Hz), 37.6 (q, $J_{C\text{-}F}$ = 29.0 Hz). ^{19}F {^{1}H} NMR (282 MHz, $CDCl_3$, 300 K) δ (ppm) -64.48, -114.07. MS (EI) Calcd. for $C_{16}H_{12}F_4O$ M^+: 296.08. Found: 296.1. Spectroscopic data are in accordance with those described in literature.[12]

2-(4-Chlorophenyl)-4,4,4-trifluoro-1-phenylbutan-1-one (3ha)

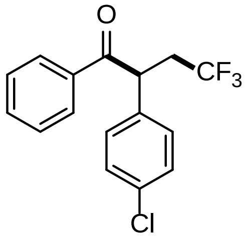

The reaction was performed according to general procedure B with 4-chloro styrene (13.9 mg, 0.1 mmol) and benzoyl fluoride (49.6 mg, 0.4 mmol). After purification by flash chromatography (*n*-pentane/diethyl ether = 40:1), the desired product was obtained as a colorless oil (16.7 mg, 53% yield). ^{1}H NMR (300 MHz, $CDCl_3$, 300 K) δ (ppm) 7.87 – 7.78 (m, 2H), 7.47 – 7.37 (m, 1H), 7.35 – 7.27 (m, 2H), 7.21 – 7.11 (m, 4H), 4.78 (dd J = 7.3, 5.9 Hz, 1H), 3.15 (dqd, J = 15.1, 10.7, 7.3 Hz, 1H), 2.43 (dqd, J = 15.1, 10.6, 5.9 Hz, 1H). ^{13}C NMR (75 MHz, $CDCl_3$, 300 K) δ (ppm) 196.6, 136.0, 135.7, 134.1, 133.7, 129.7, 129.6, 128.9, 46.7 (q, $J_{C\text{-}F}$ = 2.5 Hz), 37.5 (q, $J_{C\text{-}F}$ = 28.6 Hz). (CF_3 signal intensity too low) ^{19}F {^{1}H} NMR (282 MHz, $CDCl_3$, 300 K) δ (ppm) -64.46. MS (ESI) Calcd. for $C_{16}H_{12}OClF_3Na$ $[M+Na]^+$: 335.0421. Found: 335.0423. Spectroscopic data are in accordance with those described in literature.[12]

4,4,4-Trifluoro-1-phenyl-2-(4-(trifluoromethyl)phenyl)butan-1-one (3ia)

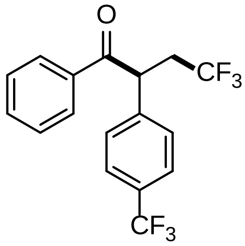

The reaction was performed according to general procedure B with 4-trifluoromethyl styrene (17.2 mg, 0.1 mmol) and benzoyl fluoride (49.6 mg, 0.4 mmol). After purification by flash chromatography (*n*-pentane/diethyl ether = 40:1), the desired product was obtained as a colorless oil (14.5 mg, 42% yield). ^{1}H NMR (300 MHz, $CDCl_3$, 300 K) δ (ppm) 7.90 – 7.82 (m, 2H), 7.54 – 7.43 (m, 3H), 7.35 (td, J = 8.3, 1.5 Hz, 4H), 4.90 (t, J = 6.6 Hz, 1H), 3.21 (dqd, J = 15.1, 10.7, 7.3 Hz, 1H), 2.49 (dqd, J = 15.0, 10.6, 6.0 Hz, 1H). ^{13}C {^{19}F} NMR (150 MHz, $CDCl_3$, 300 K) δ (ppm) 196.3, 141.4, 135.5, 133.9, 130.4, 129.0, 128.9, 128.7, 126.5, 126.3, 125.4, 124.0, 47.0, 37.4. ^{19}F {^{1}H} NMR (282 MHz, $CDCl_3$, 300 K) δ (ppm) -62.79, -64.49. MS (EI) Calcd. For $C_{17}H_{12}OF_5$ $[M\text{-}F]^+$: 327.0803. Found: 327.0802. Spectroscopic data are in accordance with those described in literature.[12]

2-([1,1'-Biphenyl]-4-yl)-4,4,4-trifluoro-1-phenylbutan-1-one (3ja)

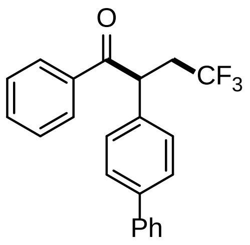

The reaction was performed according to general procedure B with 4-vinyl-1,1'-biphenyl (18.0 mg, 0.1 mmol) and benzoyl fluoride (49.6 mg, 0.4 mmol). After purification by flash chromatography (*n*-pentane/diethyl ether = 40:1), the desired product was obtained as a colorless oil (25.7 mg, 73% yield). ^{1}H NMR (300 MHz, $CDCl_3$, 300 K) δ (ppm) 8.06 – 7.95 (m, 2H), 7.58 – 7.49 (m, 5H), 7.47 – 7.31 (m, 7H), 4.96 (dd, J = 7.8, 5.4 Hz, 1H), 3.35 (dqd, J = 15.0, 10.8, 7.7 Hz, 1H), 2.70 – 2.49 (m, 1H). ^{13}C NMR (75 MHz, $CDCl_3$, 300 K) δ (ppm) 196.9, 141.0, 140.4, 136.5, 136.0, 133.6, 129.0, 128.9, 128.9, 128.6, 128.2, 127.7, 127.2, 126.6 (q, $J_{C\text{-}F}$ = 277.9 Hz) 47.0 (q, $J_{C\text{-}F}$ = 2.6 Hz),37.6 (q, $J_{C\text{-}F}$ = 28.3 Hz). ^{19}F {^{1}H} NMR (282 MHz, $CDCl_3$, 300 K) δ (ppm) -64.55. MS (EI) Calcd. for $C_{22}H_{17}F_3O$ M^+: 354.12. Found: 354.1. Spectroscopic data are in accordance with those described in literature.[12]

4,4,4-Trifluoro-2-(naphthalen-2-yl)-1-phenylbutan-1-one (3ka)

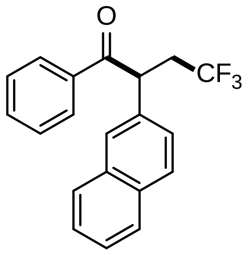

The reaction was performed according to general procedure B with 2-vinyl naphthalene (15.4 mg, 0.1 mmol) and benzoyl fluoride (49.6 mg, 0.4 mmol). After purification by flash chromatography (*n*-pentane/diethyl ether = 40:1), the desired product was obtained as a colorless oil (17.1 mg, 52% yield). ^{1}H NMR (300 MHz, $CDCl_3$, 300 K) δ (ppm) 8.06 – 7.97 (m, 2H), 7.86 – 7.73 (m, 4H), 7.52 – 7.34 (m, 6H), 5.08 (dd, J = 7.6, 5.5 Hz, 1H), 3.53 – 3.30 (m, 1H), 2.75 – 2.54 (m, 1H). ^{13}C NMR (75 MHz, $CDCl_3$, 300 K) δ (ppm) 196.8, 135.9, 135.0, 133.8, 133.5, 132.9, 129.5, 129.0, 128.8, 128.0, 127.8, 127.4, 126.7, 126.5, 125.7, 47.51 (q, $J_{C\text{-}F}$ = 2.6 Hz), 37.6 (q, $J_{C\text{-}F}$ = 28.3 Hz). (CF_3 signal intensity to low) ^{19}F {^{1}H} NMR (282 MHz, $CDCl_3$, 300 K) δ (ppm) -64.50. MS (EI) Calcd. for $C_{20}H_{15}F_3O$ M^+: 328.11. Found: 328.1. Spectroscopic data are in accordance with those described in literature.[12]

4,4,4-Trifluoro-1-phenyl-2-(pyridin-2-yl)butan-1-one (3la)

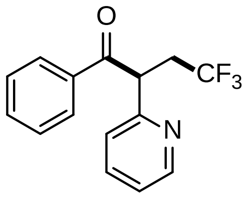

The reaction was performed according to general procedure B with 2-vinyl pyridine (10.5 mg, 0.1 mmol) and benzoyl fluoride (49.6 mg, 0.4 mmol). After purification by flash chromatography (*n*-pentane/diethyl ether/triethyl amine = 20:1:0.1), the desired product was obtained as a yellowish oil (14.9 mg, 53% yield). ^{1}H NMR (300 MHz, $CDCl_3$, 300 K) δ (ppm) 8.58 – 8.54 (m, 1H), 8.04 (dd, *J* = 7.1, 1.6 Hz, 1H), 7.62 (td, *J* = 7.7, 1.9 Hz, 1H), 7.56 – 7.48 (m, 1H), 7.45 – 7.37 (m, 1H), 7.32 – 7.27 (m, 1H), 7.15 (ddd, *J* = 7.6, 4.9, 1.2 Hz, 1H), 5.17 (t, *J* = 6.7 Hz, 1H), 3.37 – 3.18 (m, 1H), 2.78 (dqd, *J* = 15.1, 10.7, 6.3 Hz, 1H). ^{13}C NMR (75 MHz, $CDCl_3$, 300 K) δ (ppm) 196.1, 157.2, 150.3, 137.3, 135.8, 133.6, 129.2, 128.8, 126.7 (q, J_{C-F} = 276.9 Hz), 122.9, 122.7, 50.1 (q, J_{C-F} = 2.3 Hz), 36.2 (q, J_{C-F} = 28.7 Hz). ^{19}F {^{1}H} NMR (282 MHz, $CDCl_3$, 300 K) δ (ppm) -64.45. MS (EI) Calcd. for $C_{15}H_{12}F_3NO$ M^+: 279.09. Found: 279.1. Spectroscopic data are in accordance with those described in literature.[13]

2-Benzyl-4,4,4-trifluoro-1-phenylbutan-1-one (3ma)

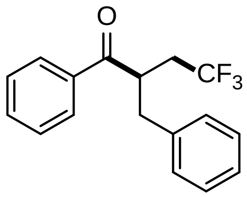

The reaction was performed according to general procedure B with allyl benzene (11.8 mg, 0.1 mmol) and benzoyl fluoride (49.6 mg, 0.4 mmol). After purification by flash chromatography (*n*-pentane/diethyl ether = 40:1), the desired product was obtained as a colorless oil (4.4 mg, 15% yield). ^{1}H NMR (300 MHz, $CDCl_3$, 300 K) δ (ppm) 7.88 – 7.74 (m, 2H), 7.53 – 7.44 (m, 1H), 7.42 – 7.30 (m, 2H), 7.22 – 7.04 (m, 5H), 4.05 – 3.88 (m, 1H), 3.02 (dd, *J* = 13.8, 6.5 Hz, 1H), 2.88 – 2.72 (m, 1H), 2.68 (dd, *J* = 13.9, 7.9 Hz, 1H), 2.16 (dqd, *J* = 14.6, 11.0, 3.5 Hz, 1H). ^{13}C NMR (75 MHz, $CDCl_3$, 300 K) δ (ppm) 200.9, 137.6, 136.4, 133.6, 129.2, 128.9, 128.9, 128.5, 127.1, 126.7 (q, J_{C-F} = 277.0 Hz) 41.9 (q, J_{C-F} = 2.6 Hz), 39.0, 34.8 (q, J_{C-F} = 28.3 Hz). ^{19}F {^{1}H} NMR (282 MHz, $CDCl_3$, 300 K) δ (ppm) -64.58. HRMS (EI) Calcd. for $C_{17}H_{15}OF_3$ M^+: 292.1070. Found: 292.1073. IR (neat): 1679, 1600, 1498, 1448, 1390, 1359, 1315, 1293, 1267, 1251, 1224, 1178, 1138, 1104, 1085, 1006, 976, 942, 915, 887, 826, 787, 752, 741, 698, 685, 657, 556.

1-phenyl-2-(2,2,2-trifluoroethyl)octan-1-one (3na)

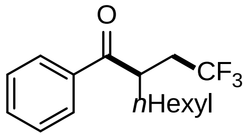

The reaction was performed according to general procedure B with 1-octene (11.2 mg, 0.1 mmol) and benzoyl fluoride (49.6 mg, 0.4 mmol). After purification by flash chromatography (*n*-pentane/diethyl ether = 40:1), the desired product was obtained as a light yellow oil (5.7 mg, 20% yield). ^{1}H NMR (500 MHz, $CDCl_3$, 300 K) δ (ppm) 7.98 – 7.92 (m, 2H), 7.62 – 7.57 (m, 1H), 7.52 – 7.47 (m, 2H), 3.79 – 3.72 (m, 1H), 2.95 – 2.82 (m, 1H), 2.30 – 2.19 (m, 1H), 1.79 – 1.70 (m, 1H), 1.58 – 1.50 (m, 1H, overlapped with water signal), 1.28 – 1.21 (m, 8H), 0.84 (t, J = 7.0 Hz, 3H). ^{13}C {^{19}F} NMR (126 MHz, $CDCl_3$, 300 K) δ (ppm) 202.1, 136.1, 134.5, 129.0, 128.4, 126.8, 39.8, 35.1, 32.7, 31.6, 29.2, 26.8, 22.6, 14.5. ^{19}F {^{1}H} NMR (470 MHz, $CDCl_3$, 300 K) δ (ppm) -64.85. MS (EI) Calcd. for $C_{16}H_{21}F_3O$ $[M]^+$: 286.15. Found: 286.12. IR (neat): 1686, 1598, 1582, 1449, 1377, 1326, 1258, 1223, 1149, 1119, 1095, 1068, 1027, 983, 836, 789, 707, 687, 657, 574.

***trans*-Phenyl(2-(trifluoromethyl)-2,3-dihydro-1H-inden-1-yl)methanone (*trans*-3oa)**

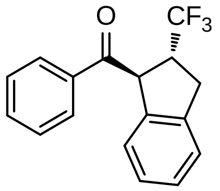

The reaction was performed according to general procedure B with indene (11.6 mg, 0.1 mmol) and benzoyl fluoride (49.6 mg, 0.4 mmol). After purification by flash chromatography (*n*-pentane/diethyl ether = 40:1), the desired product was obtained as a colorless oil (16.1 mg, 55% yield). ^{1}H NMR (300 MHz, $CDCl_3$, 300 K) δ (ppm) 8.18 – 8.05 (m, 2H), 7.73 – 7.64 (m, 1H), 7.63 – 7.53 (m, 2H), 7.31 – 7.18 (m, 2H), 7.08 (t, J = 7.4 Hz, 1H), 6.90 (d, J = 7.7 Hz, 1H), 5.31 (d, J = 6.8 Hz, 1H), 4.13 – 3.90 (m, 1H), 3.44 (dd, J = 16.5, 9.3 Hz, 1H), 3.25 (dd, J = 16.5, 7.6 Hz, 1H). ^{13}C NMR (75 MHz, $CDCl_3$, 300 K) δ (ppm) 197.5, 141.3, 139.5, 136.9, 134.0, 129.4, 129.2, 128.3, 127.9 (q, $J_{C\text{-}F}$ = 276.2 Hz), 127.3, 125.1, 124.4, 52.6 (q, $J_{C\text{-}F}$ = 2.2 Hz), 45.0 (q, $J_{C\text{-}F}$ = 27.6 Hz), 32.4 (q, $J_{C\text{-}F}$ = 2.6 Hz). ^{19}F {^{1}H} NMR (282 MHz, $CDCl_3$, 300 K) δ (ppm) -70.76. HRMS (EI) Calcd. for $C_{17}H_{13}OF_3$ M^+: 290.0913. Found: 290.0916. IR (neat): 1686, 1597, 1581, 1480, 1448, 1399, 1323, 1268, 1219, 1202, 1174, 1151, 1104, 1063, 1029, 1002, 972, 950, 927, 885, 836, 764, 746, 693, 660, 622, 602, 568.

***trans*-Phenyl(2-(trifluoromethyl)-2,3-dihydrobenzofuran-3-yl)methanone (*trans*-3pa)**

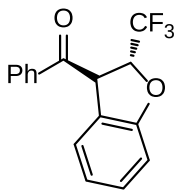

The reaction was performed according to general procedure B with benzofuran (11.8 g, 0.1 mmol) and benzoyl fluoride (49.6 mg, 0.4 mmol). After purification by flash chromatography (*n*-pentane/ethyl acetate = 50:1), the desired product was obtained as a colorless oil (10.5 mg, 36% yield). ^{1}H NMR (600 MHz, $CDCl_3$) δ (ppm) 8.11 (dd, *J* = 8.4, 1.3 Hz, 2H), 7.75 – 7.68 (m, 1H), 7.64 – 7.53 (m, 2H), 7.20 (dddd, *J* = 8.2, 7.4, 1.4, 0.8 Hz, 1H), 6.99 – 6.90 (m, 2H), 6.81 (td, *J* = 7.6, 1.0 Hz, 1H), 5.78 (qd, *J* = 6.9, 5.5 Hz, 1H), 5.41 – 5.34 (m, 1H). ^{13}C NMR (150 MHz, $CDCl_3$) δ (ppm) 193.5, 158.9, 135.4, 134.5, 130.1, 129.6, 129.3, 124.8, 124.2 (q, $J_{C\text{-}F}$ = 278.7 Hz), 123.0, 121.8, 110.8, 80.0 (q, $J_{C\text{-}F}$ = 33.1 Hz), 50.3 (d, $J_{C\text{-}F}$ = 1.4 Hz). ^{13}C NMR {^{19}F} (150 MHz, $CDCl_3$) δ 193.5, 158.9, 135.4, 134.5, 130.1, 129.6, 129.3, 124.8, 123.0, 121.8, 110.8, 80.0, 50.3. ^{19}F NMR (282 MHz, $CDCl_3$) δ (ppm) -78.82, -78.84. HRMS (ESI) Calcd. for $C_{16}H_{11}F_3NaO_2$ $[M+Na]^+$: 315.0609. Found: 315.0605. IR (neat): 1689, 1596, 1479, 1463, 1450, 1392, 1341, 1300, 1273, 1230, 1172, 1133, 1103, 1053, 1026, 932, 896, 878, 853, 816, 750, 693, 636, 615.

7,7,7-trifluoro-1,2-diphenylhept-4-en-1-one (*E*,*Z*-isomer, 7)

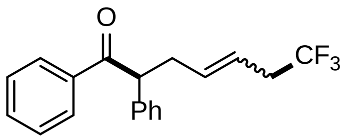

The reaction was performed according to general procedure B with (2-vinylcyclopropyl)benzene (14.4 g, 0.1 mmol) and benzoyl fluoride (49.6 mg, 0.4 mmol). After purification by flash chromatography (*n*-pentane/ethyl acetate = 50:1), the desired product was obtained as a colorless oil (35.1 mg, 55% yield). Due to the overlap for *E* and *Z* isomers in ^{1}H NMR, we used ^{13}C NMR to confirm its ratio. ^{1}H NMR (*E*,*Z*-isomer, 300 MHz, $CDCl_3$) δ (ppm) 8.01 – 7.89 (m, 2H), 7.53 – 7.44 (m, 1H), 7.38 (tt, *J* = 6.8, 1.6 Hz, 2H), 7.34 – 7.16 (m, 5H), 5.65 (dt, *J* = 14.6, 7.0 Hz, 1H), 5.41 (dtt, *J* = 15.3, 7.0, 1.3 Hz, 1H), 4.58 (td, *J* = 7.3, 4.2 Hz, 1H), 3.02 – 2.87 (m, 1H), 2.87 – 2.49 (m, 3H). ^{13}C NMR (*E*-isomer, 75 MHz, $CDCl_3$) δ (ppm) 199.1, 138.9, 136.8, 135.4, 133.1, 129.1, 128.8, 128.7, 128.4, 127.4, 126.0 (q, $J_{C\text{-}F}$ = 274.8 Hz),, 120.3 (q, $J_{C\text{-}F}$ = 3.6 Hz), 53.9, 37.4 (q, $J_{C\text{-}F}$ = 29.4 Hz), 37.0. ^{19}F {^{1}H} NMR (*E*,*Z*-isomer, 282 MHz, $CDCl_3$) δ (ppm) -66.10, -66.59. HRMS (EI) Calcd. for $C_{19}H_{17}F_3O$ M^+: 318.1231. Found: 318.1225. IR (*E*,*Z*-isomer, neat): 1681, 1598, 1581, 1494, 1448, 1430, 1348, 1249, 1204, 1176, 1132, 1056, 1030, 1002, 971, 944, 913, 844, 756, 697, 648, 595.

6. ^{1}H, ^{13}C and ^{19}F NMR spectra

4,4,4-trifluoro-1,2-diphenylbutan-1-one (3aa)

^{1}H NMR (300 MHz, $CDCl_3$, 300 K)

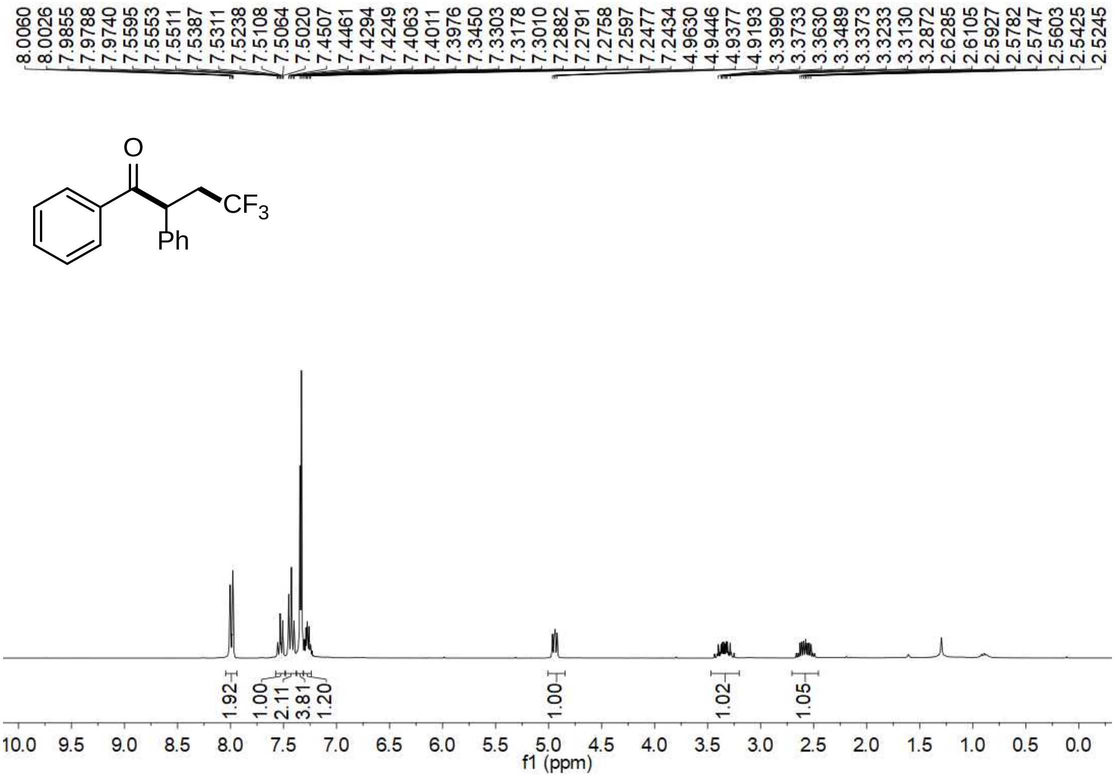

^{13}C NMR (75 MHz, $CDCl_3$, 300 K)

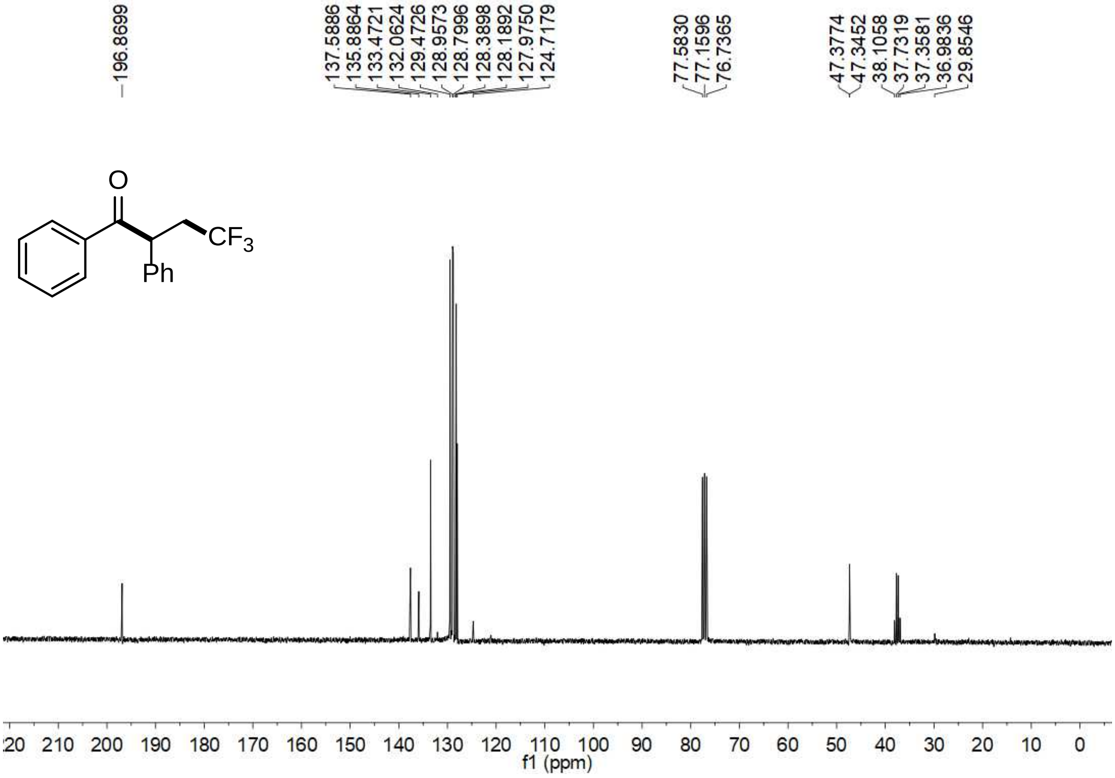

^{19}F {^{1}H} NMR (282 MHz, $CDCl_3$, 300 K)

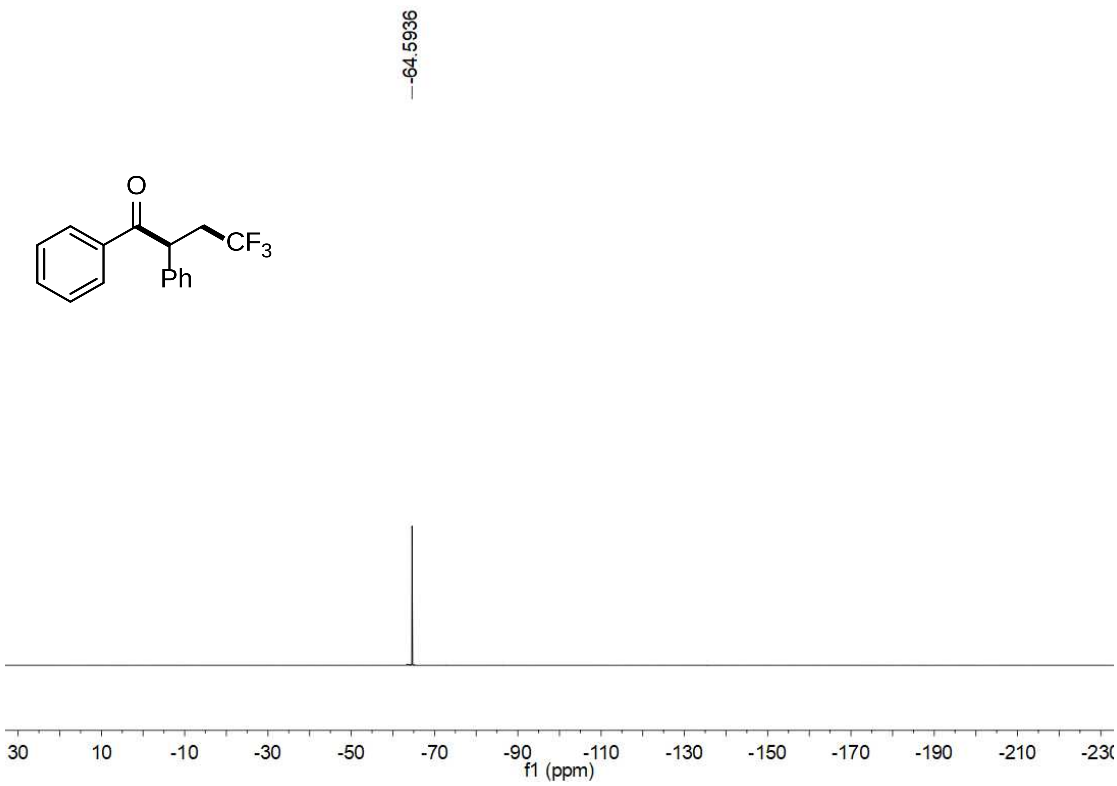

4,4,4-trifluoro-1-(4-methoxyphenyl)-2-phenylbutan-1-one (3ab)

^{1}H NMR (300 MHz, $CDCl_3$, 300 K)

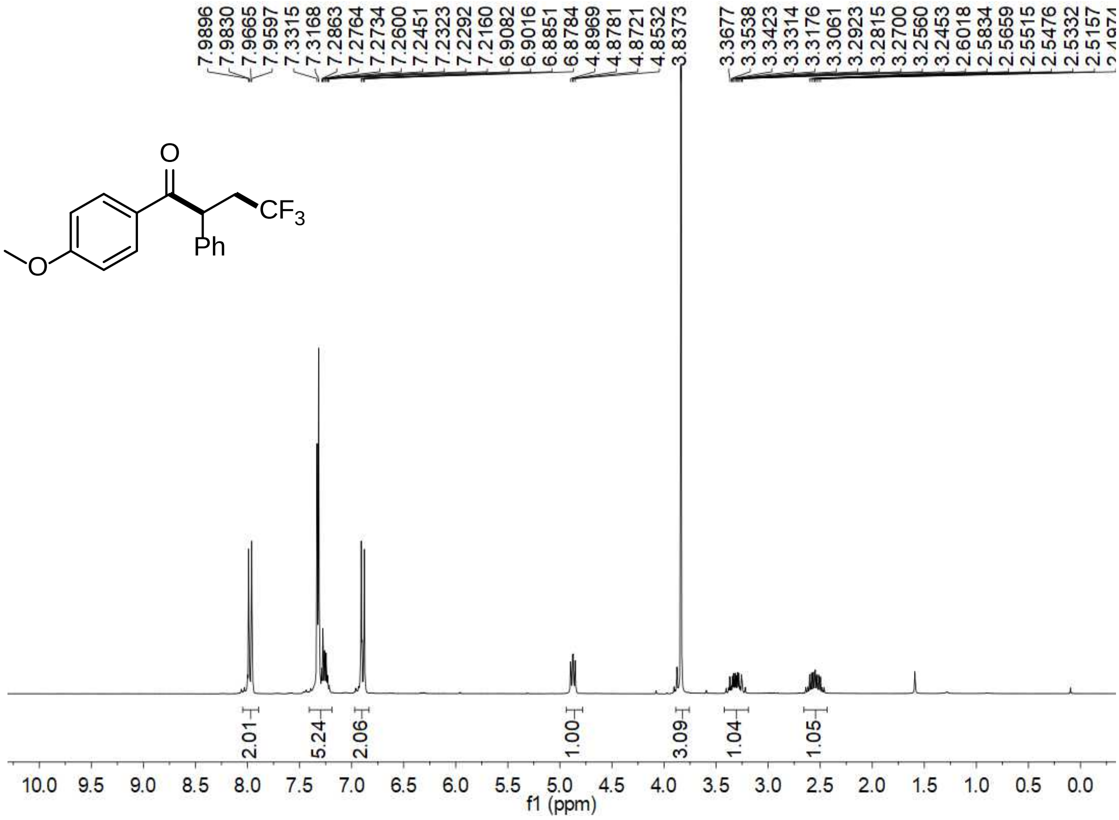

^{13}C NMR (75 MHz, $CDCl_3$, 300 K)

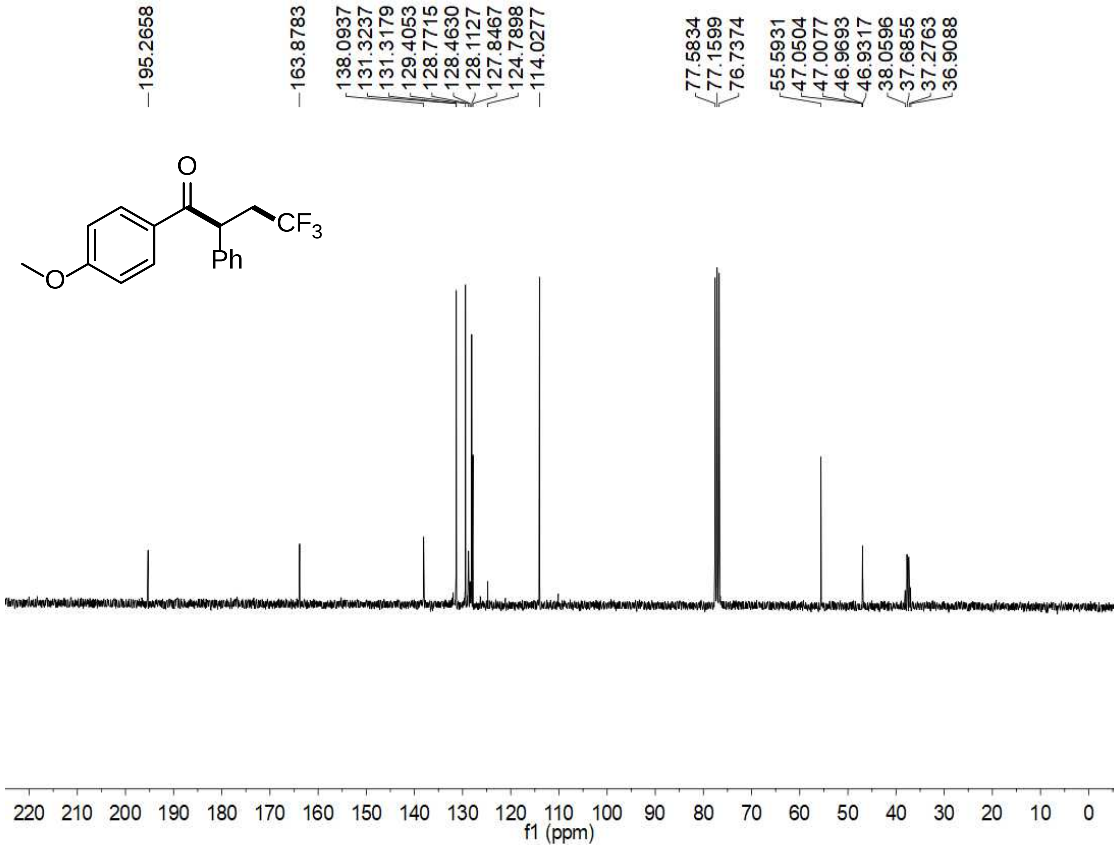

^{19}F {^{1}H} NMR (282 MHz, $CDCl_3$, 300 K)

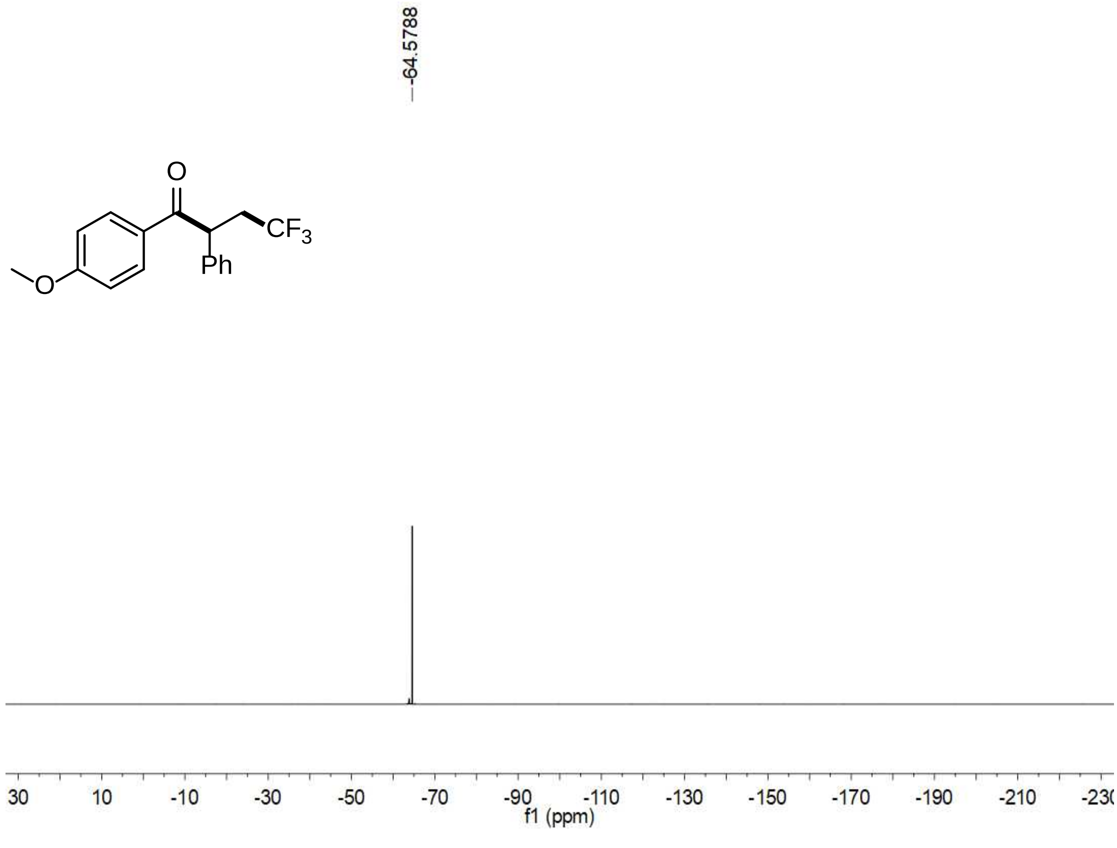

4,4,4-trifluoro-1-(4-fluorophenyl)-2-phenylbutan-1-one (3ac)

^{1}H NMR (300 MHz, $CDCl_3$, 300 K)

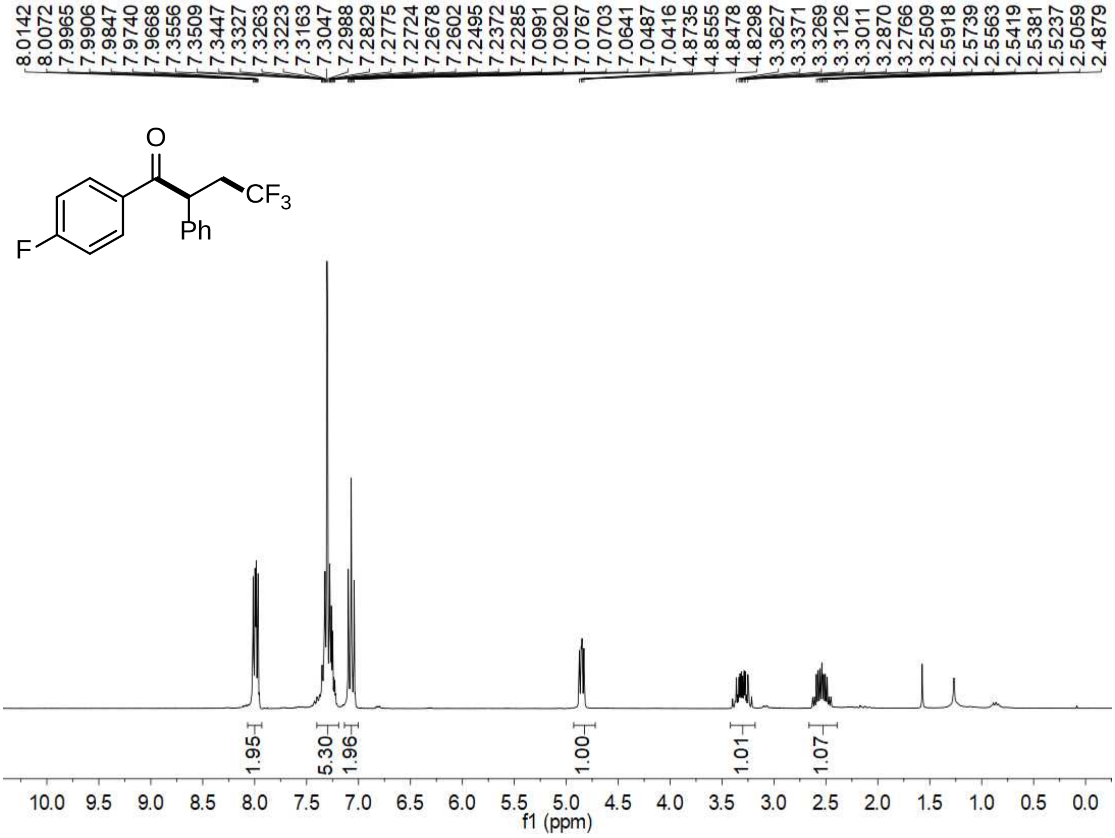

^{13}C NMR (75 MHz, $CDCl_3$, 300 K)

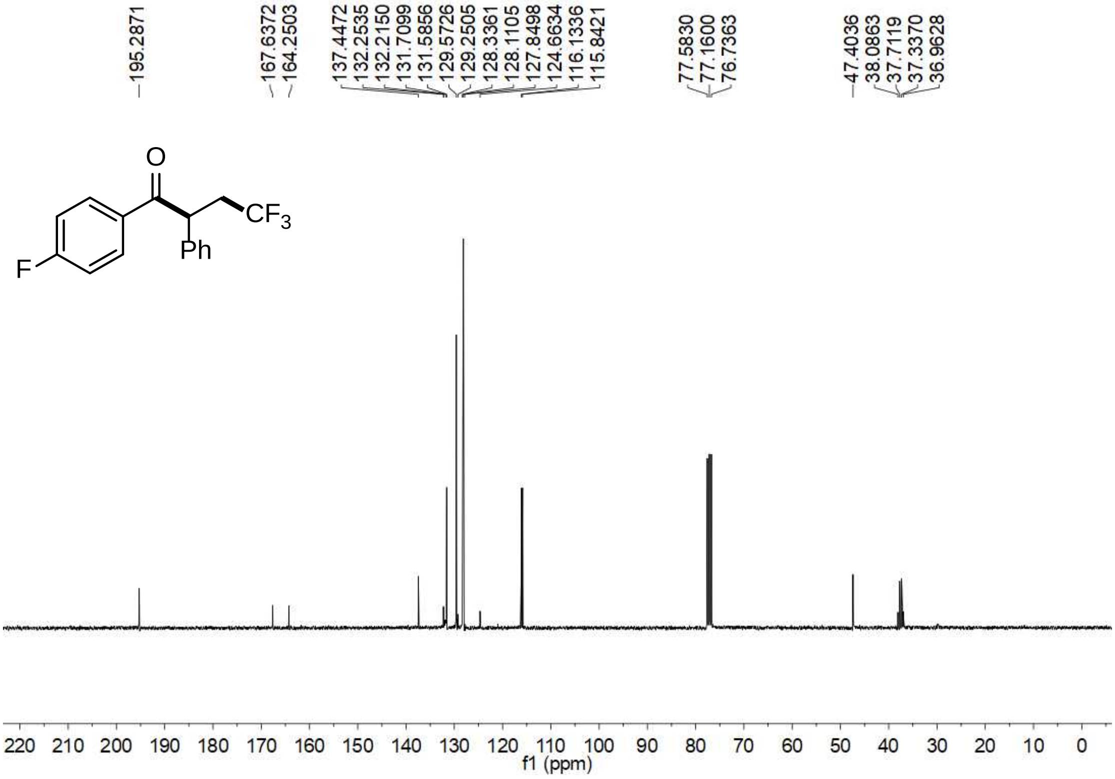

$^{19}F\{^1H\}$ NMR (282 MHz, $CDCl_3$, 300 K)

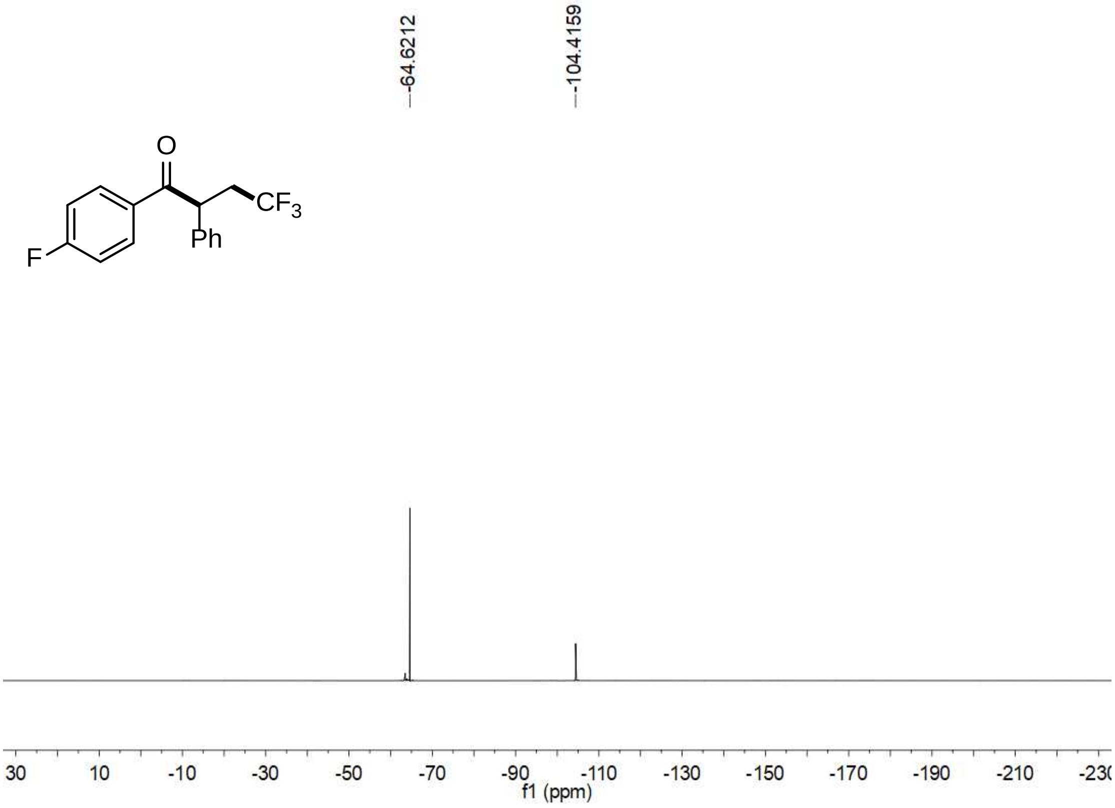

1-(4-chlorophenyl)-4,4,4-trifluoro-2-phenylbutan-1-one (3ad)

^1H NMR (300 MHz, $CDCl_3$, 300 K)

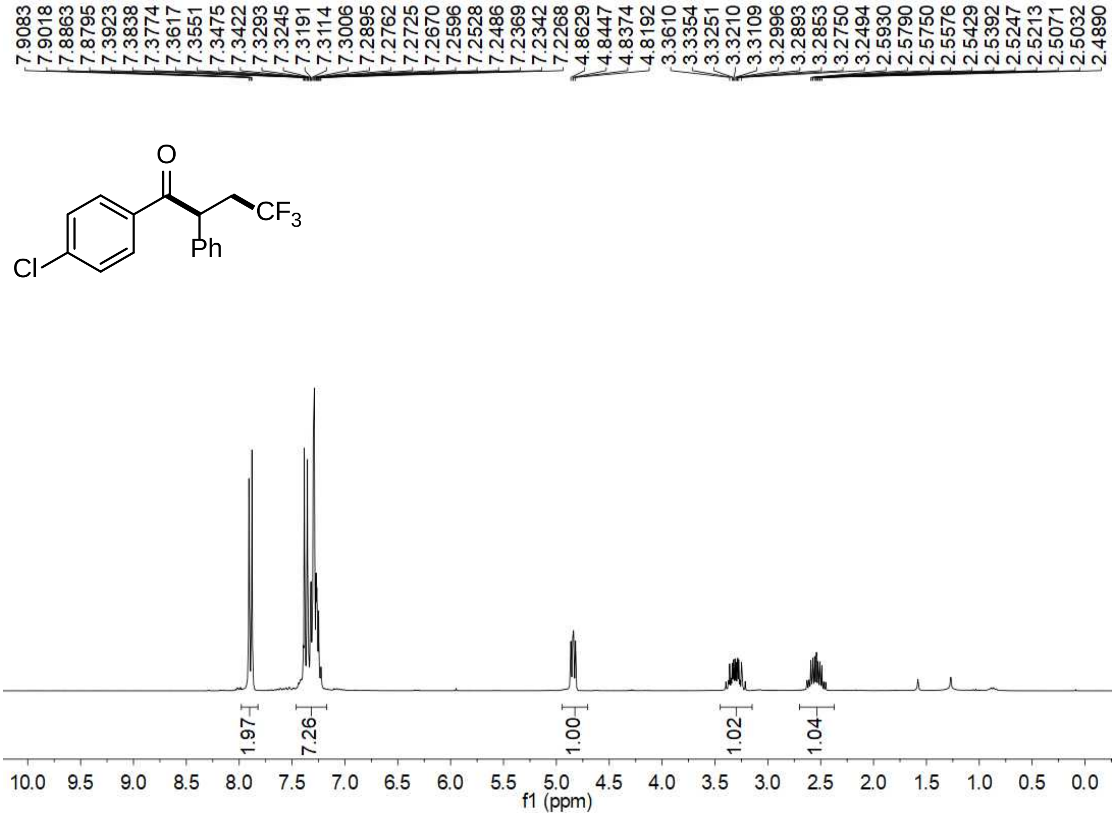

^{13}C NMR (75 MHz, $CDCl_3$, 300 K)

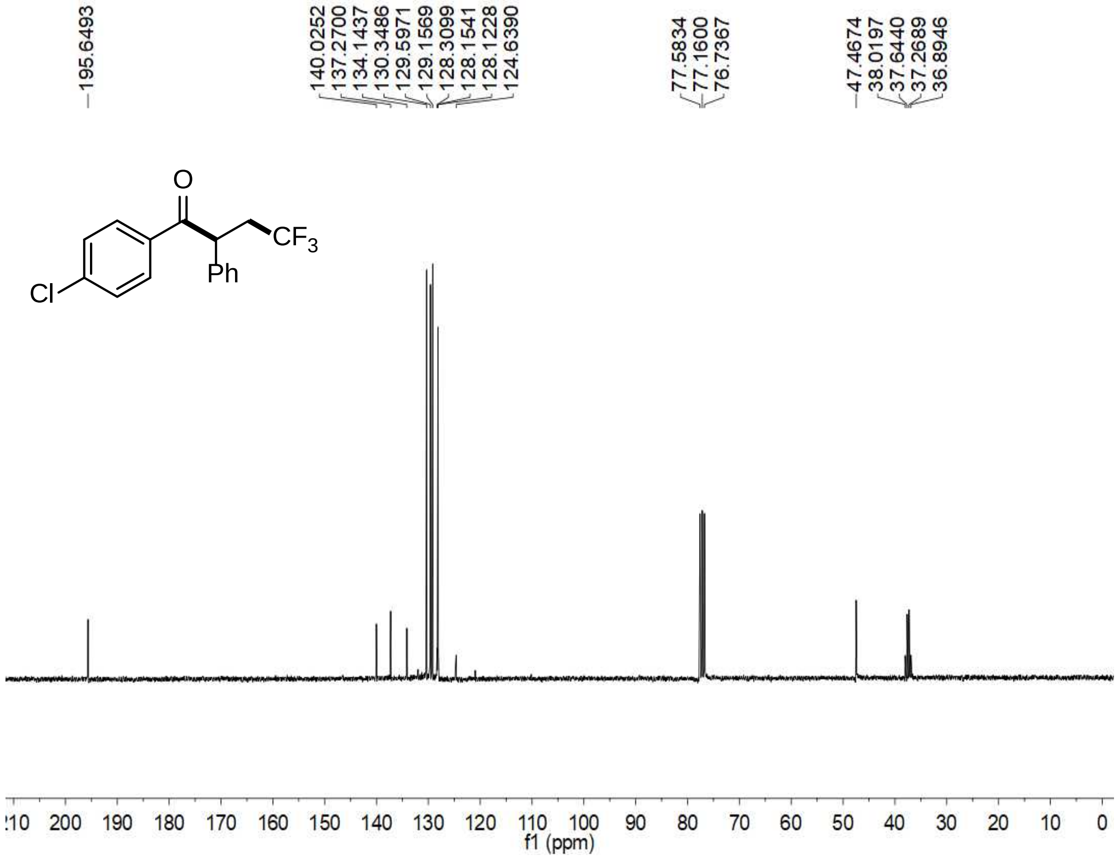

^{19}F {^{1}H} NMR (282 MHz, $CDCl_3$, 300 K)

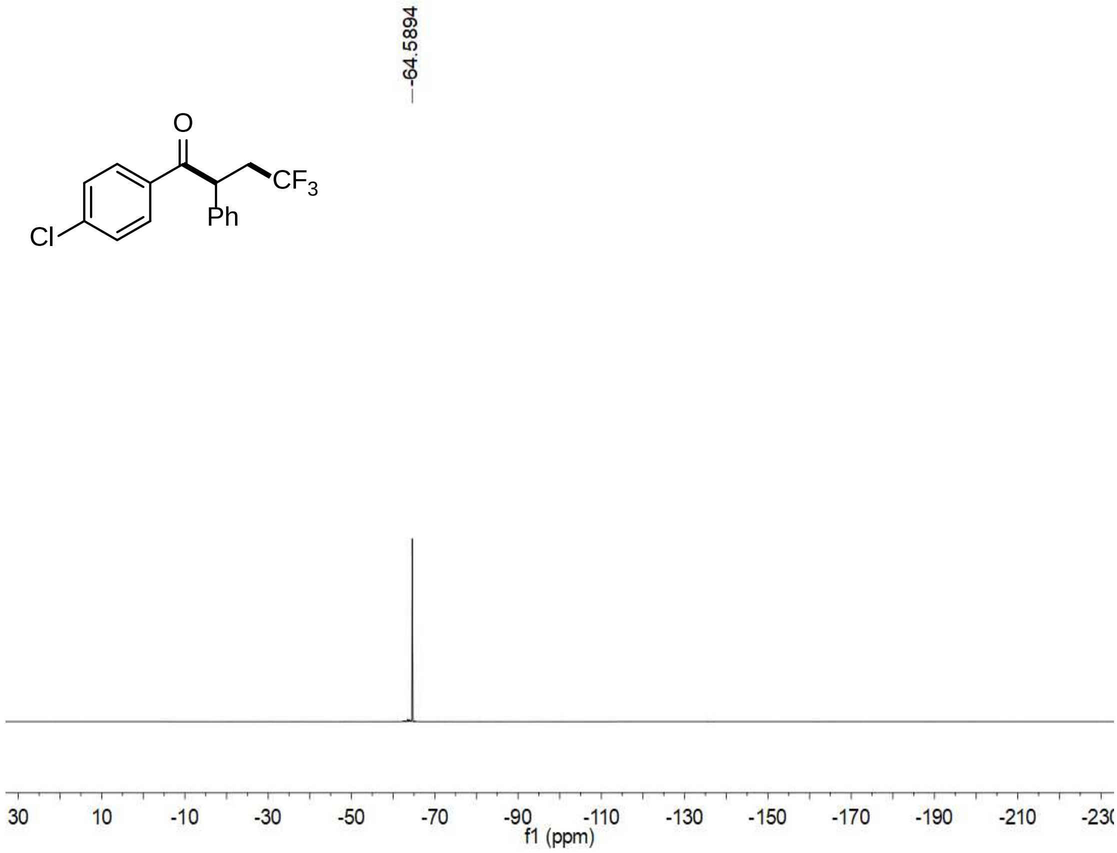

1-(4-bromophenyl)-4,4,4-trifluoro-2-phenylbutan-1-one (3ae)

^{1}H NMR (300 MHz, $CDCl_3$)

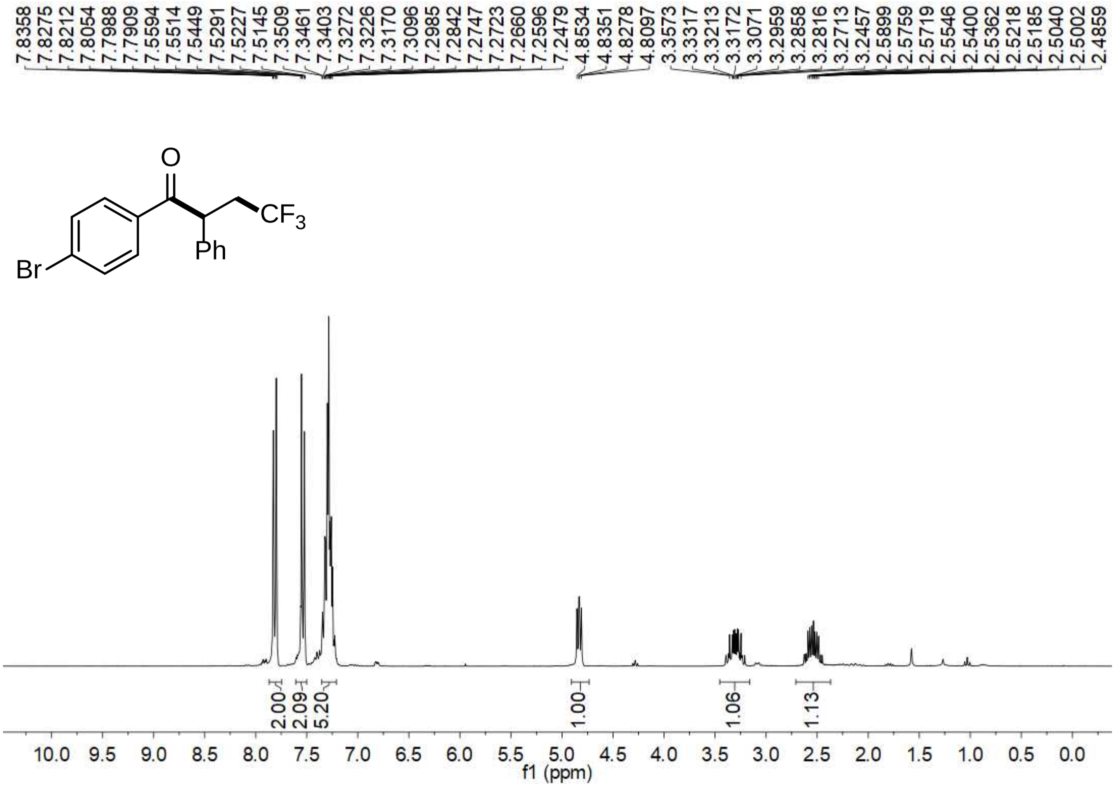

^{13}C NMR (75 MHz, $CDCl_3$)

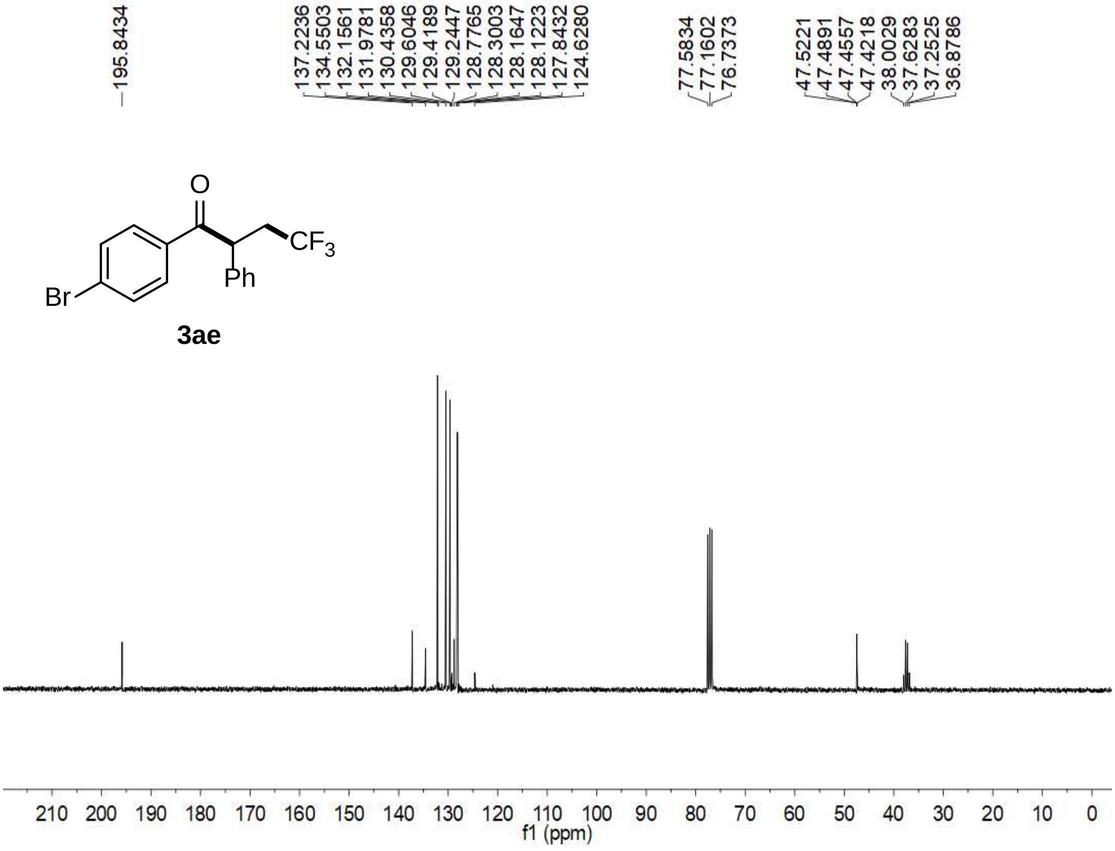

$^{19}F\ \{^{1}H\}$ NMR (282 MHz, $CDCl_3$)

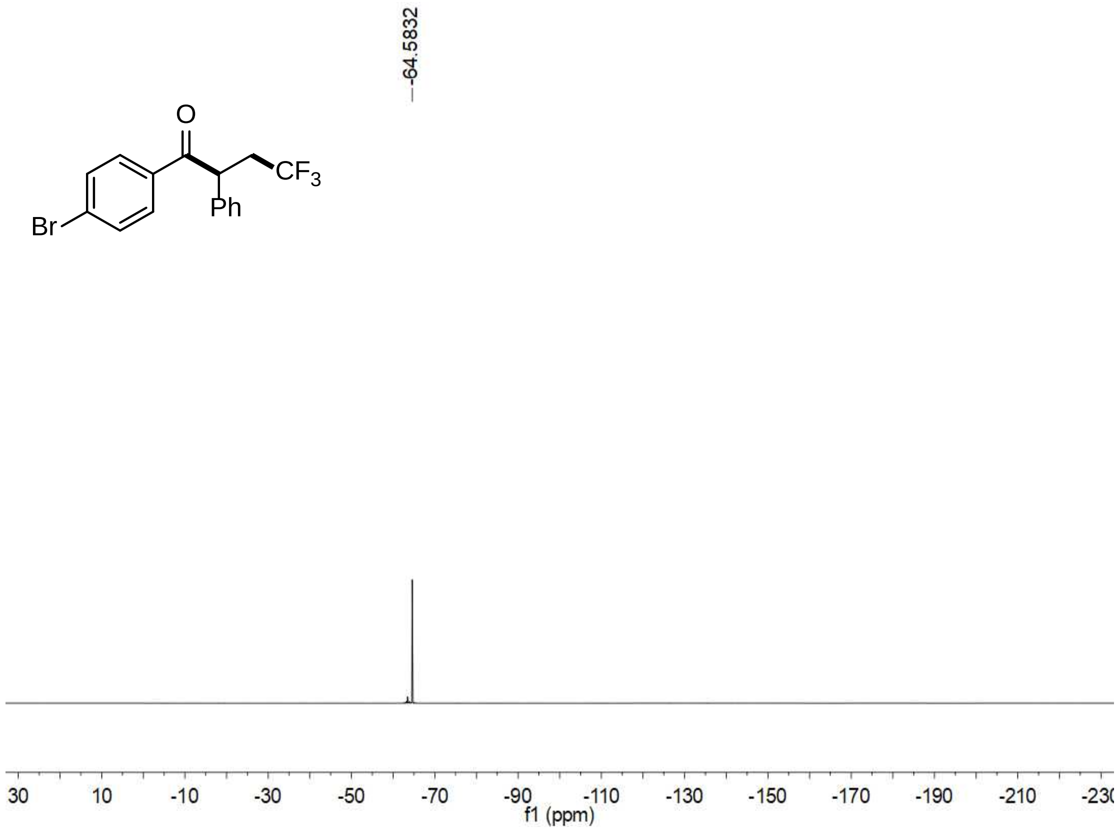

4,4,4-trifluoro-1-(4-iodophenyl)-2-phenylbutan-1-one (3af)

^{1}H NMR (300 MHz, $CDCl_3$)

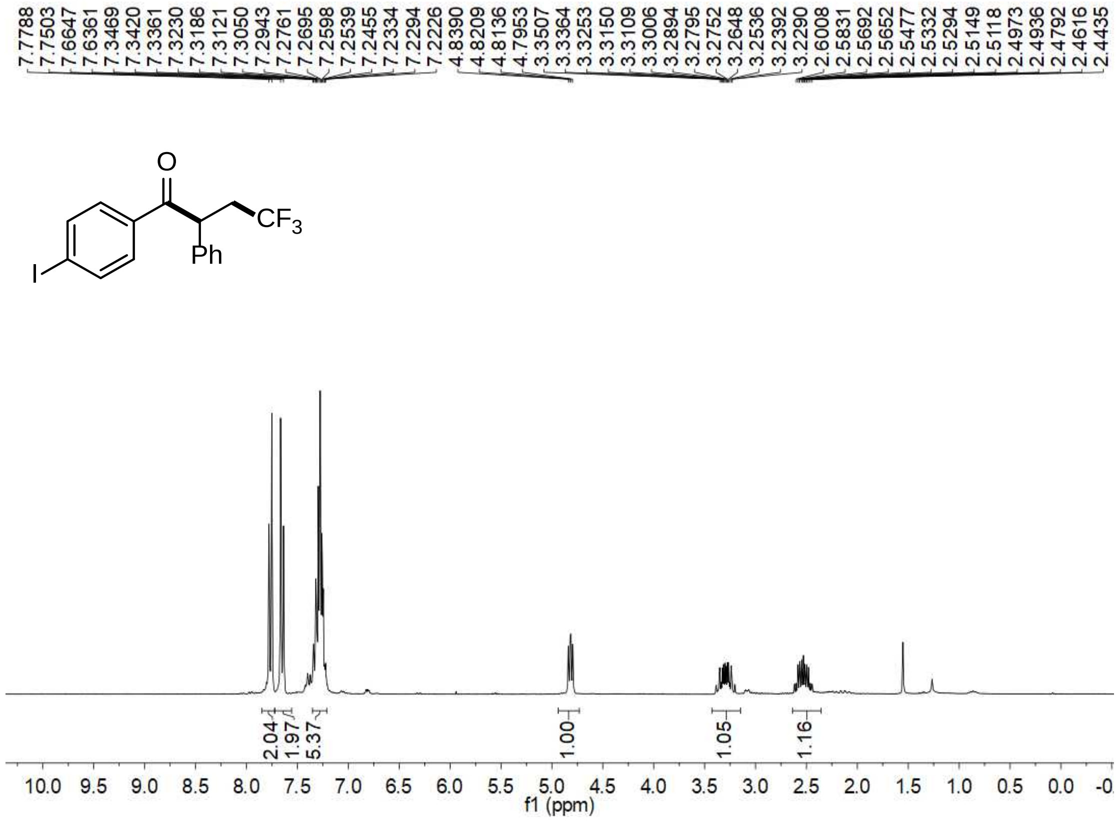

^{13}C NMR (75 MHz, $CDCl_3$)

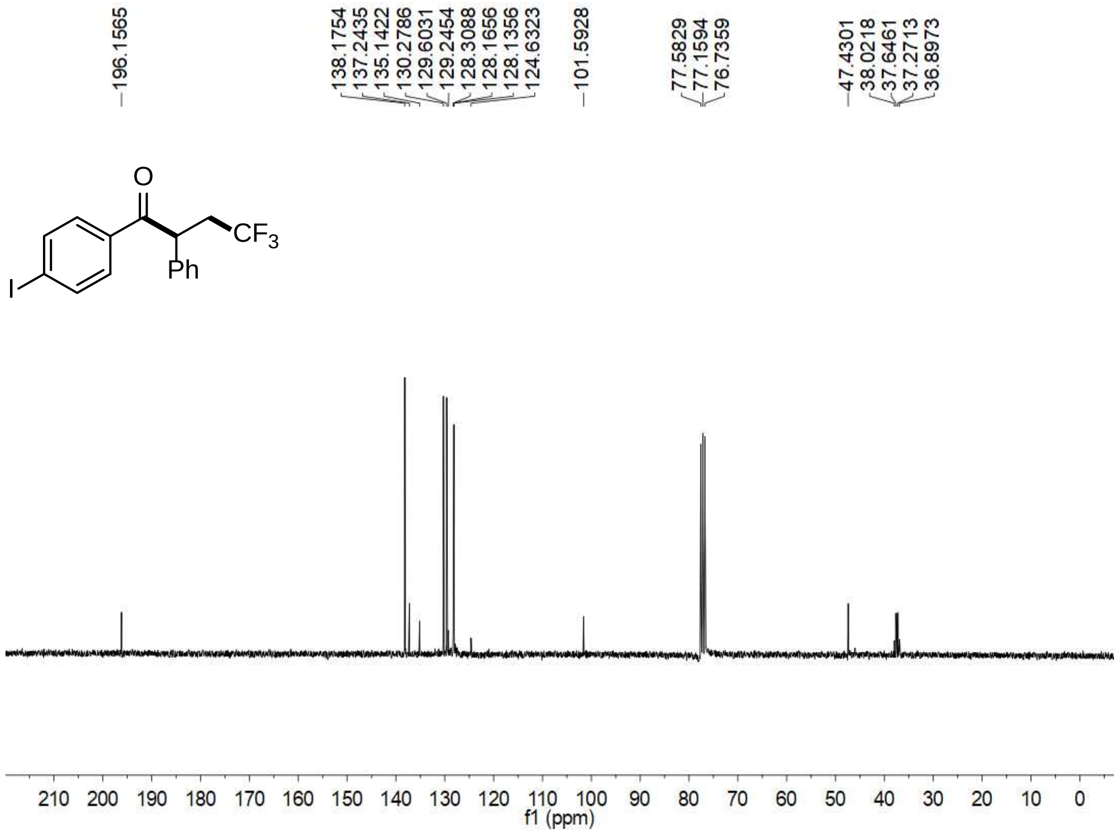

^{19}F {^{1}H} NMR (282 MHz, $CDCl_3$)

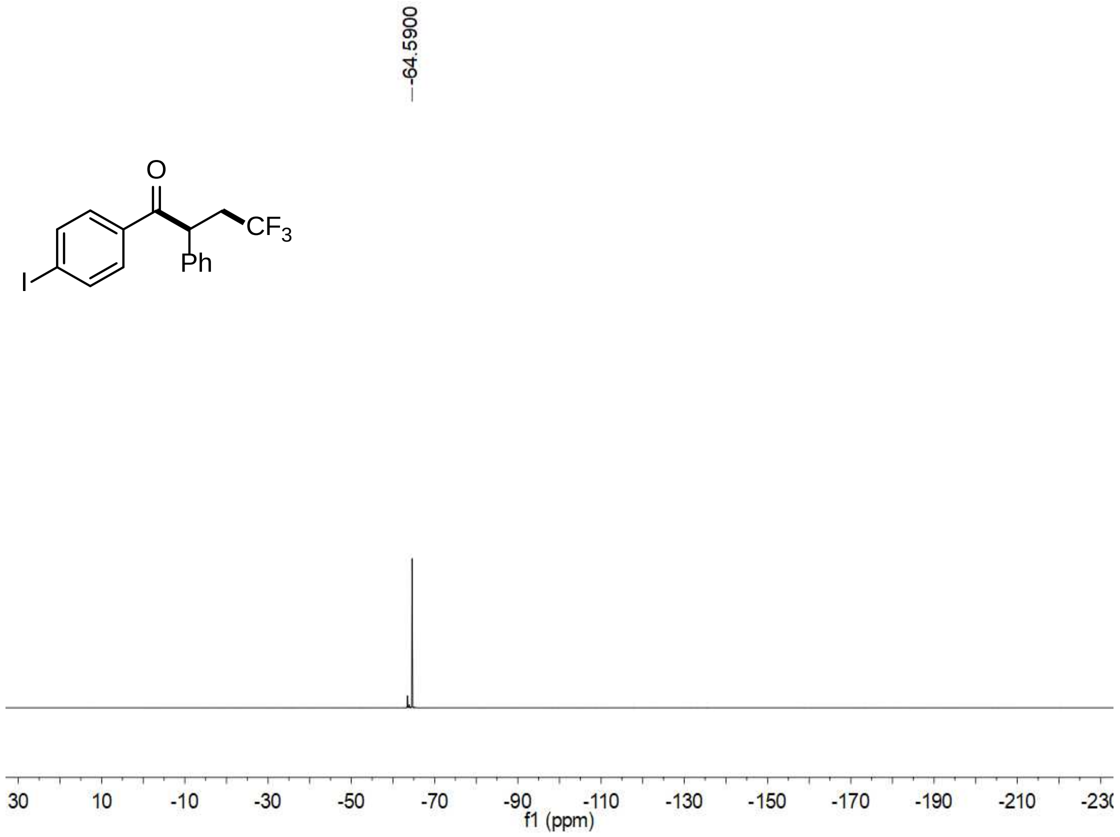

4,4,4-trifluoro-2-phenyl-1-(4-(trifluoromethoxy)phenyl)butan-1-one (3ag)

^{1}H NMR (300 MHz, $CDCl_3$)

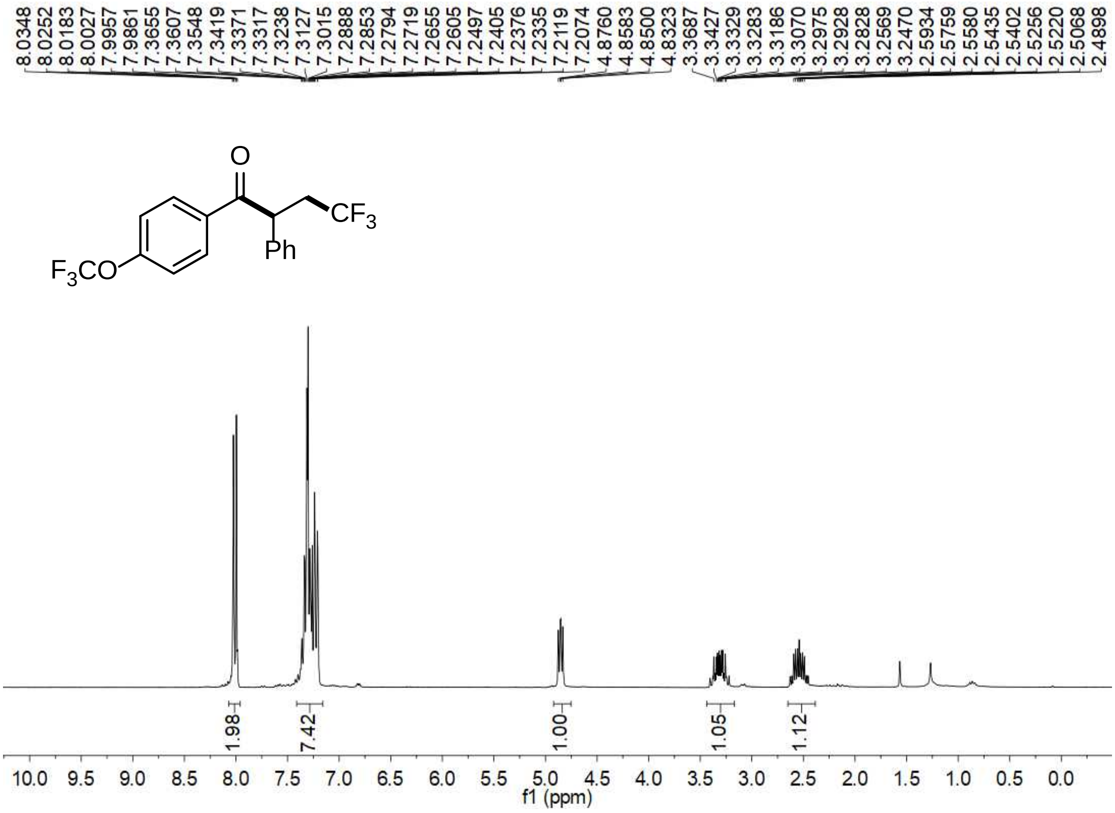

^{13}C NMR (75 MHz, $CDCl_3$)

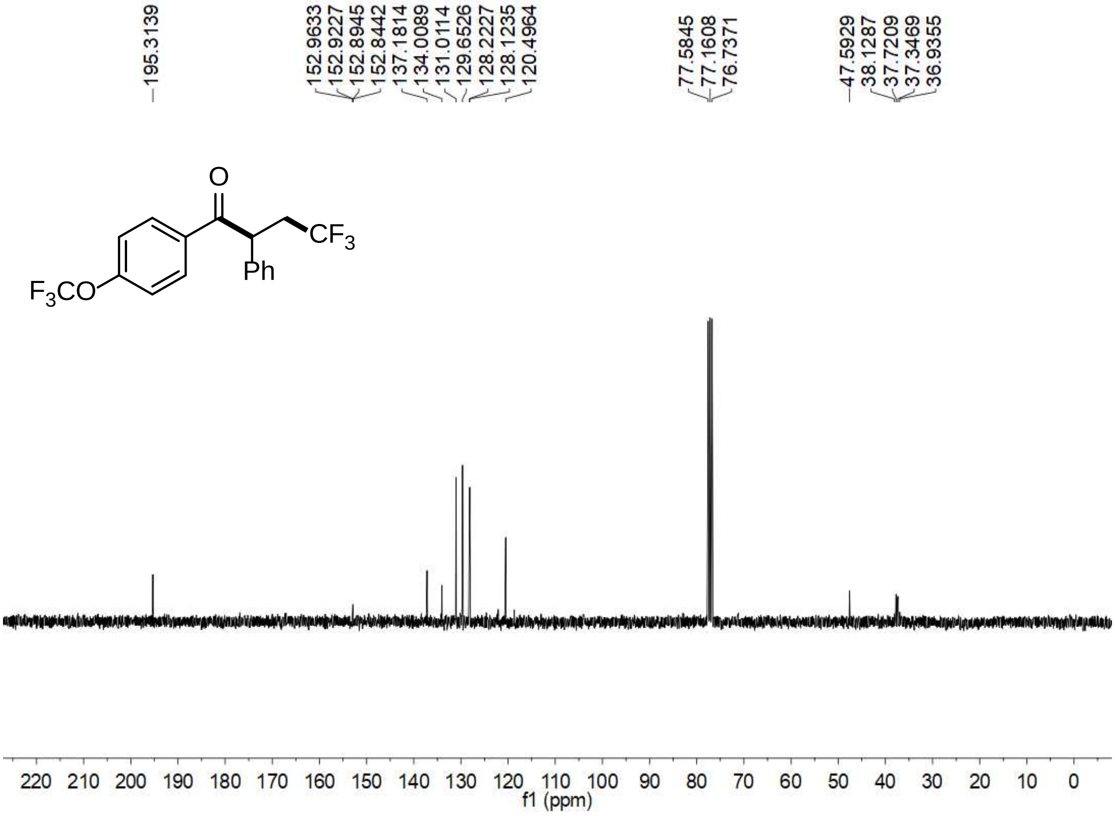

$^{19}F\{^{1}H\}$ NMR (282 MHz, $CDCl_3$)

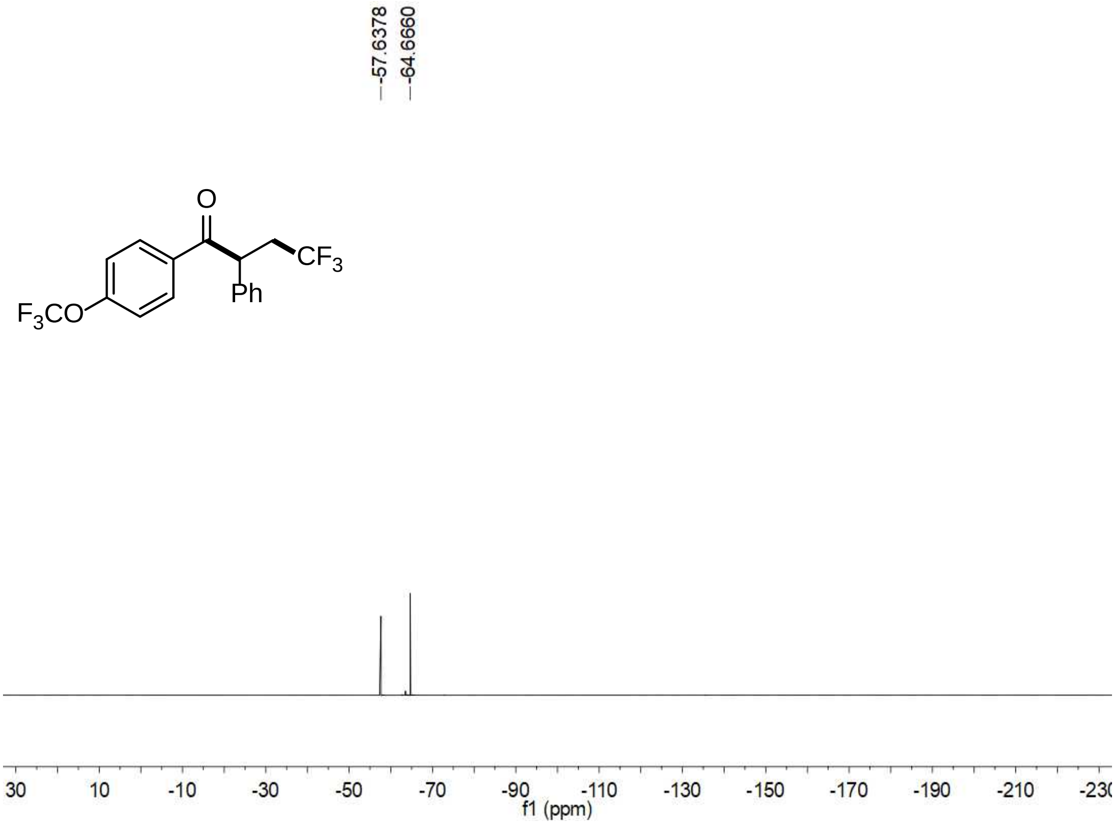

4-(4,4,4-trifluoro-2-phenylbutanoyl)benzonitrile (3ah)

^{1}H NMR (300 MHz, $CDCl_3$)

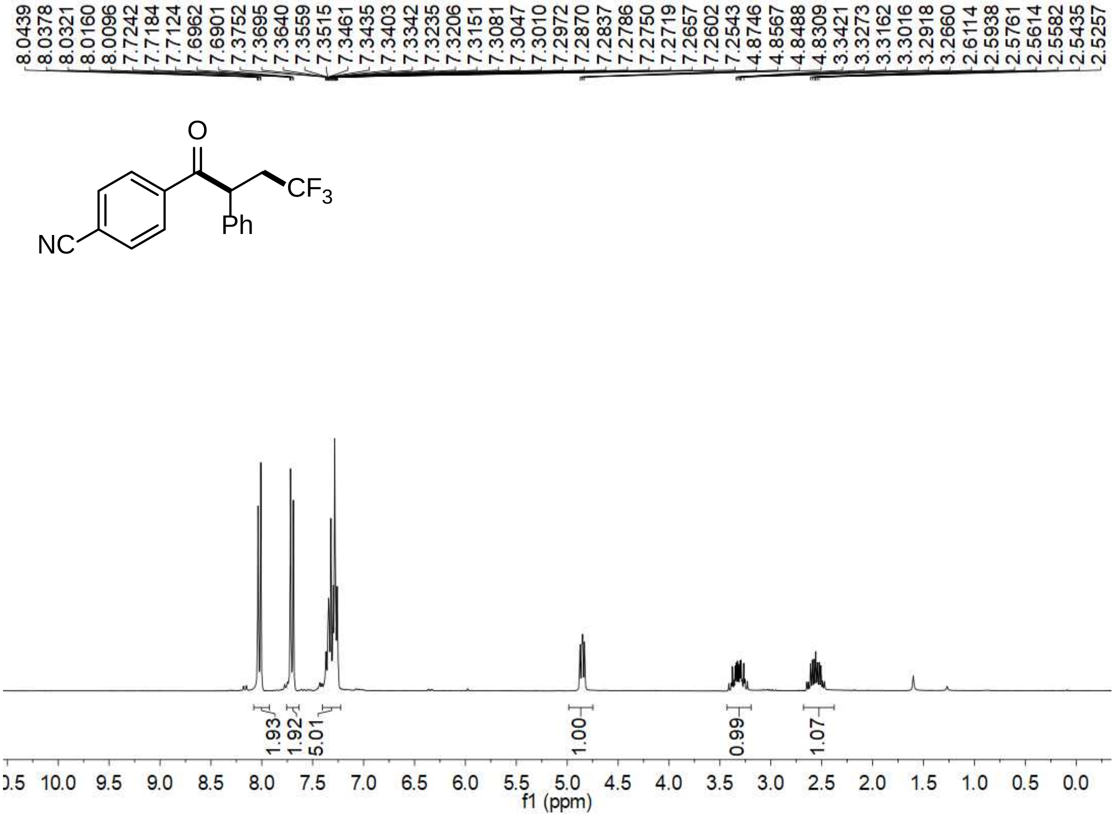

^{13}C NMR (75 MHz, $CDCl_3$)

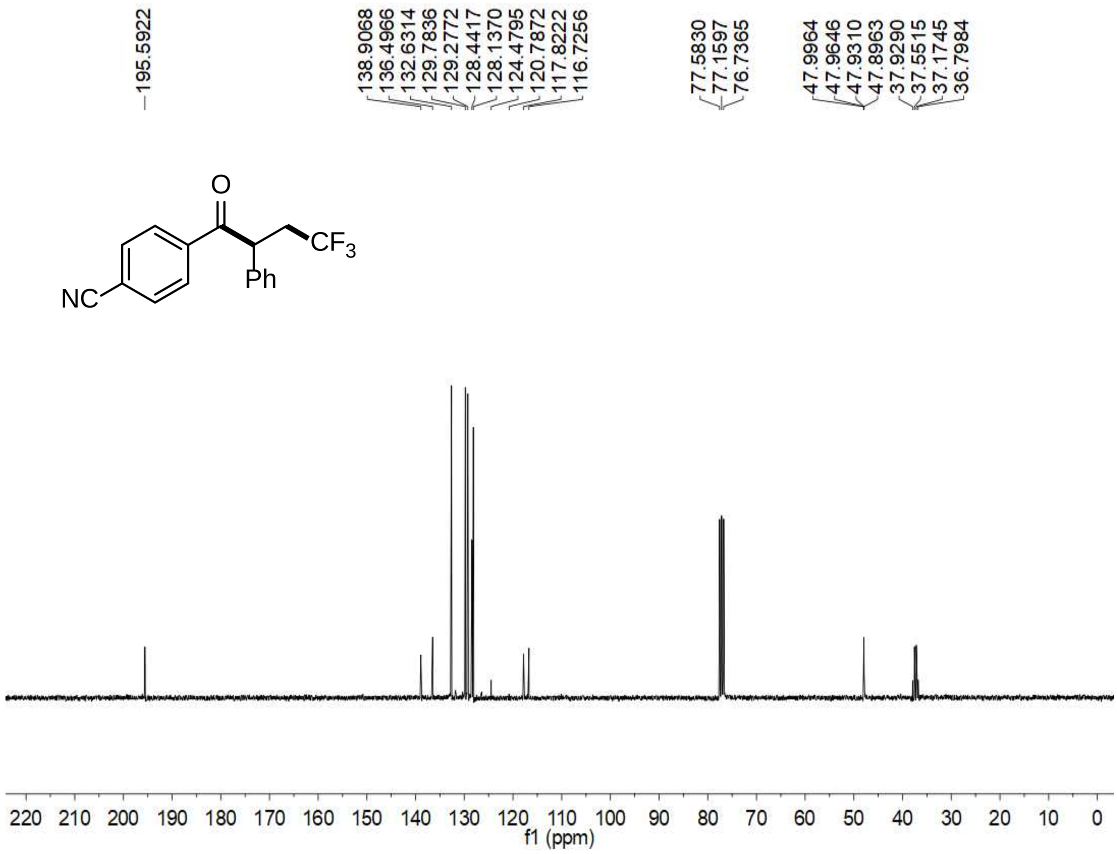

^{19}F {^{1}H} NMR (282 MHz, $CDCl_3$)

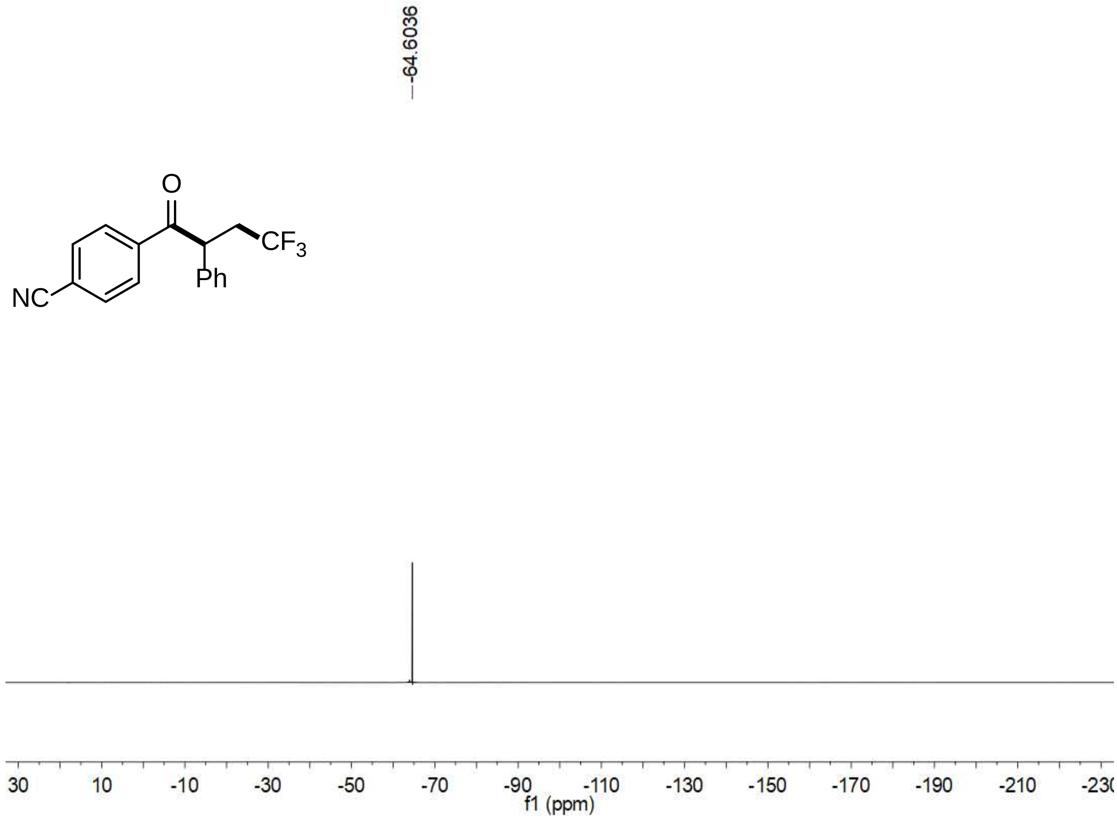

4,4,4-trifluoro-2-phenyl-1-(m-tolyl)butan-1-one (3ai)

^{1}H NMR (300 MHz, $CDCl_3$)

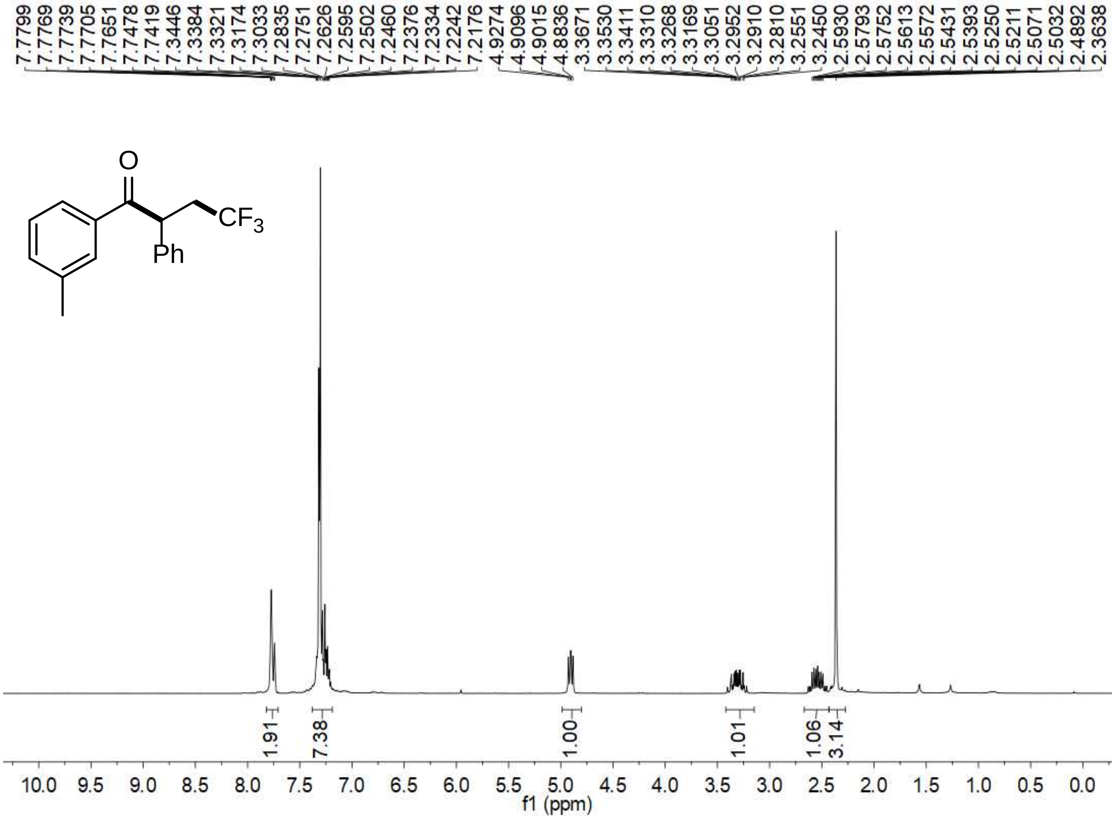

^{13}C NMR (75 MHz, $CDCl_3$)

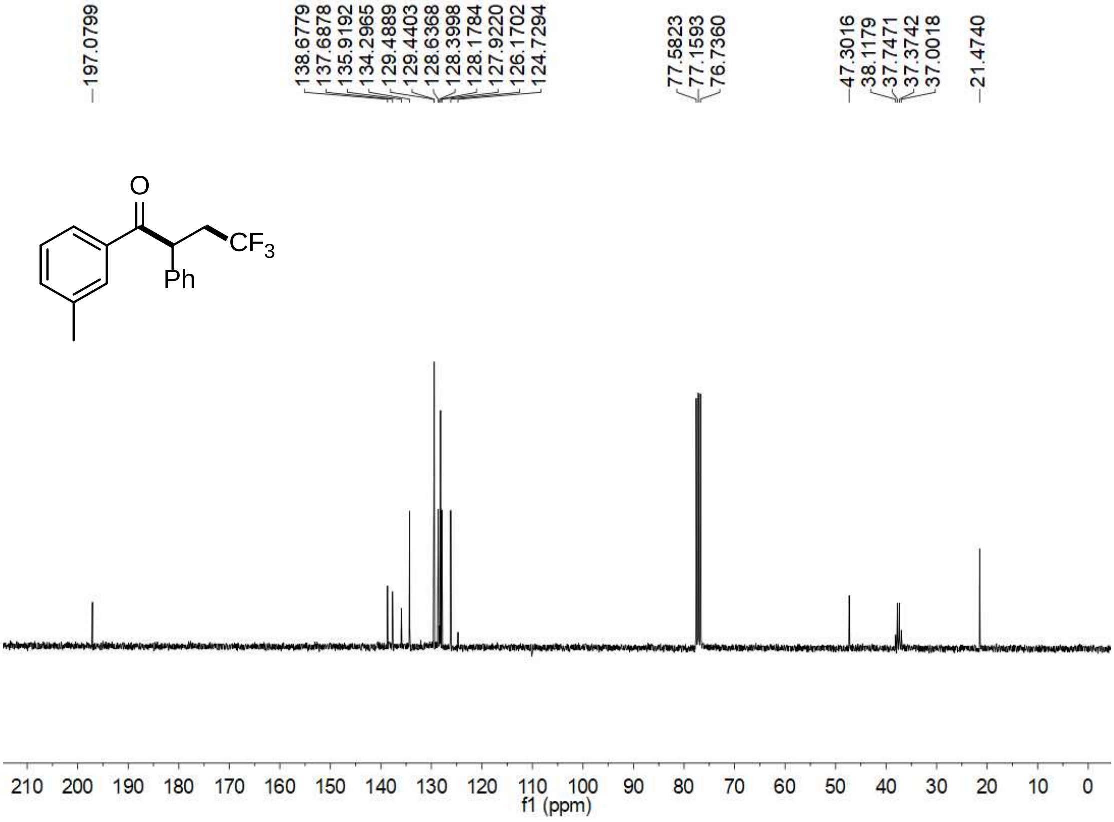

^{19}F {^{1}H} NMR (282 MHz, $CDCl_3$)

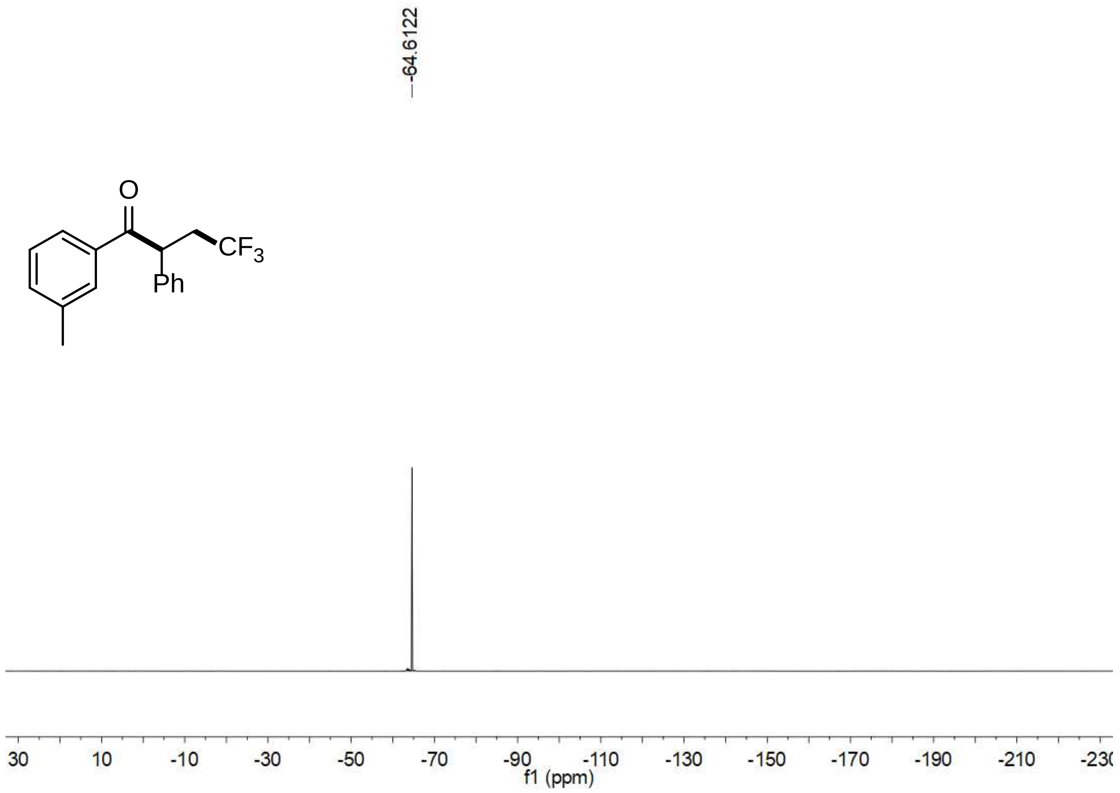

1-(3-bromophenyl)-4,4,4-trifluoro-2-phenylbutan-1-one (3aj)

^{1}H NMR (300 MHz, $CDCl_3$)

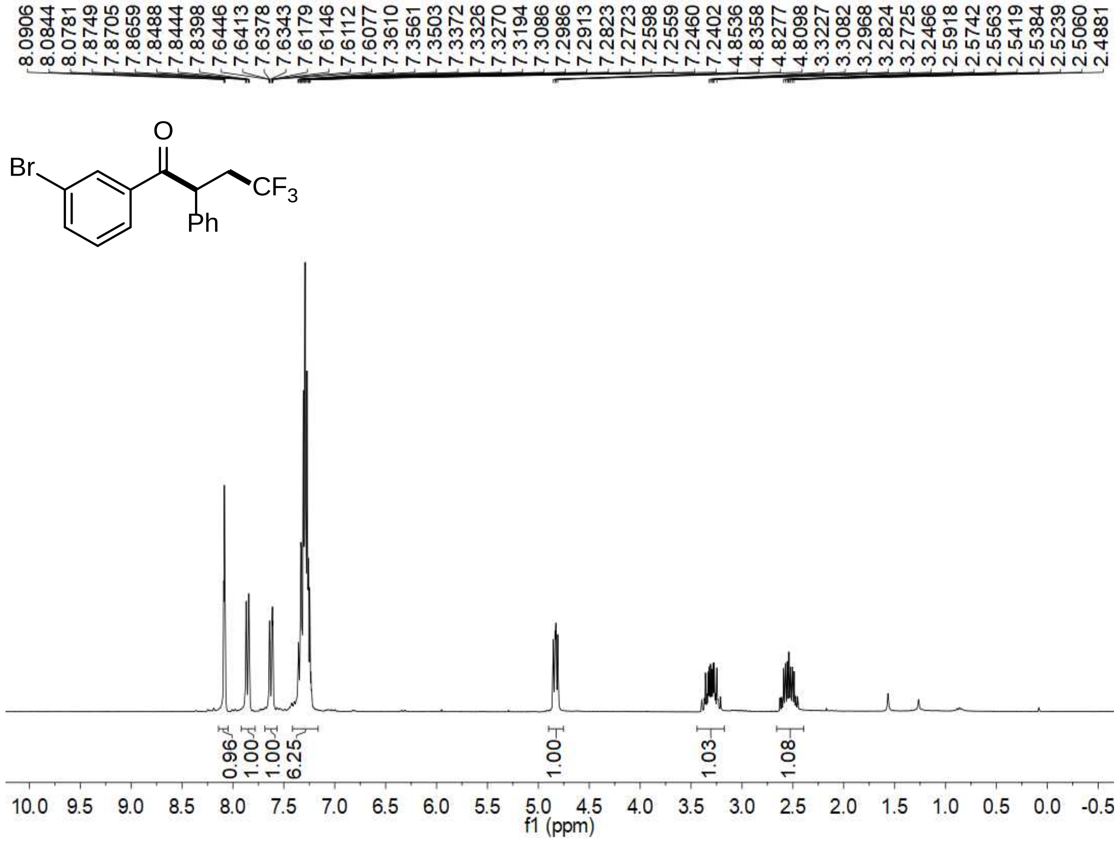

^{13}C NMR (75 MHz, $CDCl_3$)

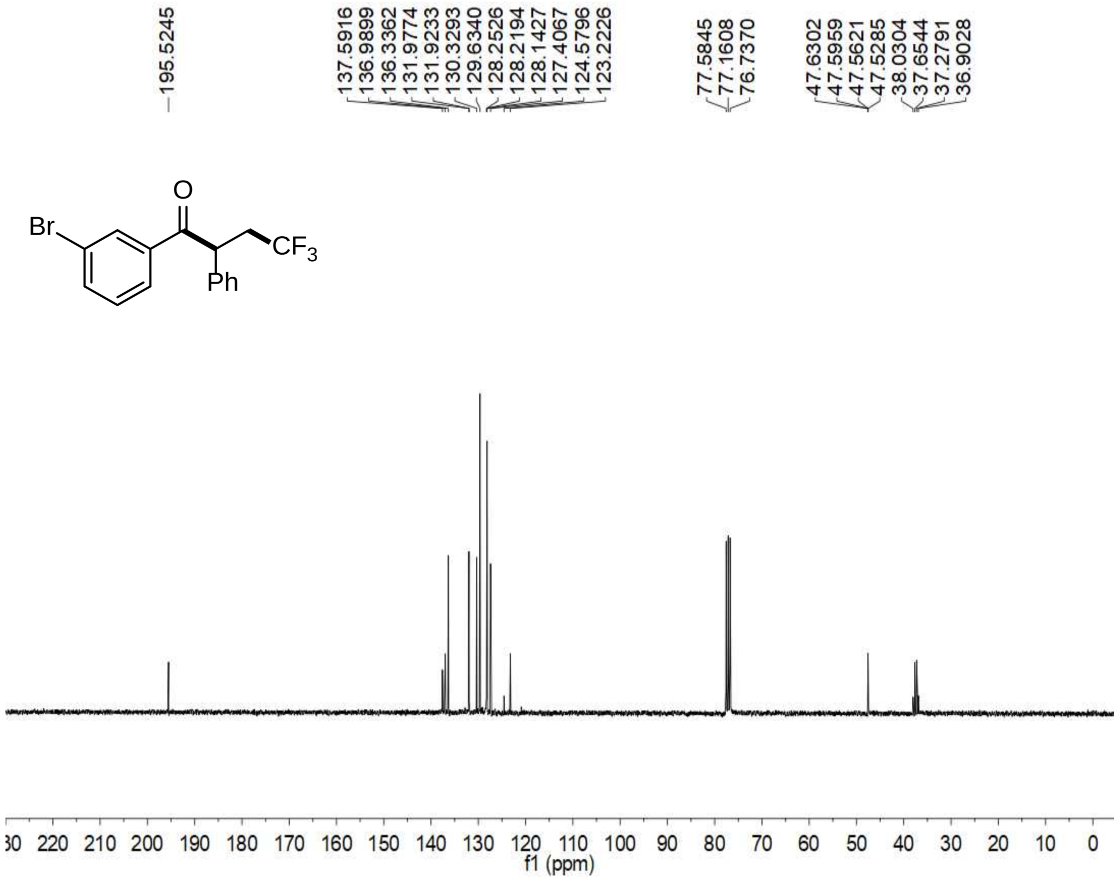

^{19}F {^{1}H} NMR (282 MHz, $CDCl_3$)

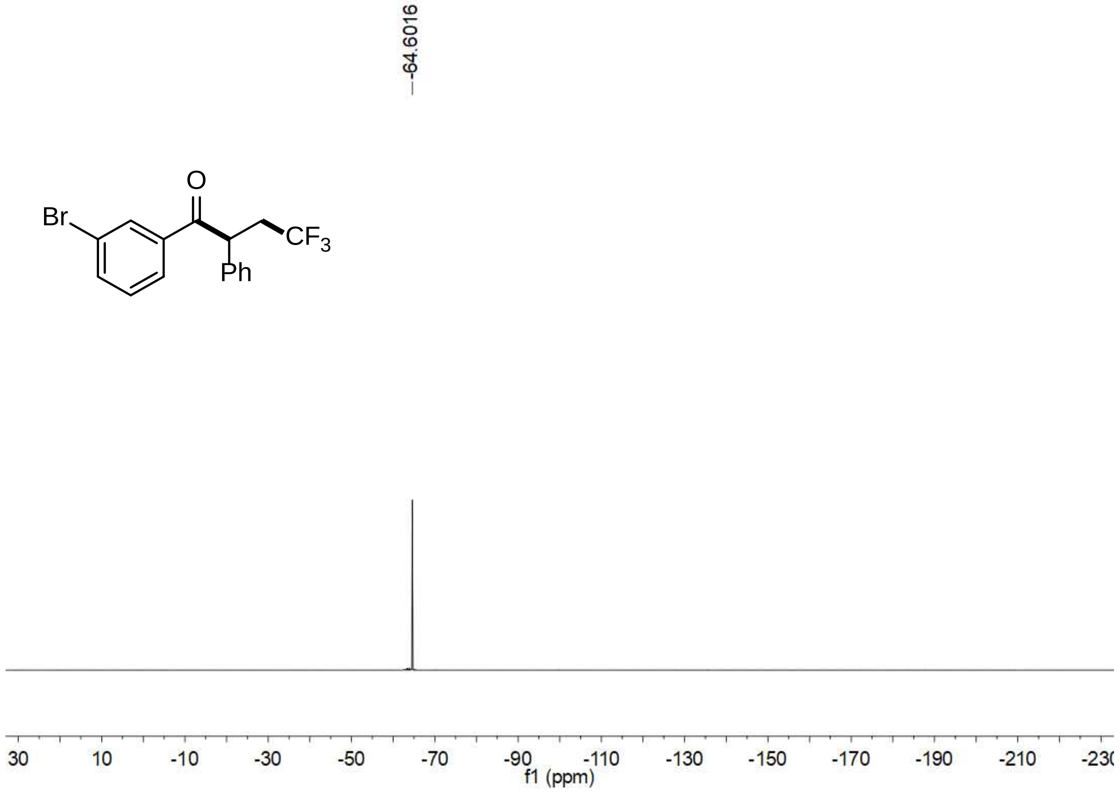

4,4,4-trifluoro-2-phenyl-1-(3-(trifluoromethyl)phenyl)butan-1-one (3ak)

^{1}H NMR (300 MHz, $CDCl_3$)

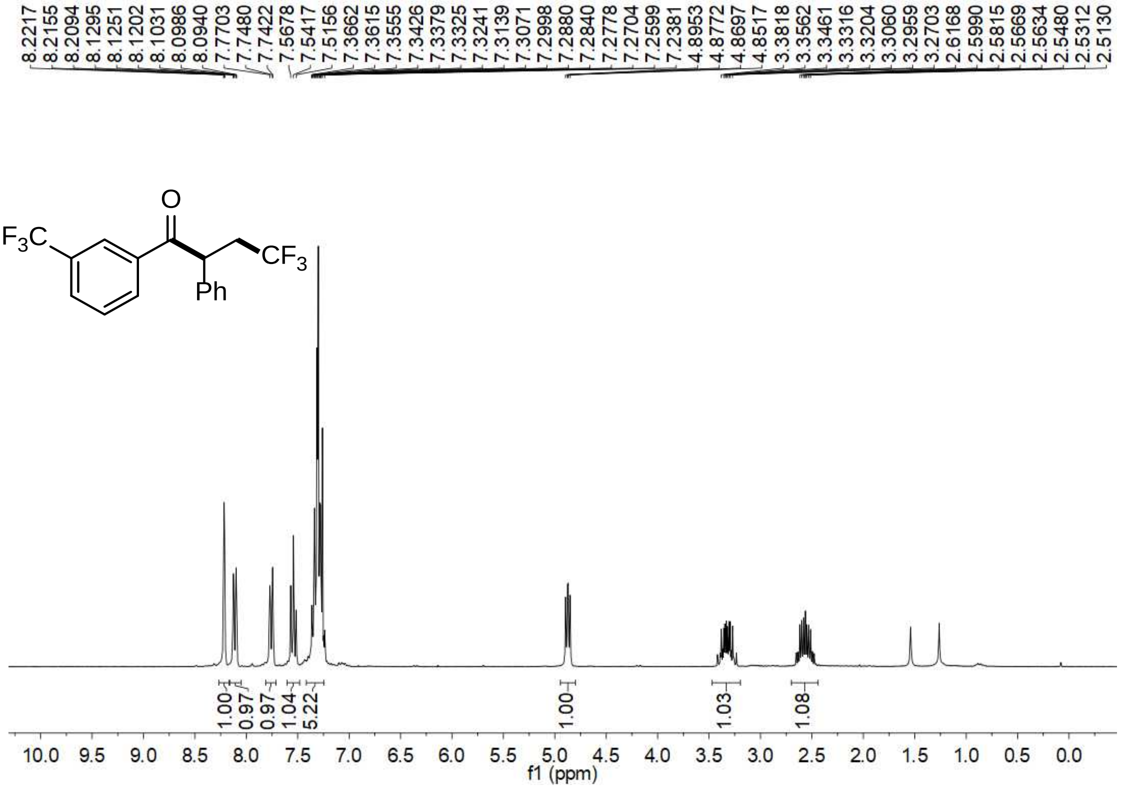

^{13}C NMR (150 MHz, $CDCl_3$)

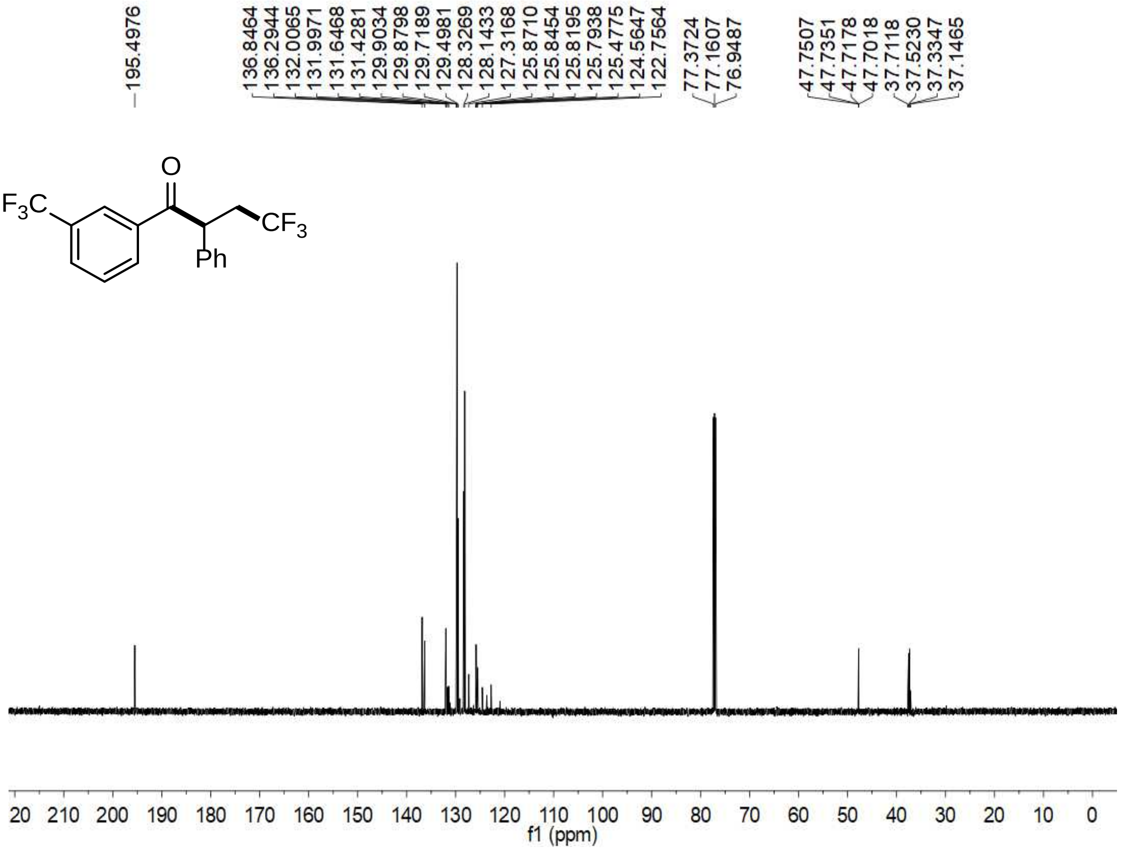

$^{13}C\ \{^{19}F\}$ NMR (150 MHz, $CDCl_3$)

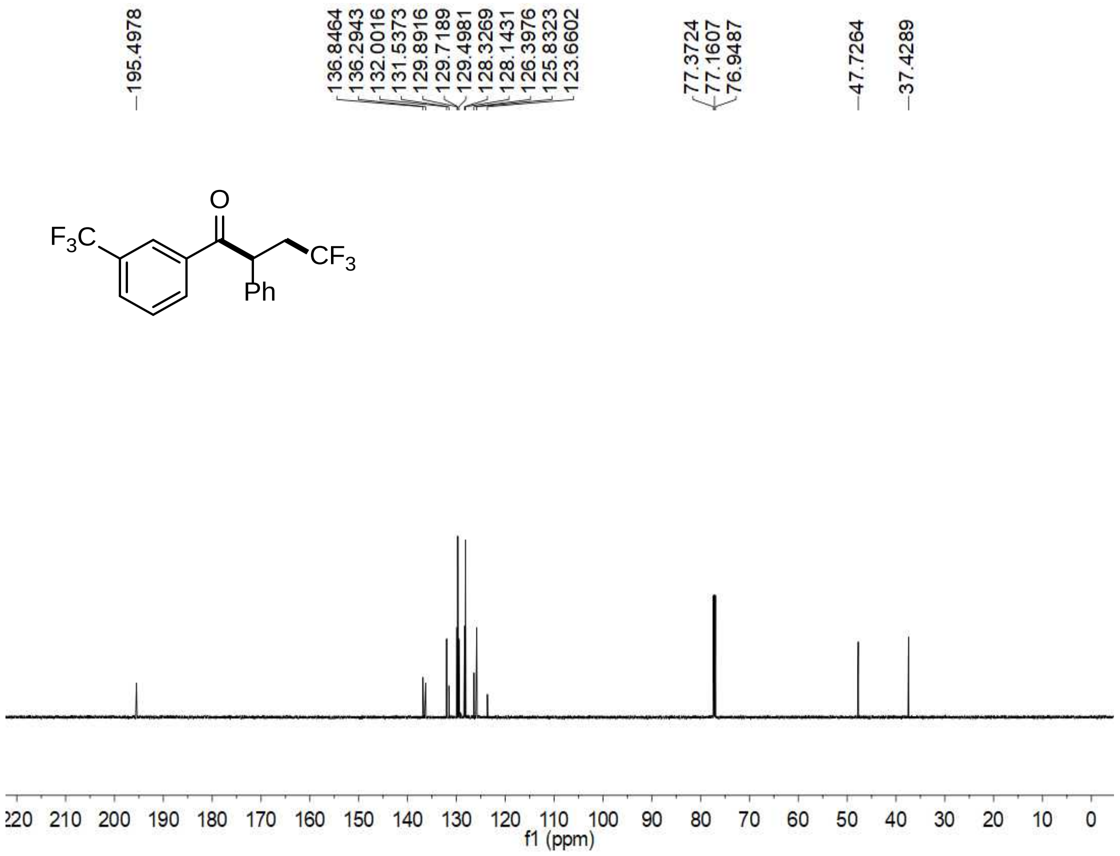

$^{19}F\ \{^{1}H\}$ NMR (282 MHz, $CDCl_3$)

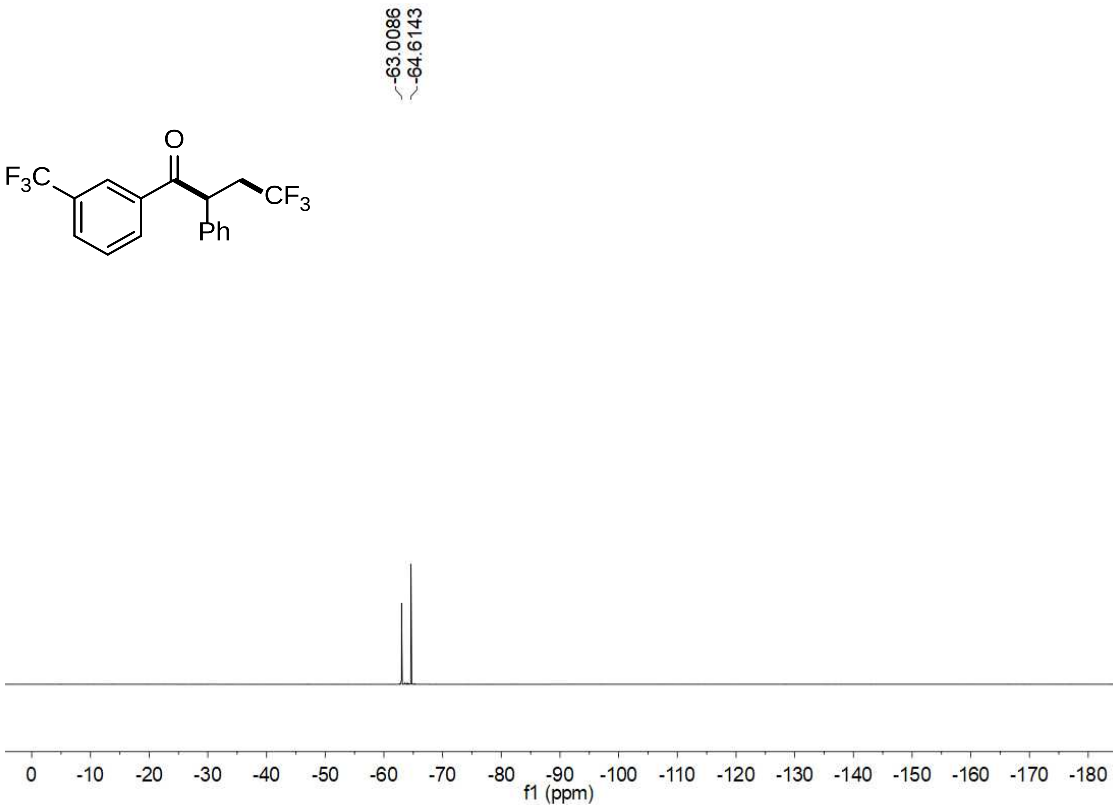

4,4,4-trifluoro-1-(2-fluorophenyl)-2-phenylbutan-1-one (3al)

^{1}H NMR (300 MHz, $CDCl_3$)

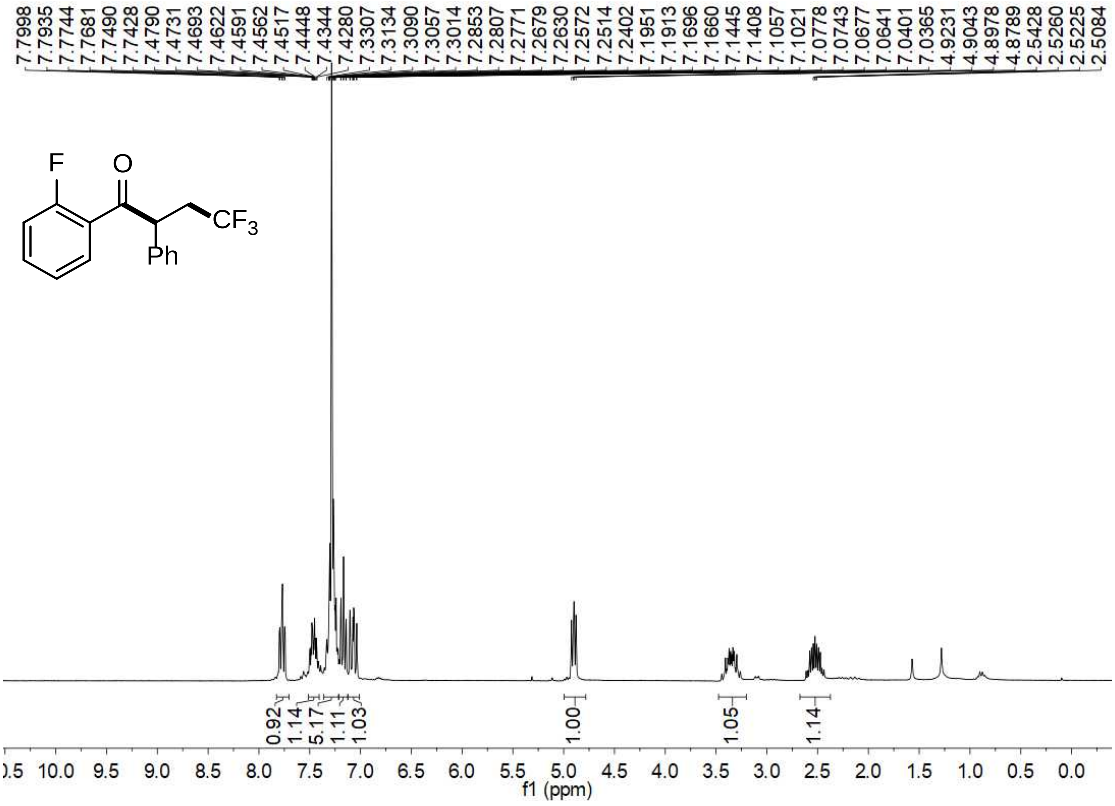

^{13}C NMR (75 MHz, $CDCl_3$)

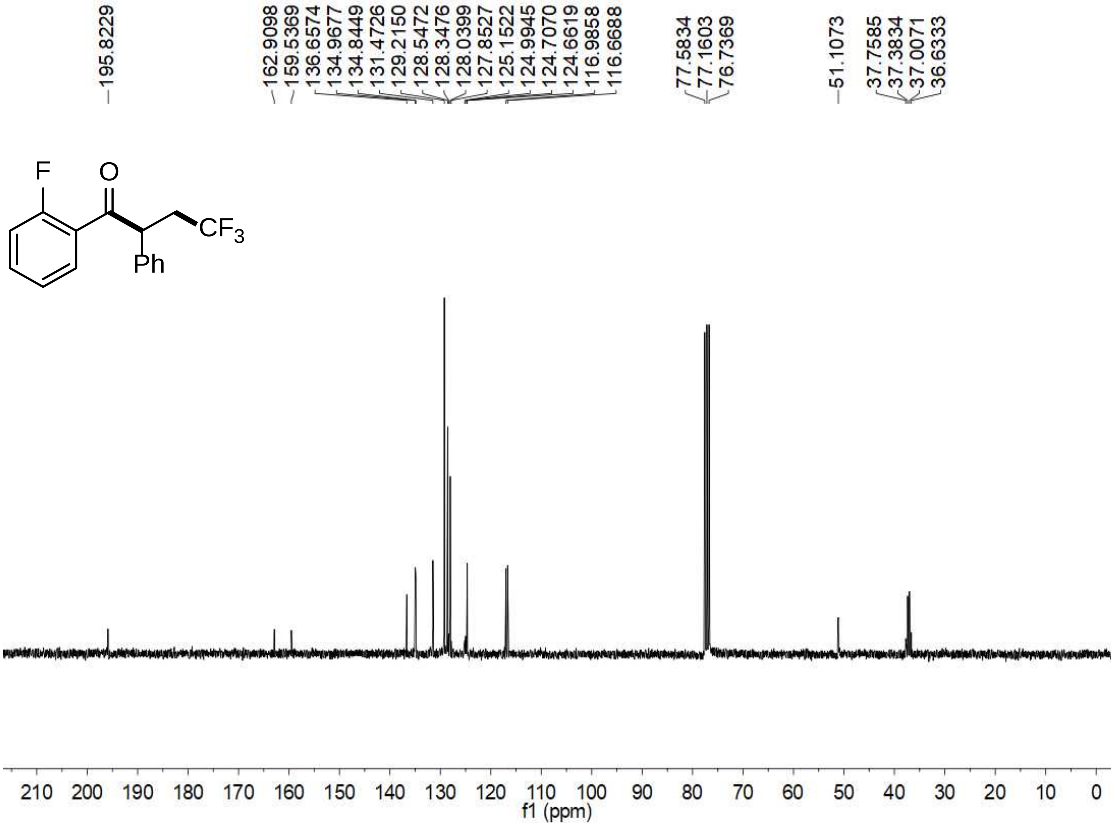

$^{19}F\{^{1}H\}$ NMR (282 MHz, $CDCl_3$)

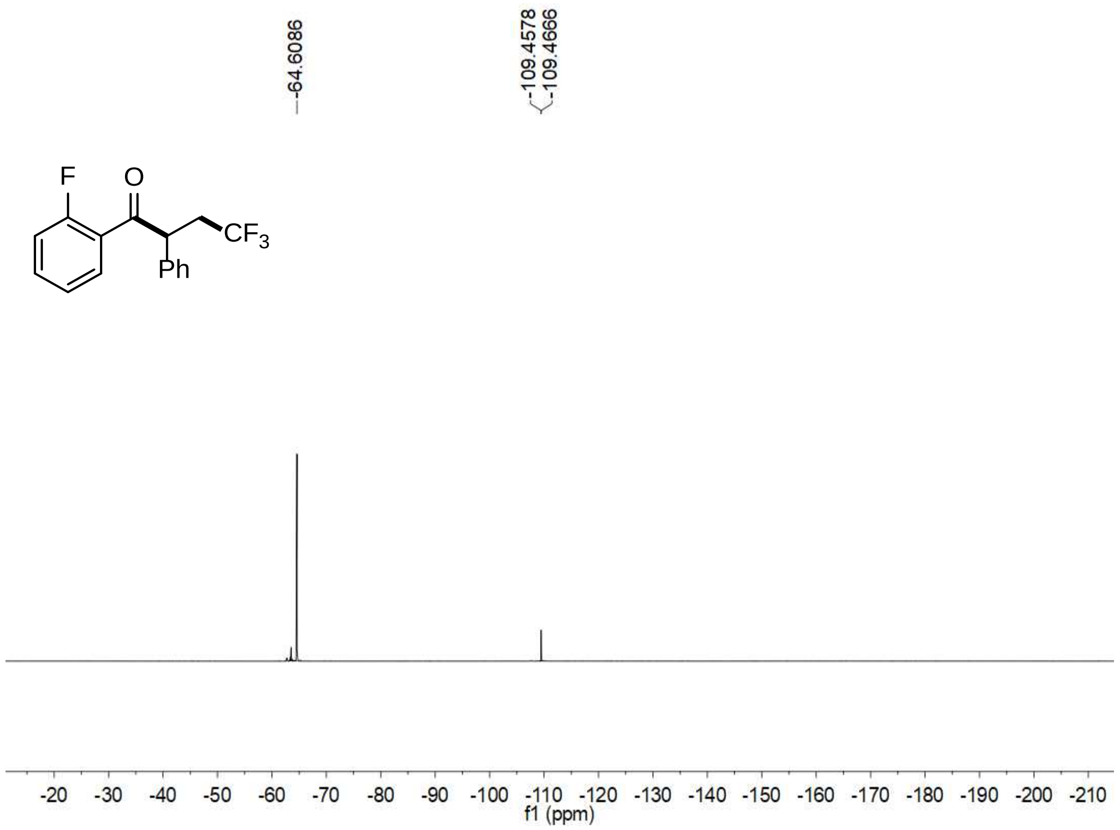

1-(2-bromophenyl)-4,4,4-trifluoro-2-phenylbutan-1-one (3am)

^{1}H NMR (300 MHz, $CDCl_3$)

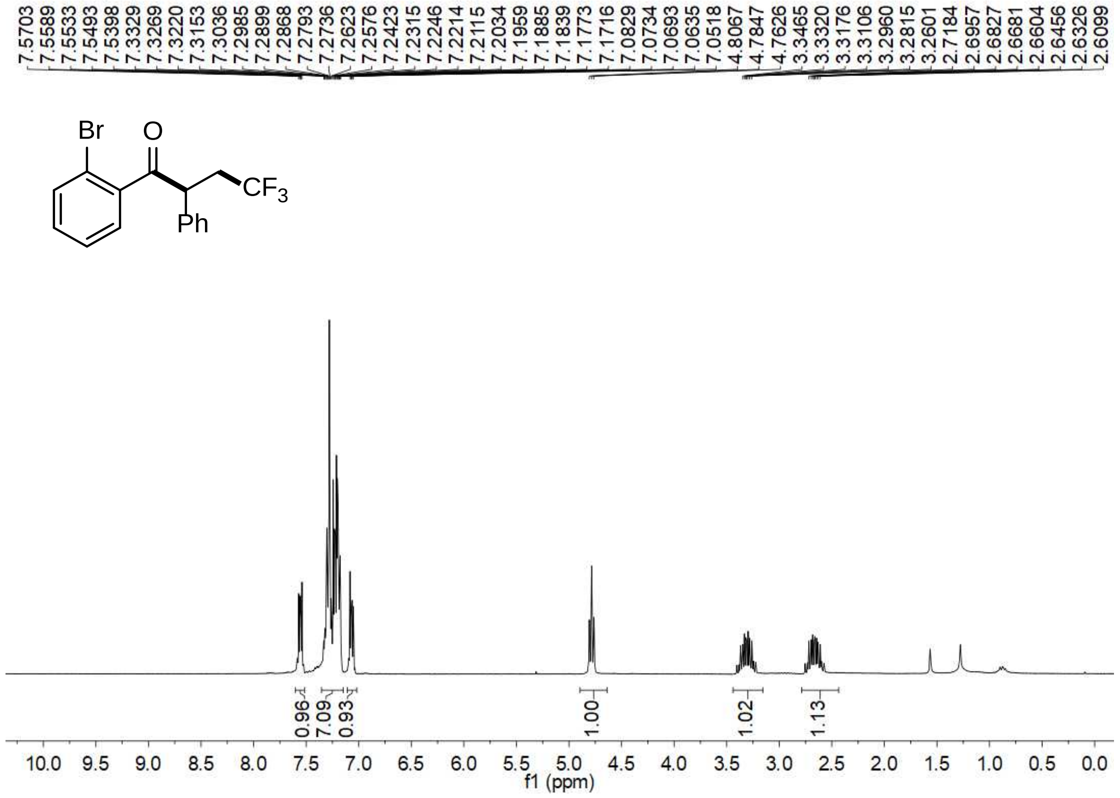

^{13}C NMR (75 MHz, $CDCl_3$)

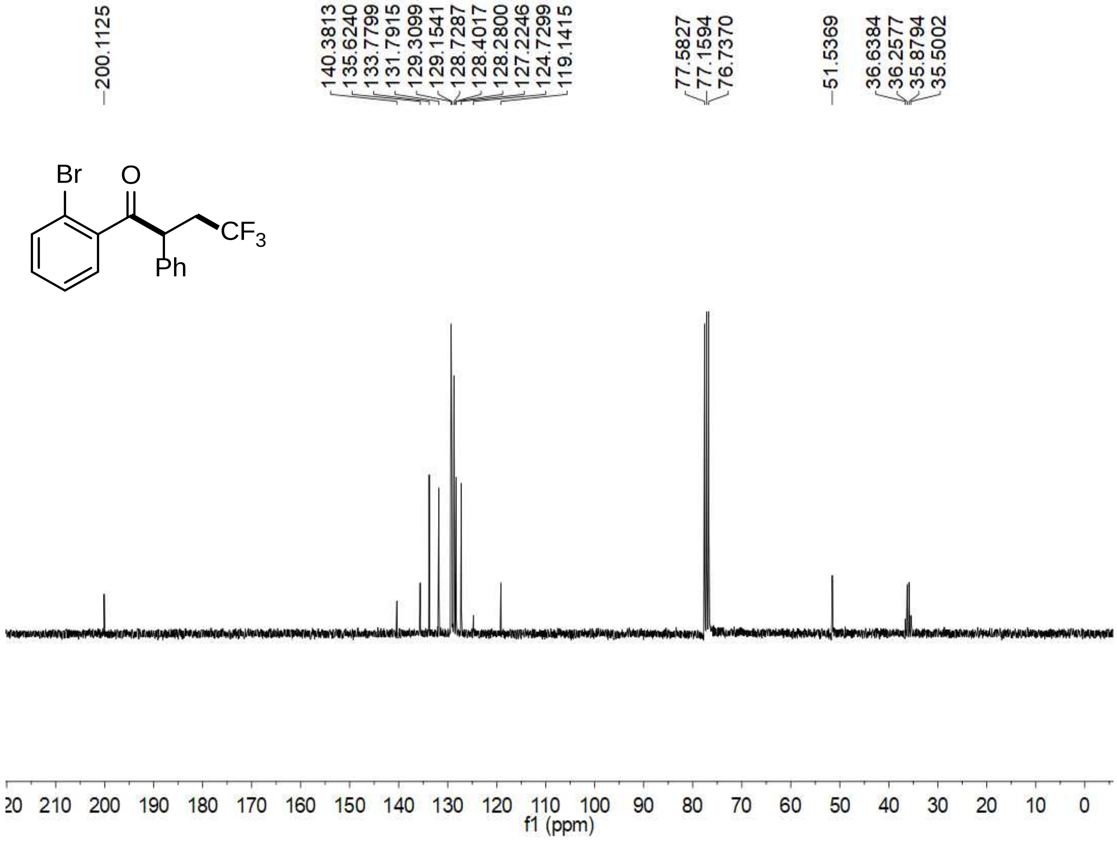

^{19}F {^{1}H} NMR (282 MHz, $CDCl_3$)

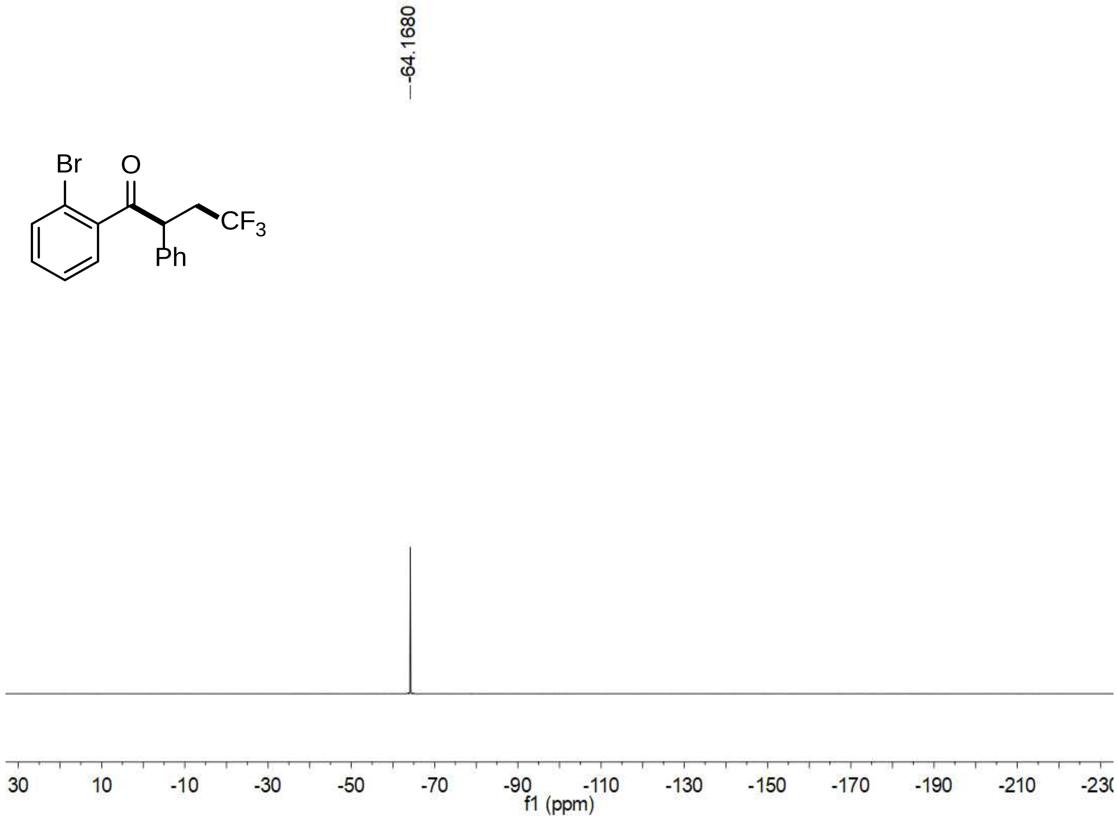

1-(2-(tert-butyl)phenyl)-4,4,4-trifluoro-2-phenylbutan-1-one (3an)

^{1}H NMR (300 MHz, $CDCl_3$)

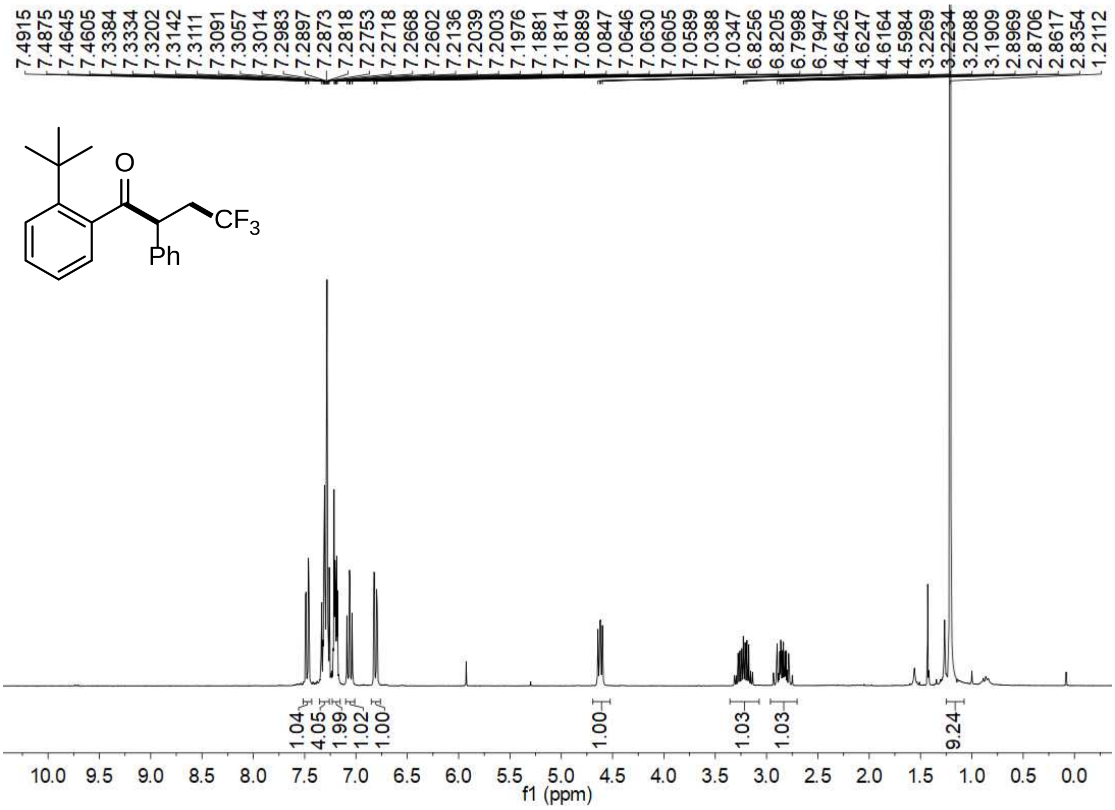

^{13}C NMR (75 MHz, $CDCl_3$)

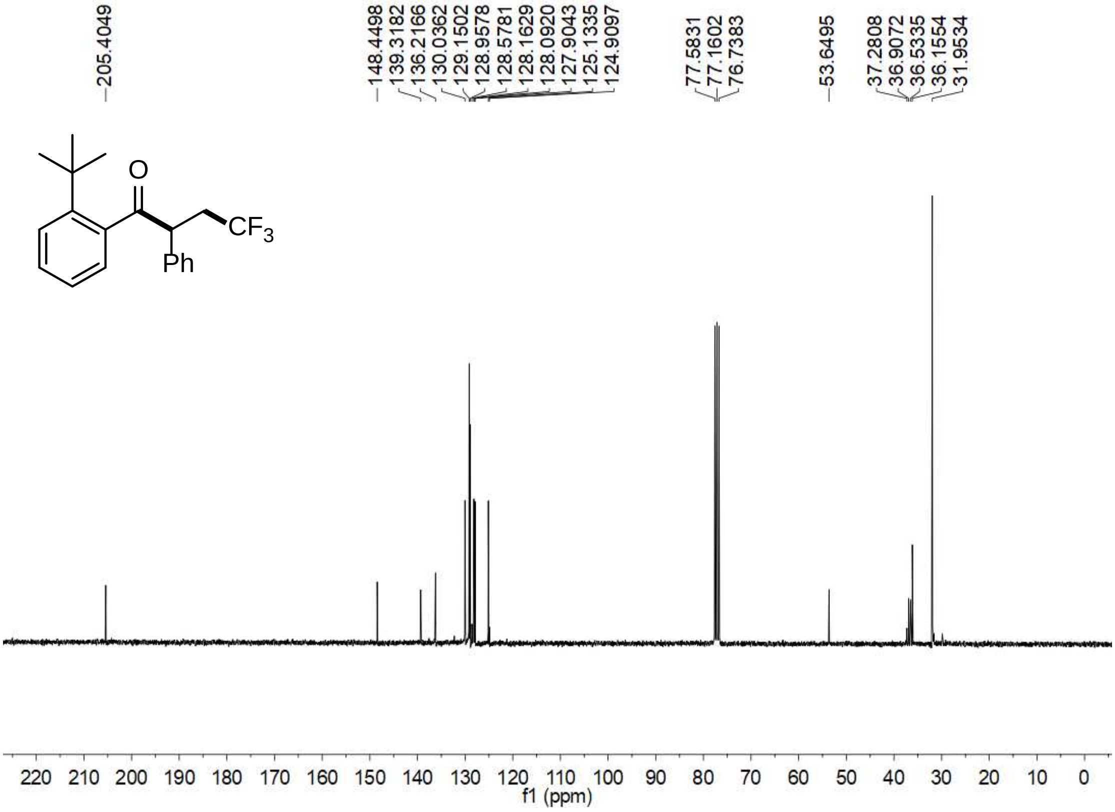

$^{19}F\{^{1}H\}$ NMR (282 MHz, $CDCl_3$)

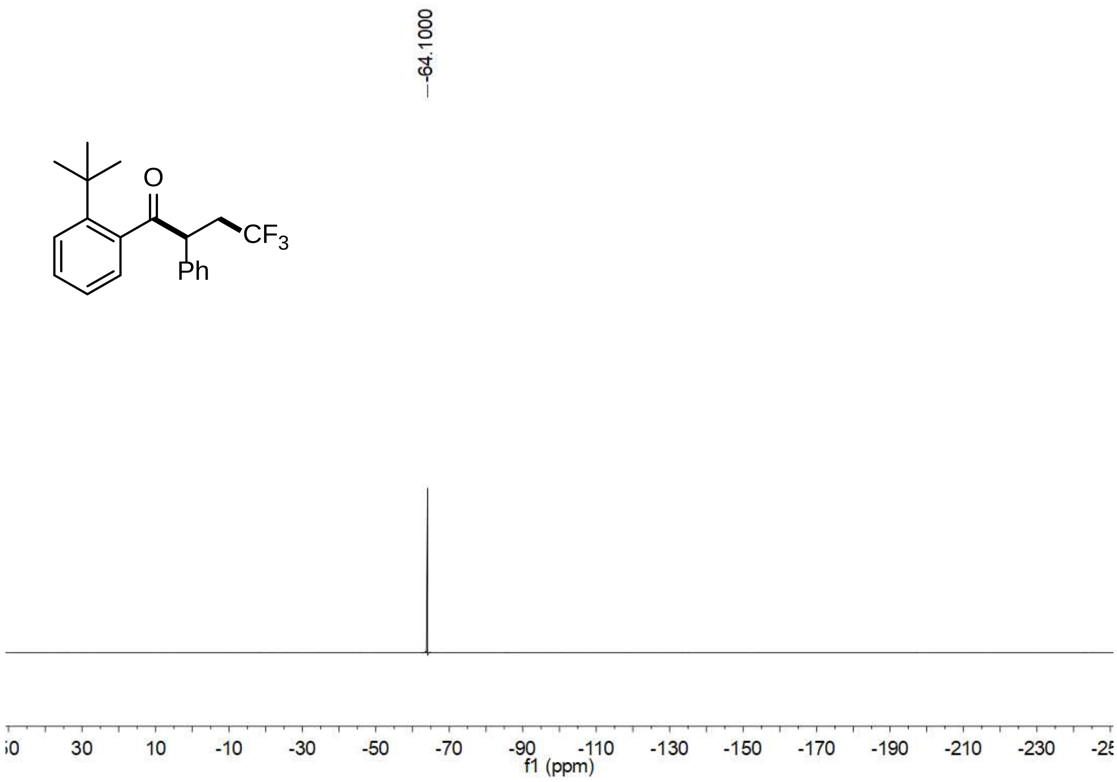

4,4,4-trifluoro-2-(naphthalen-2-yl)-1-phenylbutan-1-one (3ao)

^{1}H NMR (300 MHz, $CDCl_3$)

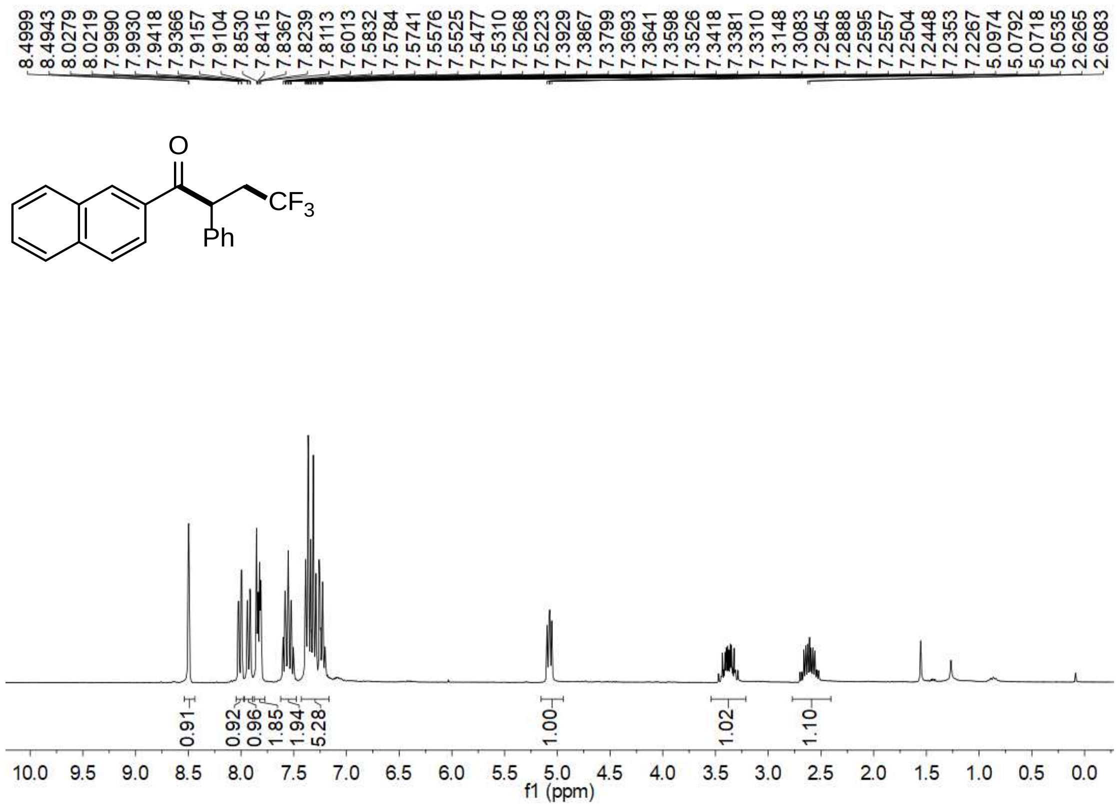

^{13}C NMR (75 MHz, $CDCl_3$)

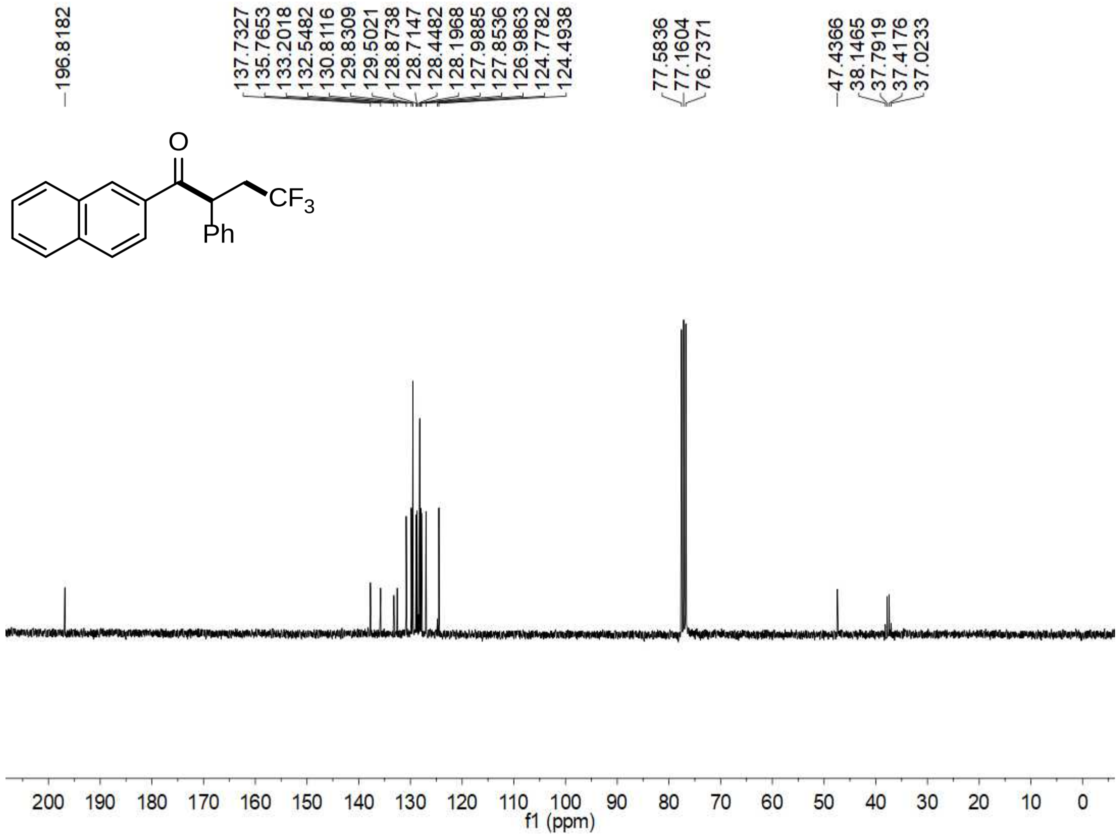

^{19}F {^{1}H} NMR (282 MHz, $CDCl_3$)

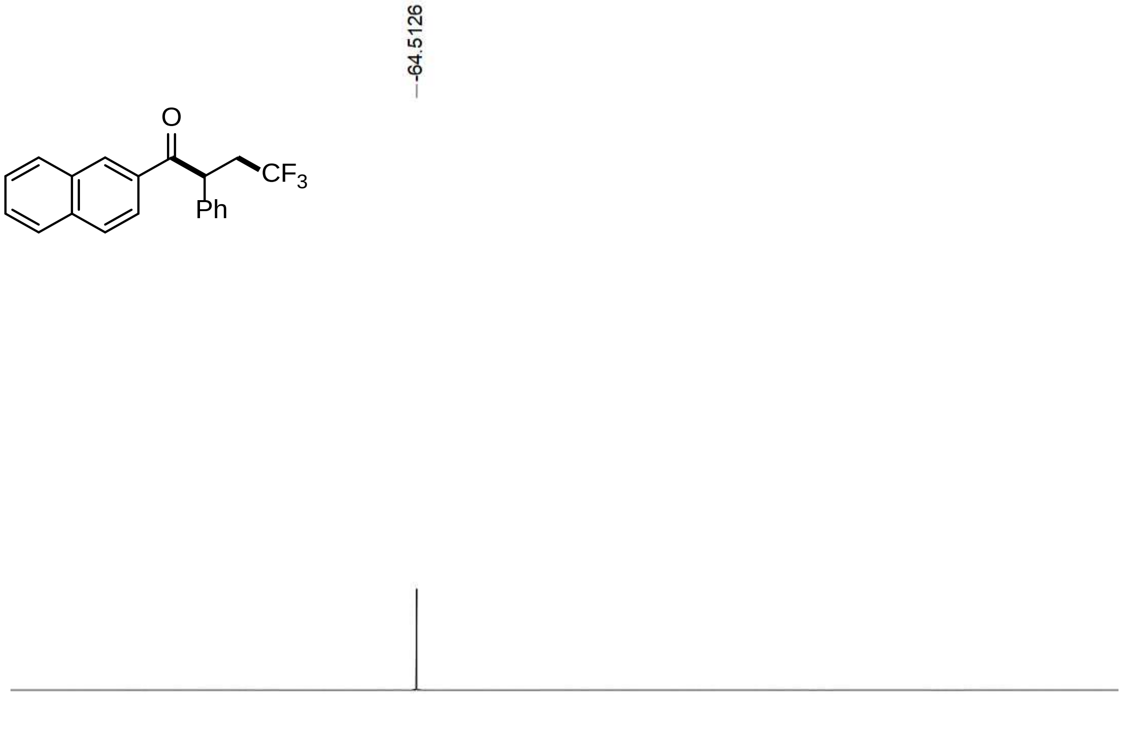

4,4,4-trifluoro-1-(furan-2-yl)-2-phenylbutan-1-one (3ap)

^{1}H NMR (300 MHz, $CDCl_3$)

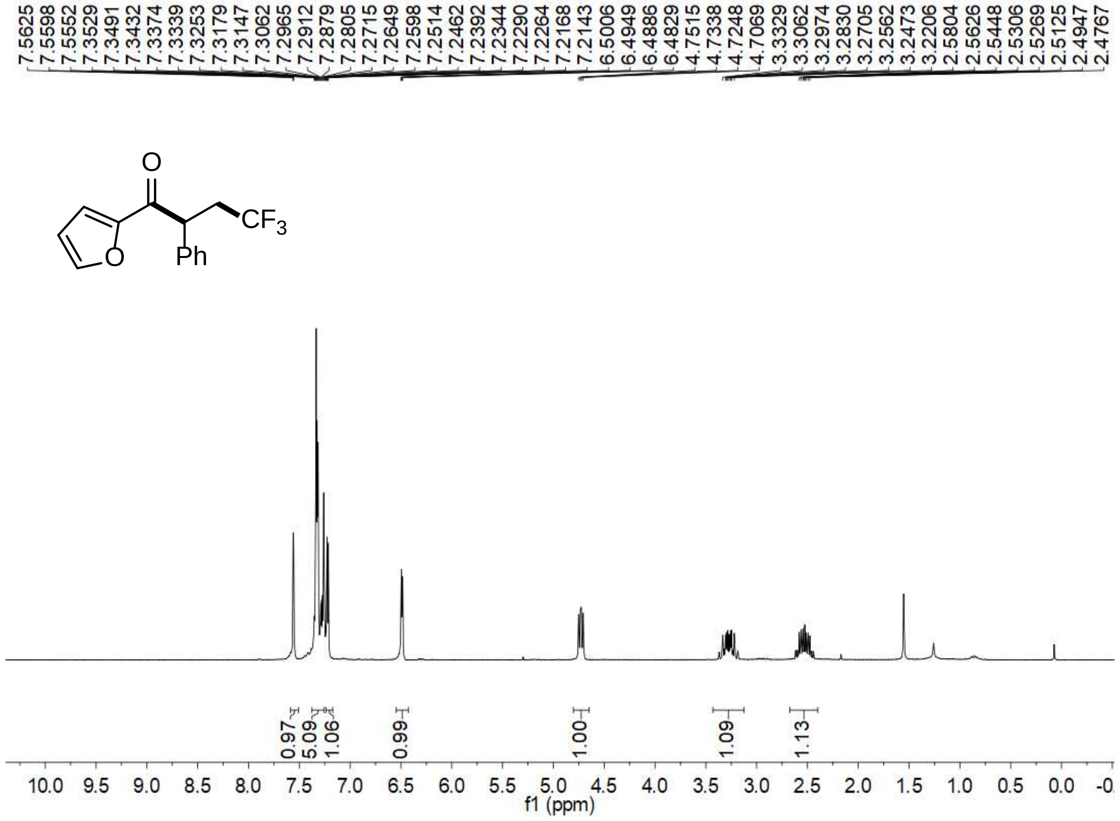

^{13}C NMR (75 MHz, $CDCl_3$)

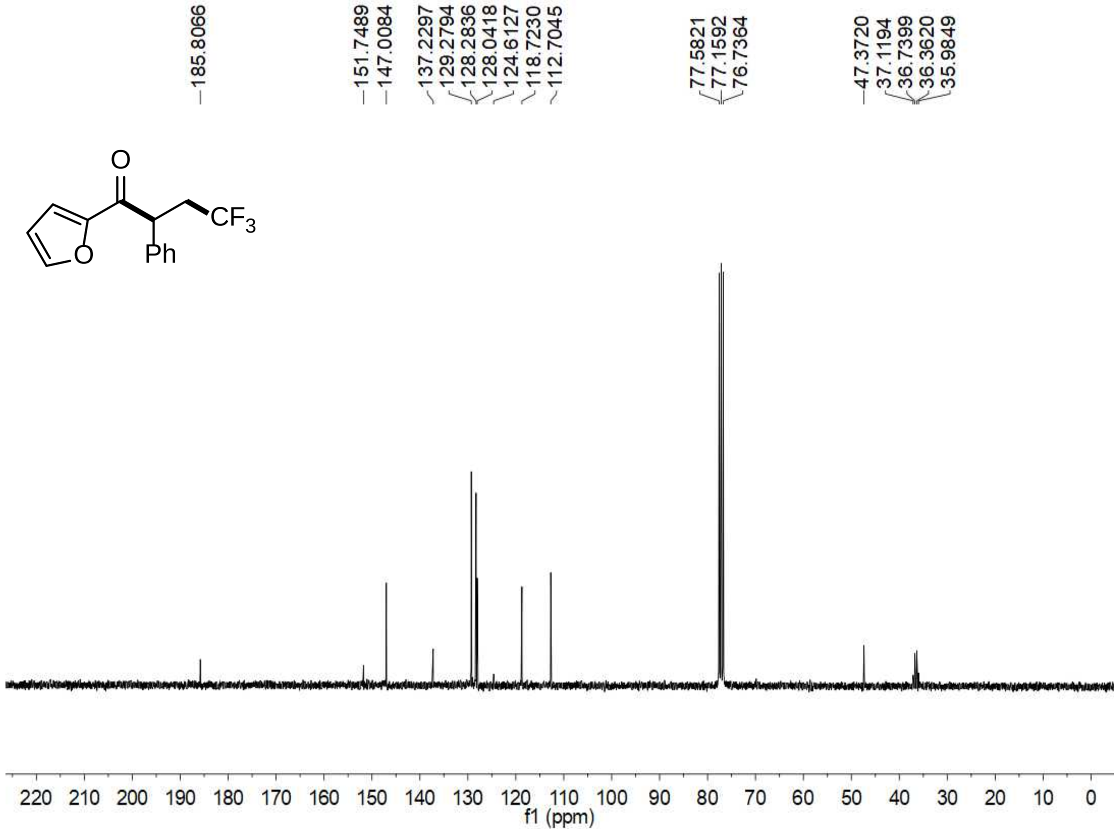

^{19}F {^{1}H} NMR (282 MHz, $CDCl_3$)

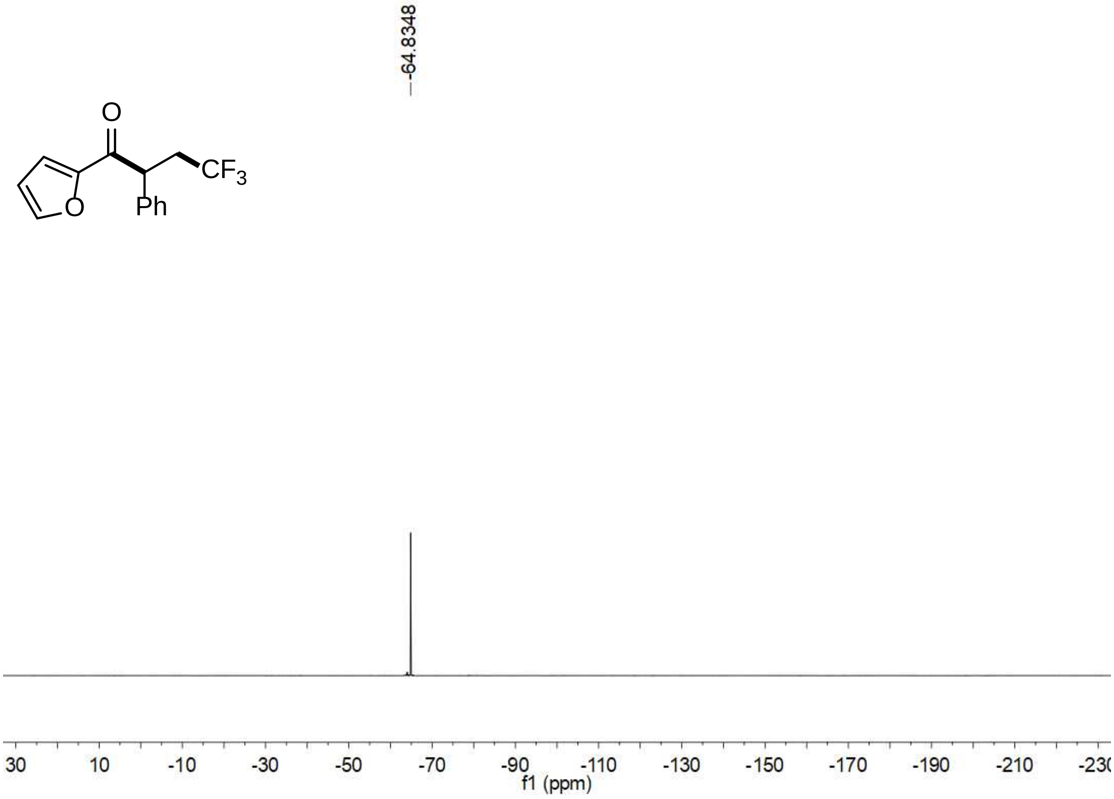

4,4,4-trifluoro-2-phenyl-1-(thiophen-2-yl)butan-1-one (3aq)

^{1}H NMR (300 MHz, $CDCl_3$)

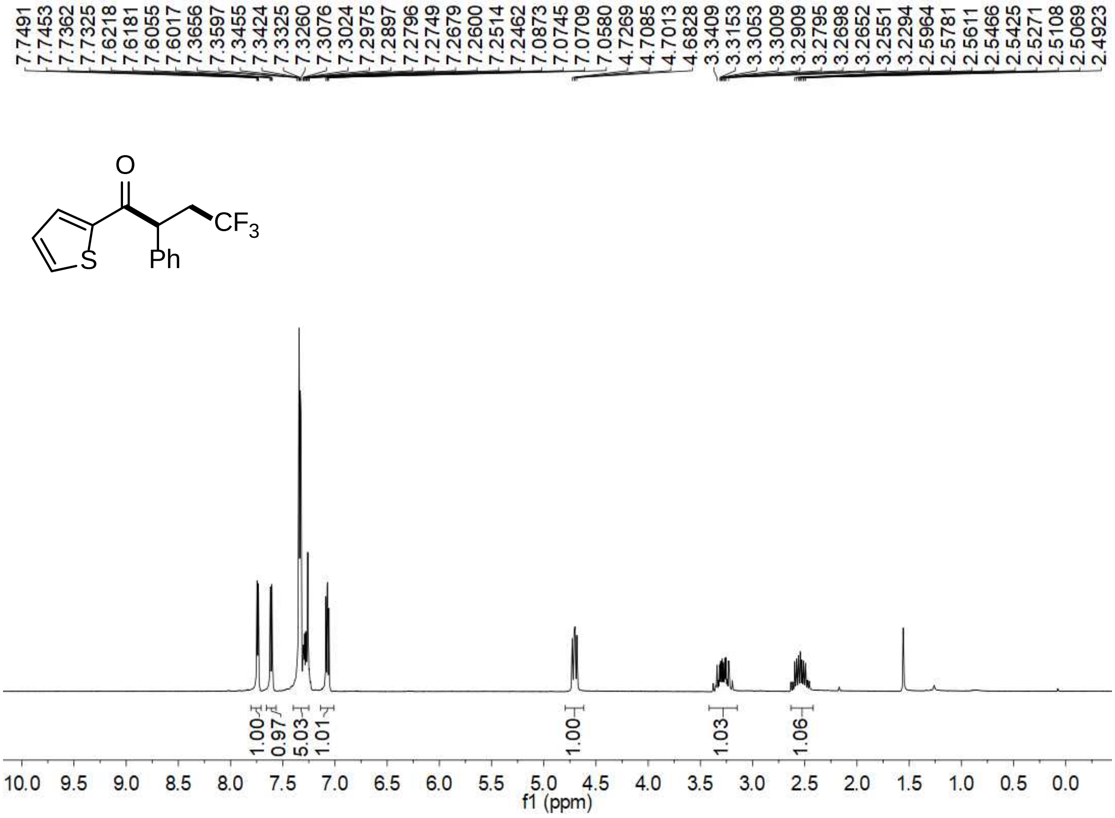

^{13}C NMR (75 MHz, $CDCl_3$)

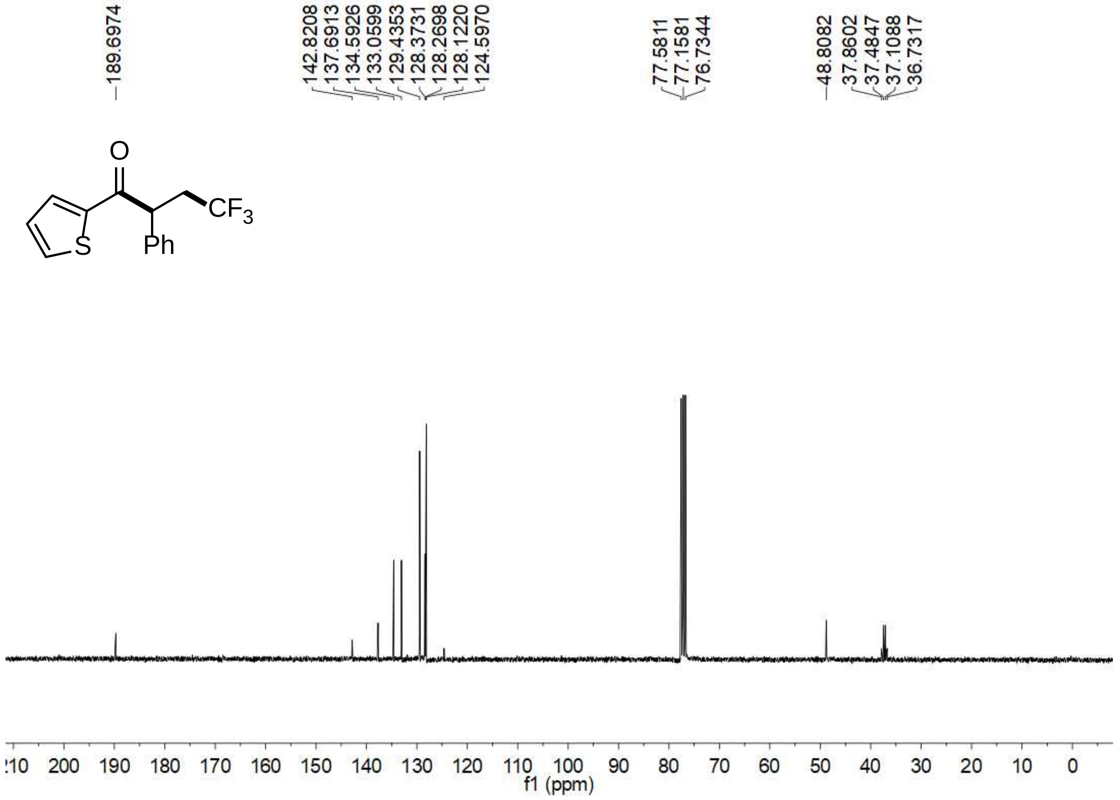

^{19}F {^{1}H} NMR (282 MHz, $CDCl_3$)

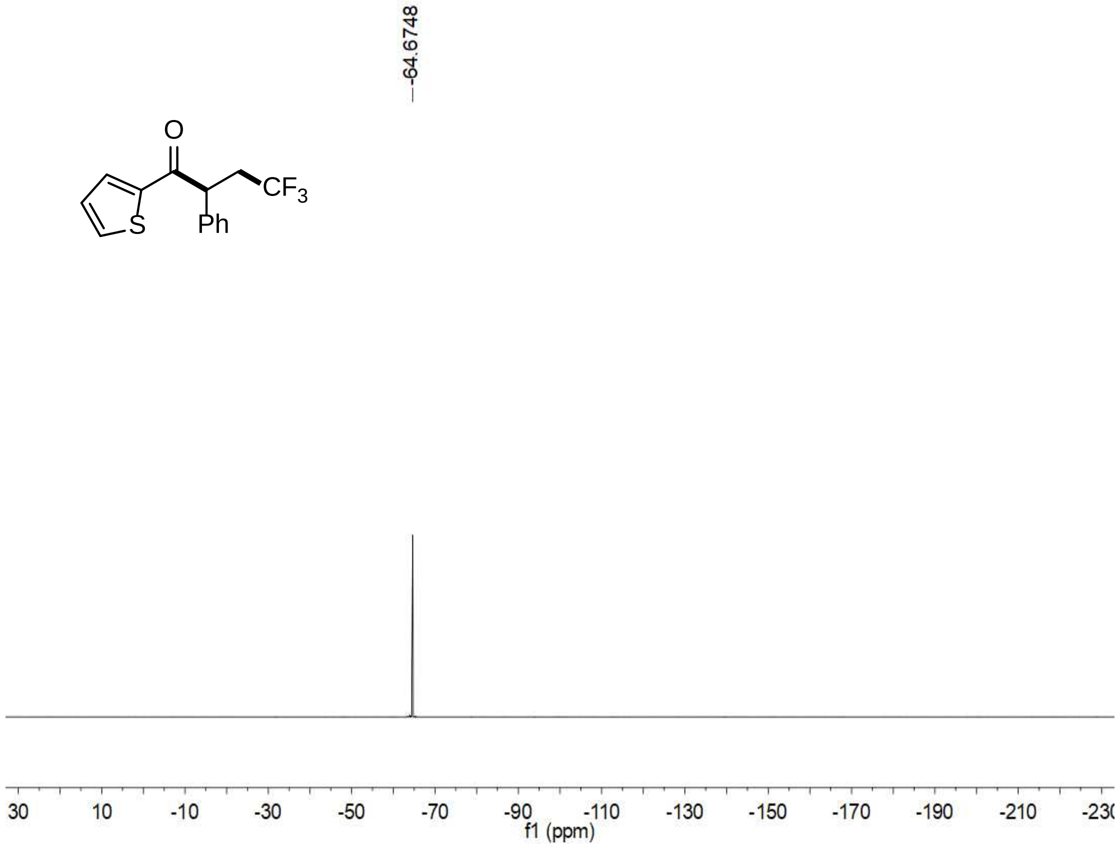

4,4,4-trifluoro-1-phenyl-2-(p-tolyl)butan-1-one (3ba)

^{1}H NMR (300 MHz, $CDCl_3$)

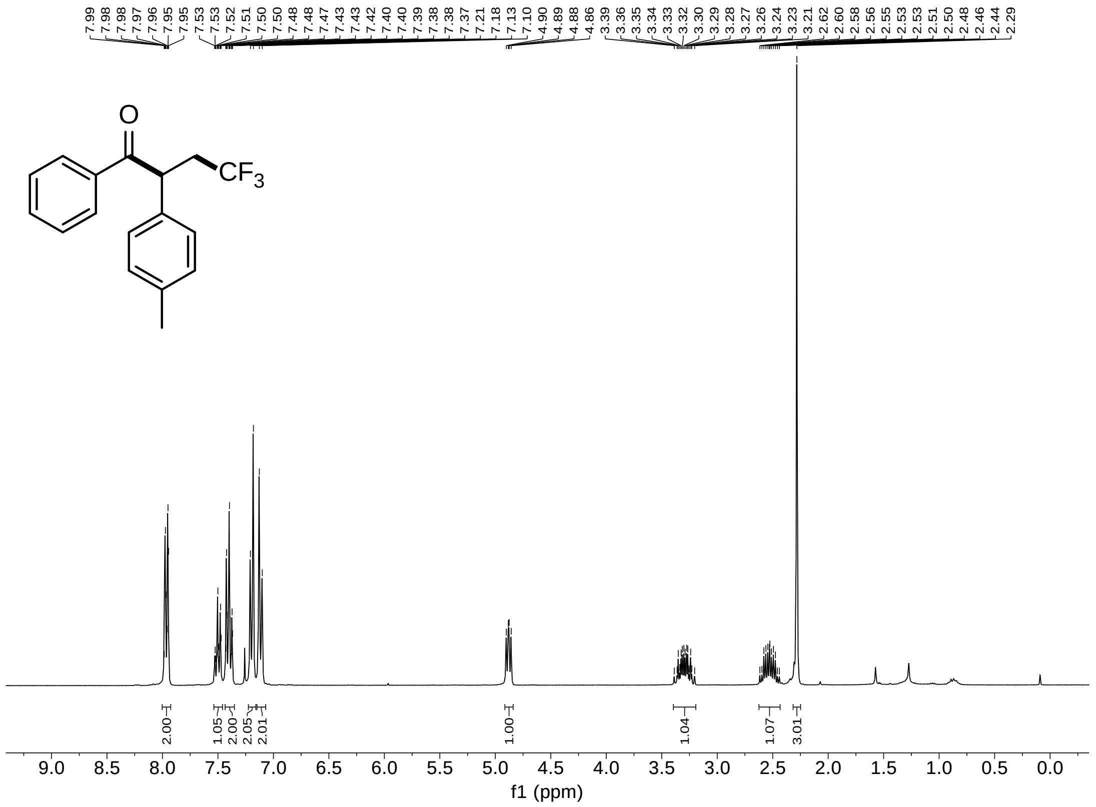

^{13}C NMR (75 MHz, $CDCl_3$)

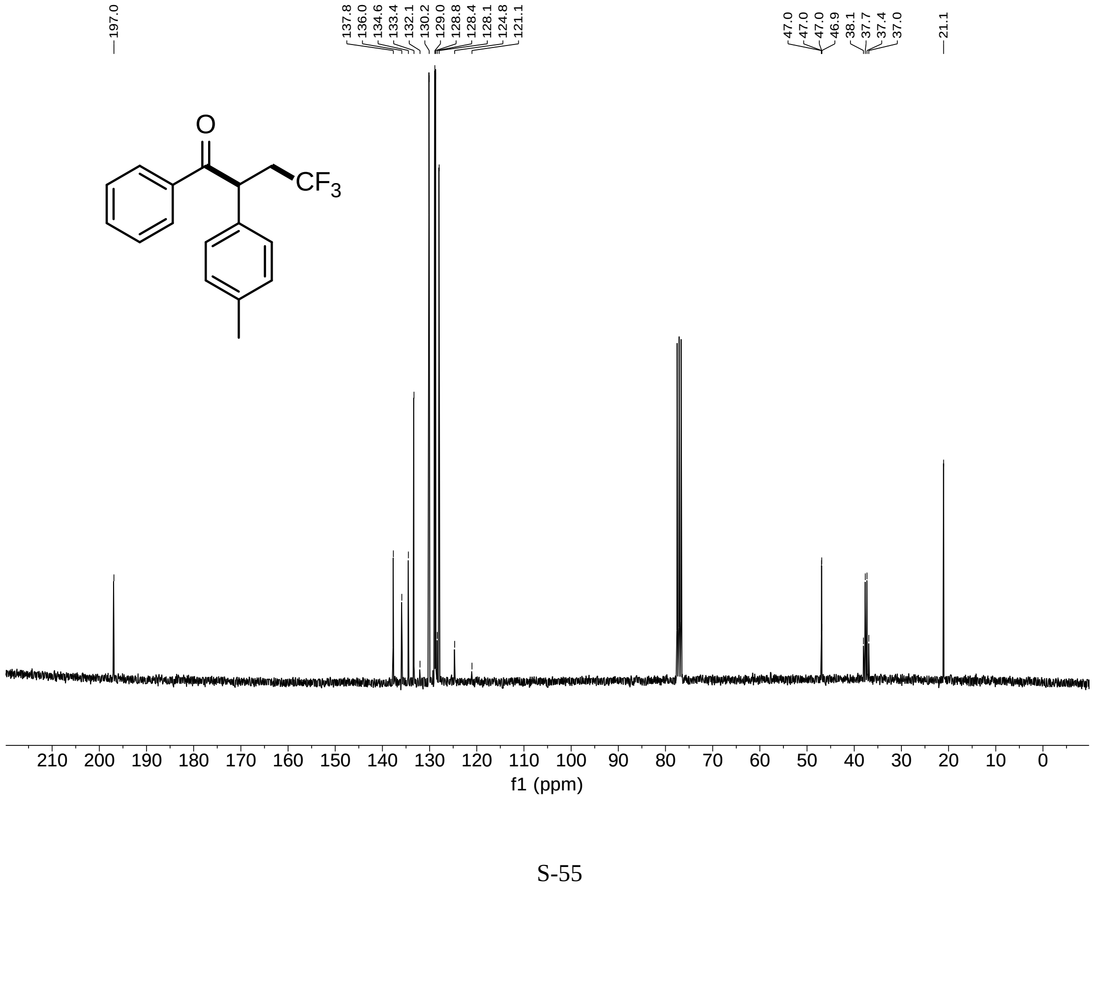

$^{19}F\ \{^{1}H\}$ NMR (282 MHz, $CDCl_3$)

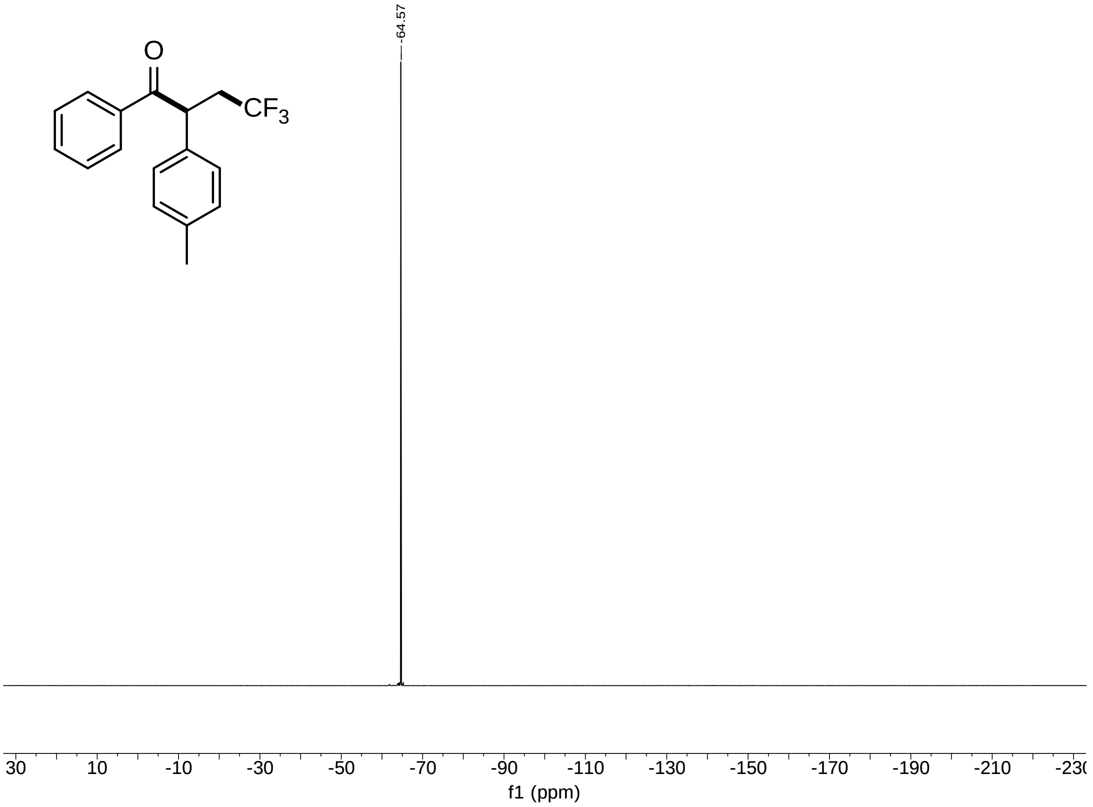

4,4,4-trifluoro-1-phenyl-2-(m-tolyl)butan-1-one (3ca)

^{1}H NMR (300 MHz, $CDCl_3$)

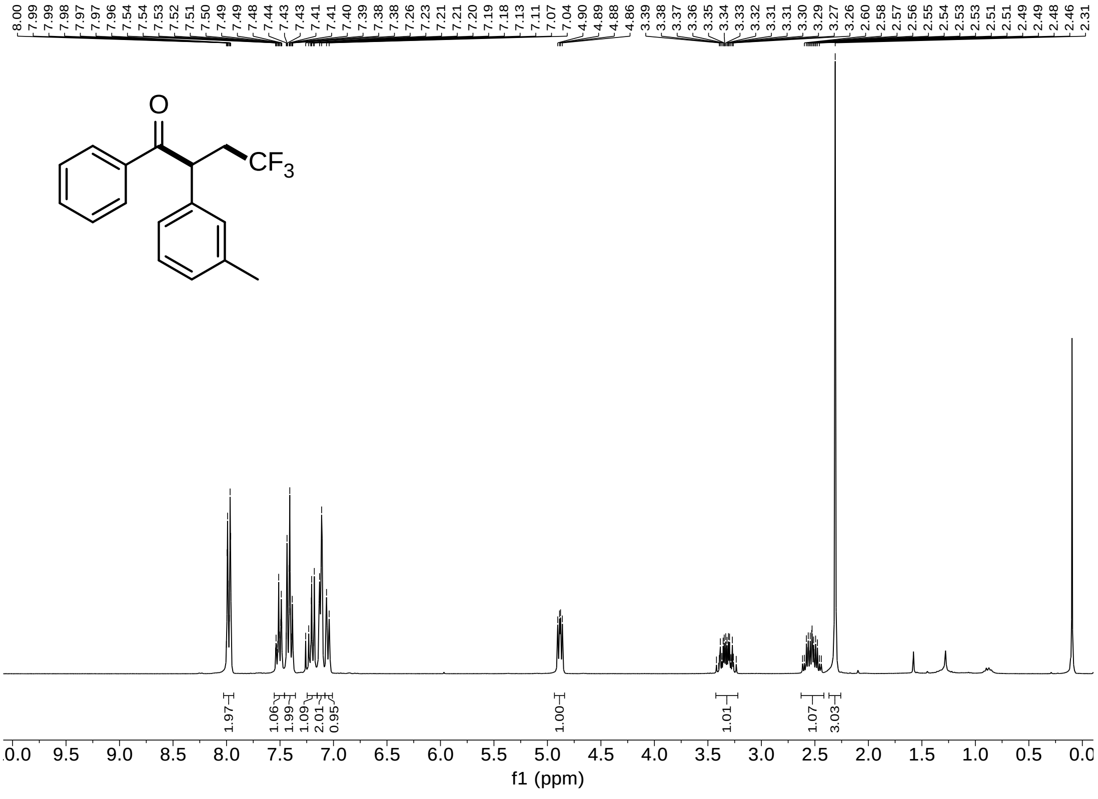

^{13}C NMR (75 MHz, $CDCl_3$)

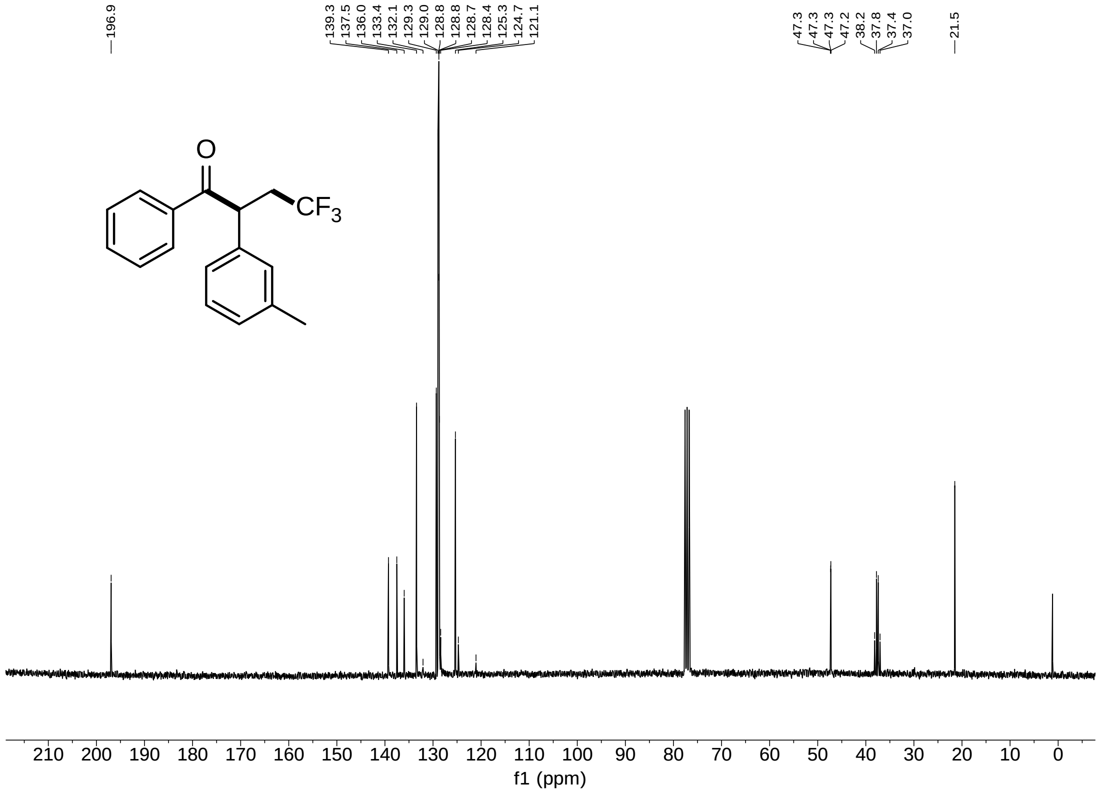

^{19}F {^{1}H} NMR (282 MHz, $CDCl_3$)

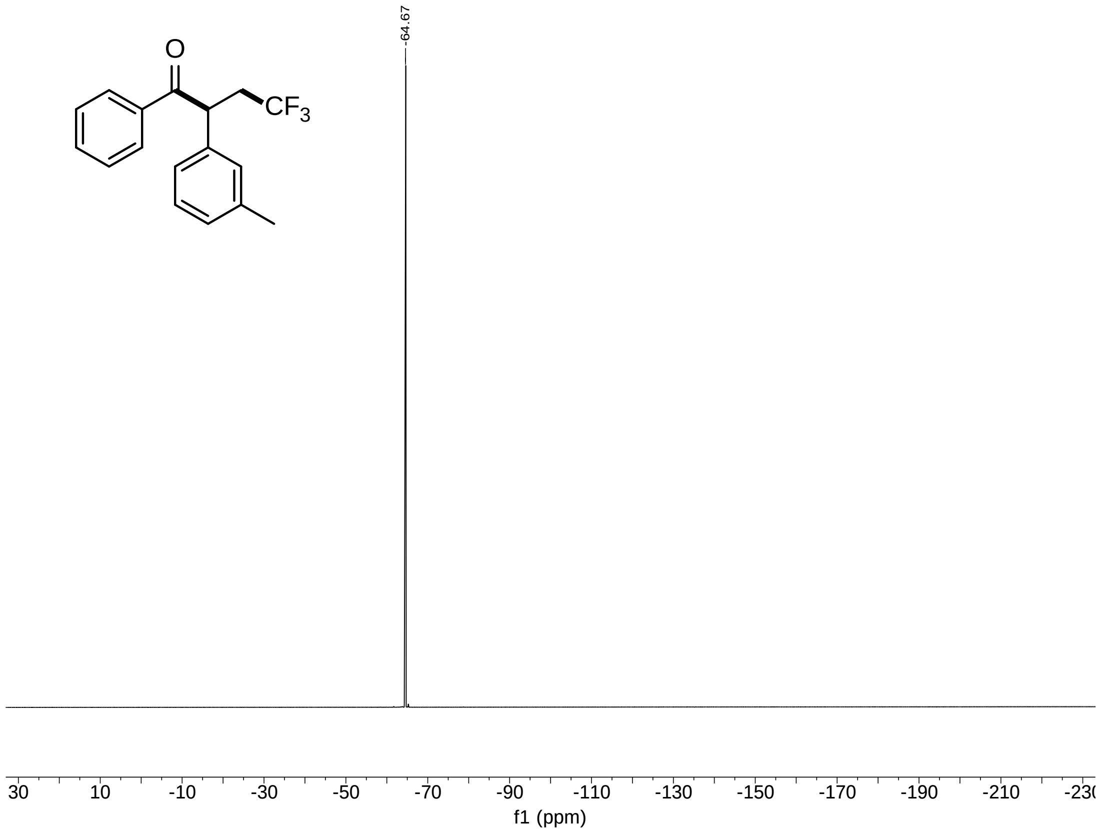

4,4,4-trifluoro-1-phenyl-2-(o-tolyl)butan-1-one (3da)

^{1}H NMR (300 MHz, $CDCl_3$)

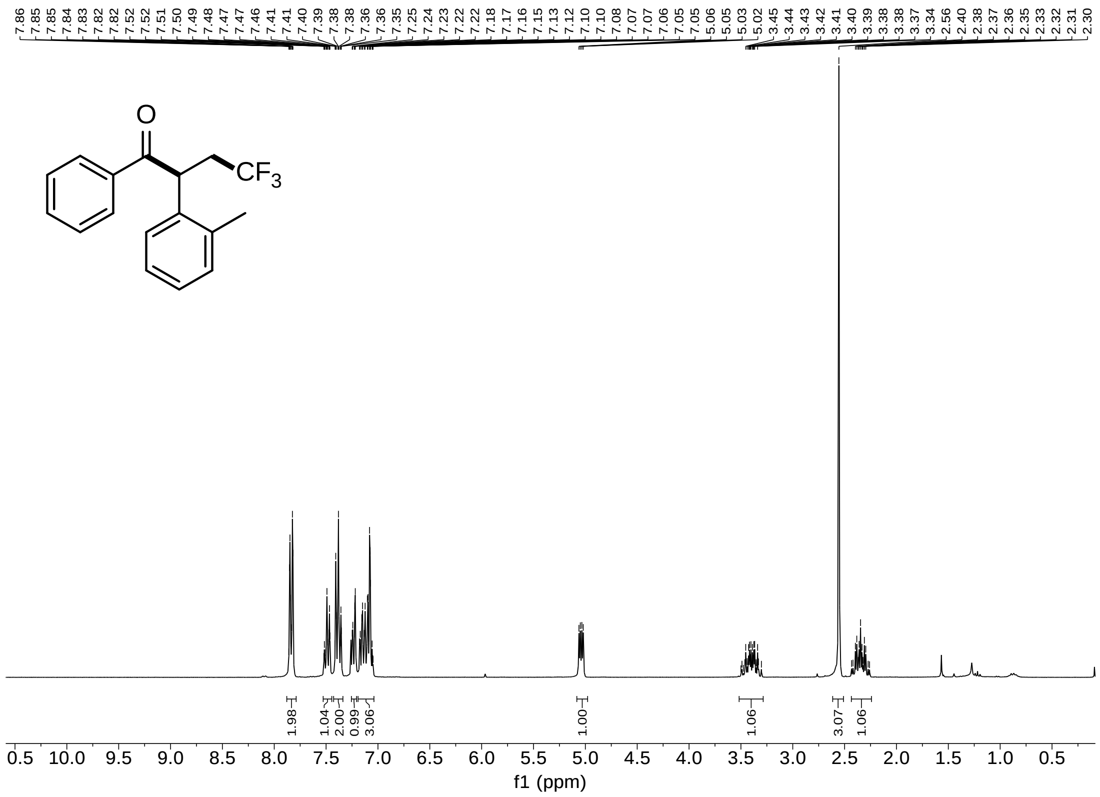

^{13}C NMR (75 MHz, $CDCl_3$)

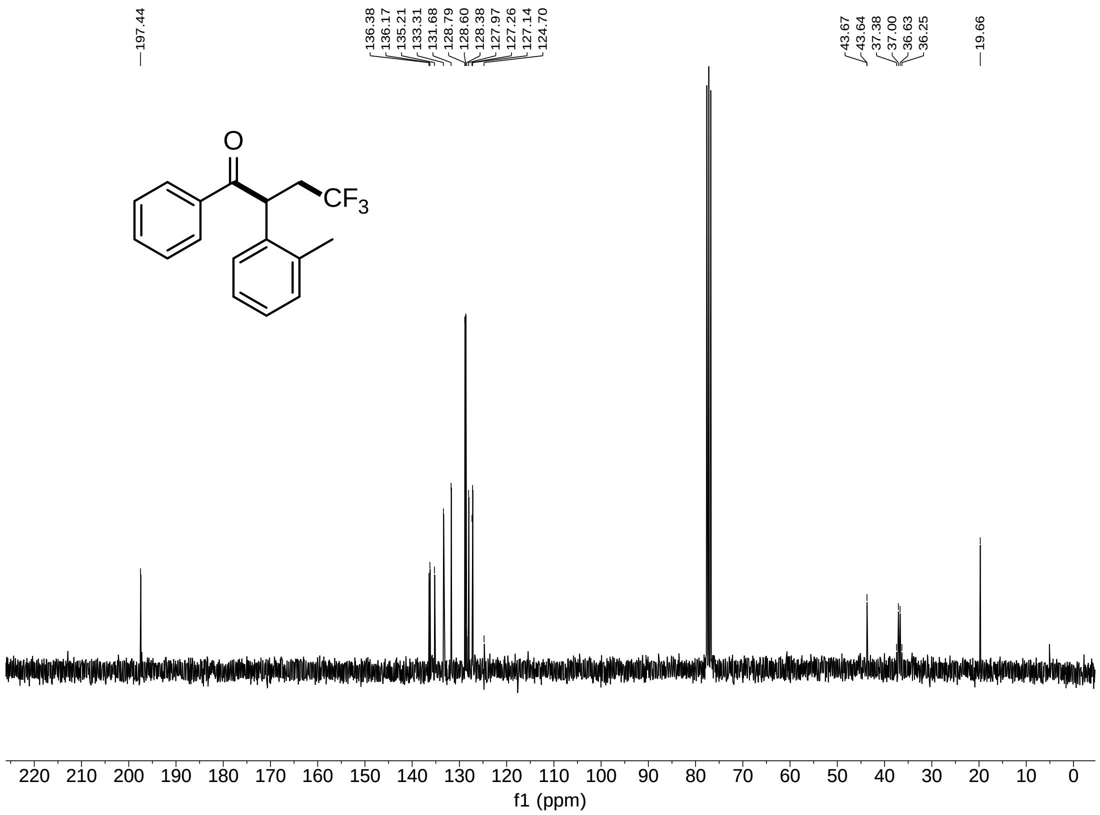

$^{19}F\{^{1}H\}$ NMR (282 MHz, $CDCl_3$)

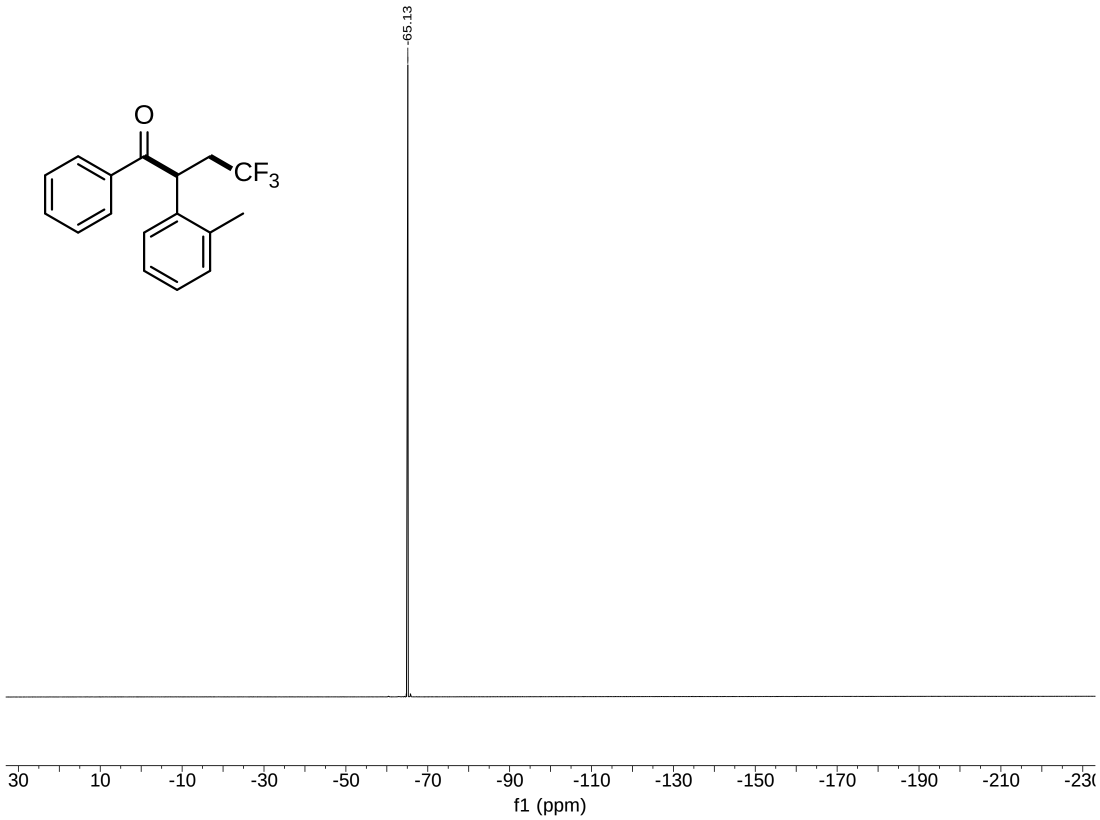

4,4,4-trifluoro-2-(4-methoxyphenyl)-1-phenylbutan-1-one (3ea)

^{1}H NMR (300 MHz, $CDCl_3$)

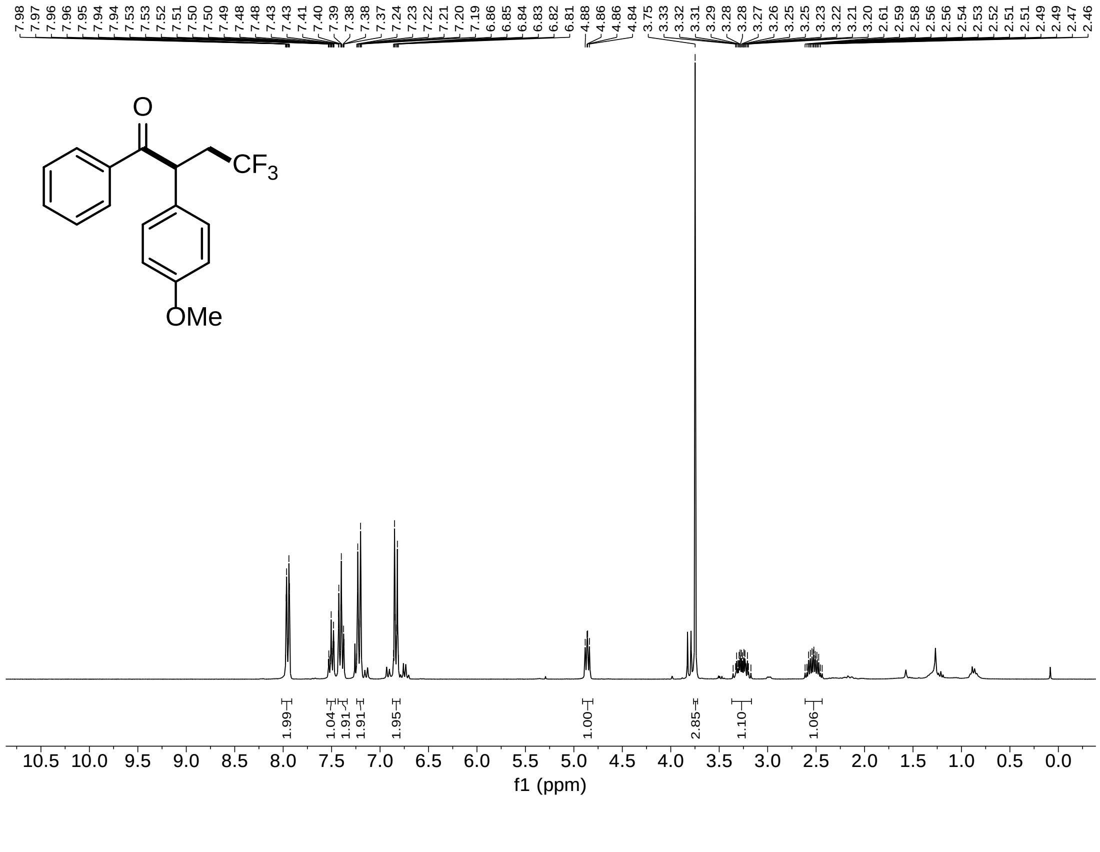

^{13}C NMR (75 MHz, CDCl$_3$)

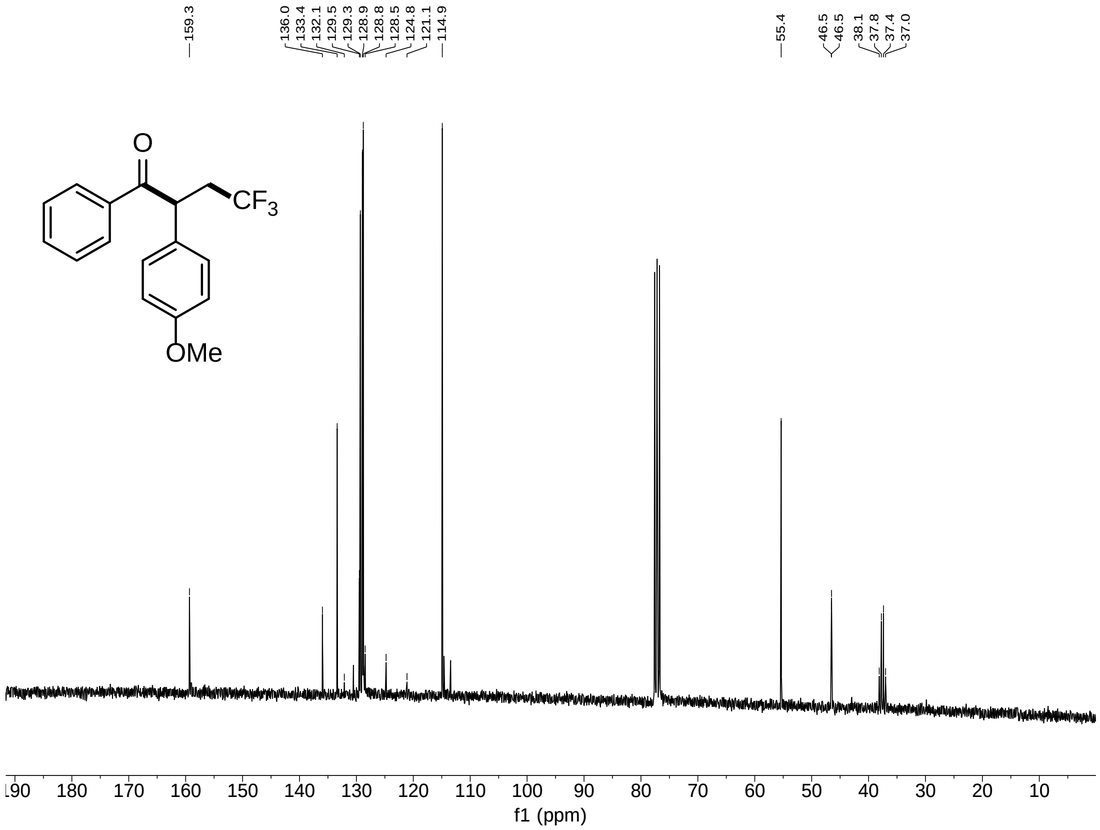

^{19}F {^{1}H} NMR (282 MHz, CDCl$_3$)

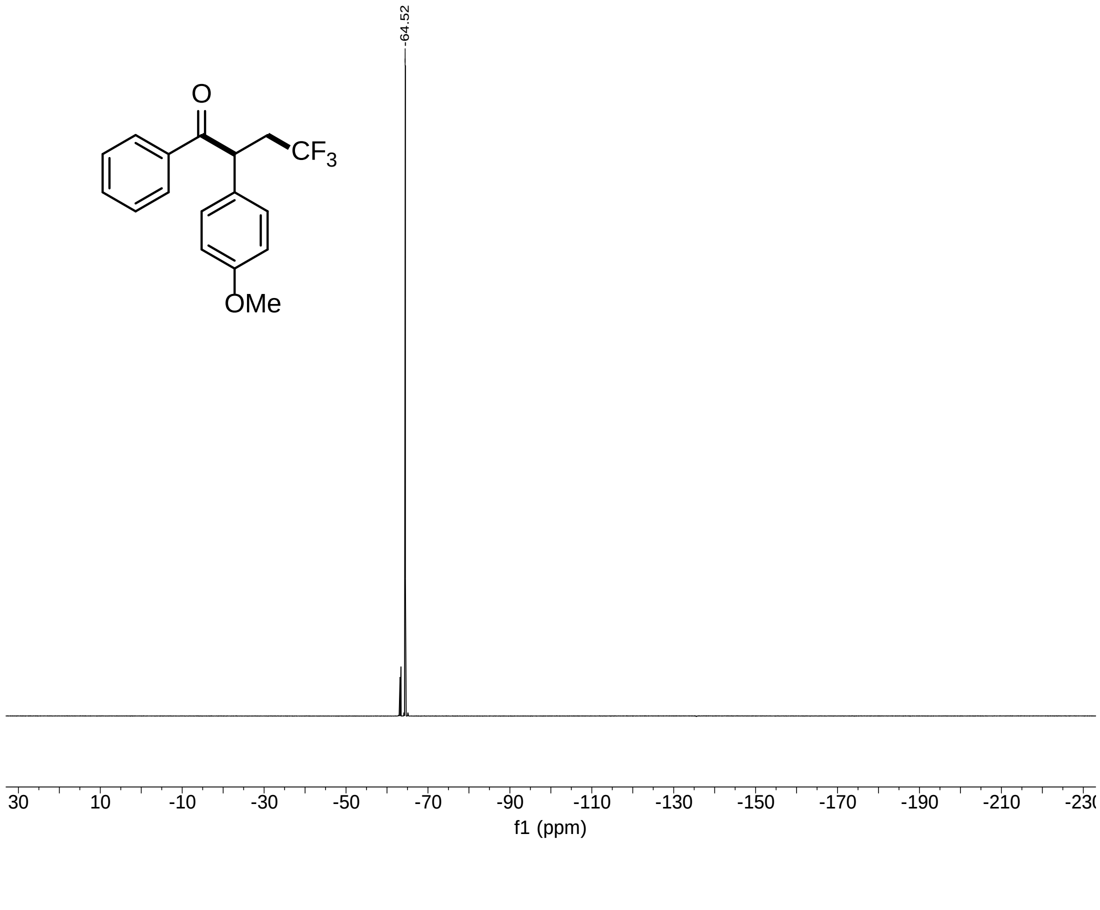

2-(4-(chloromethyl)phenyl)-4,4,4-trifluoro-1-phenylbutan-1-one (3fa)

^{1}H NMR (300 MHz, $CDCl_3$)

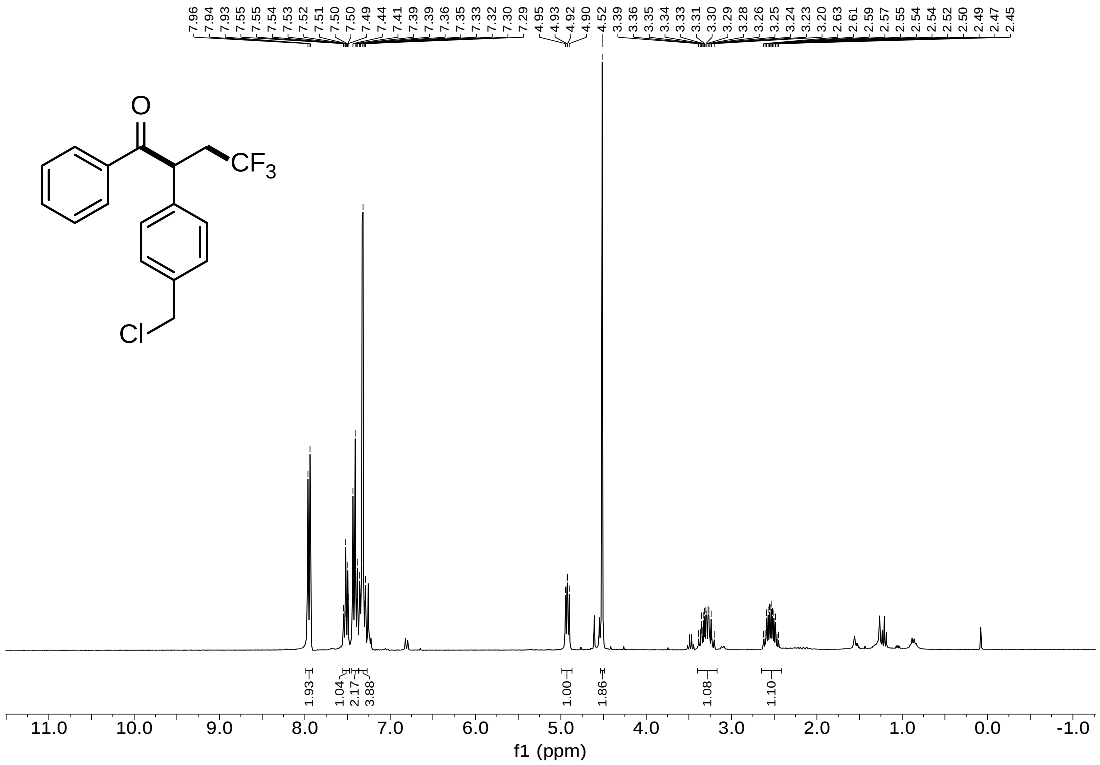

^{13}C NMR (75 MHz, $CDCl_3$)

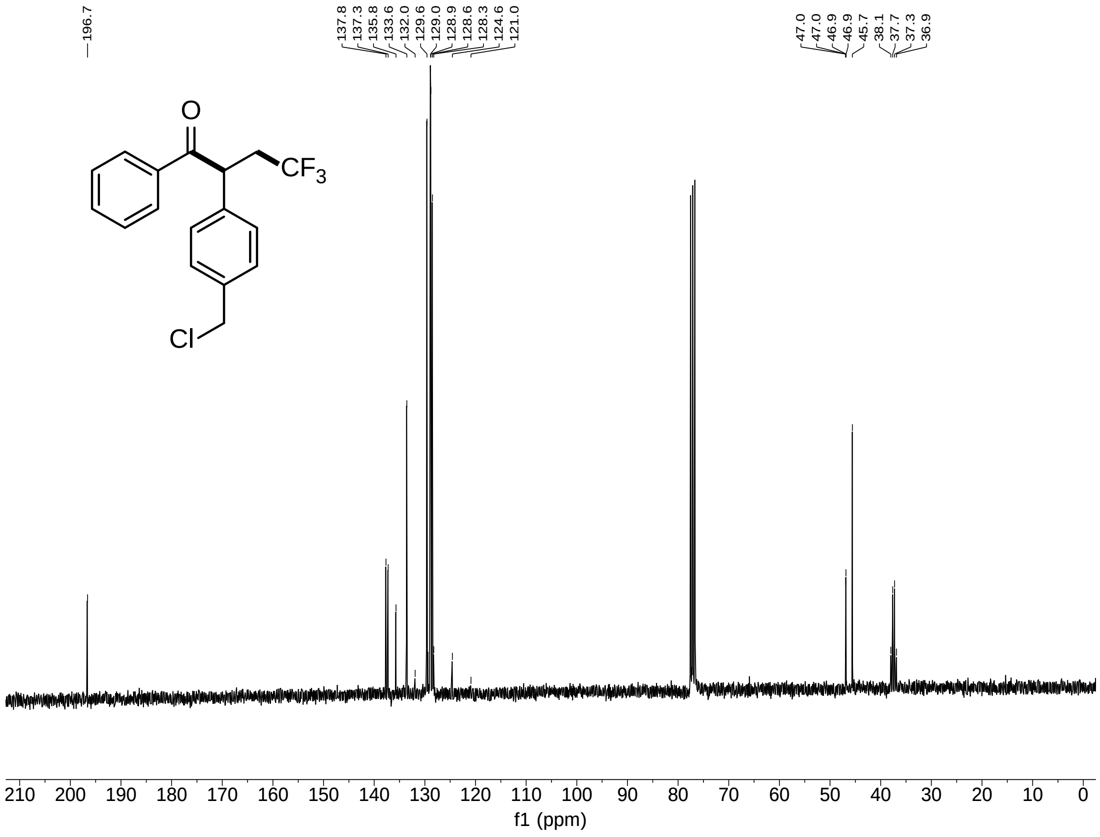

$^{19}F\{^{1}H\}$ NMR (282 MHz, $CDCl_3$)

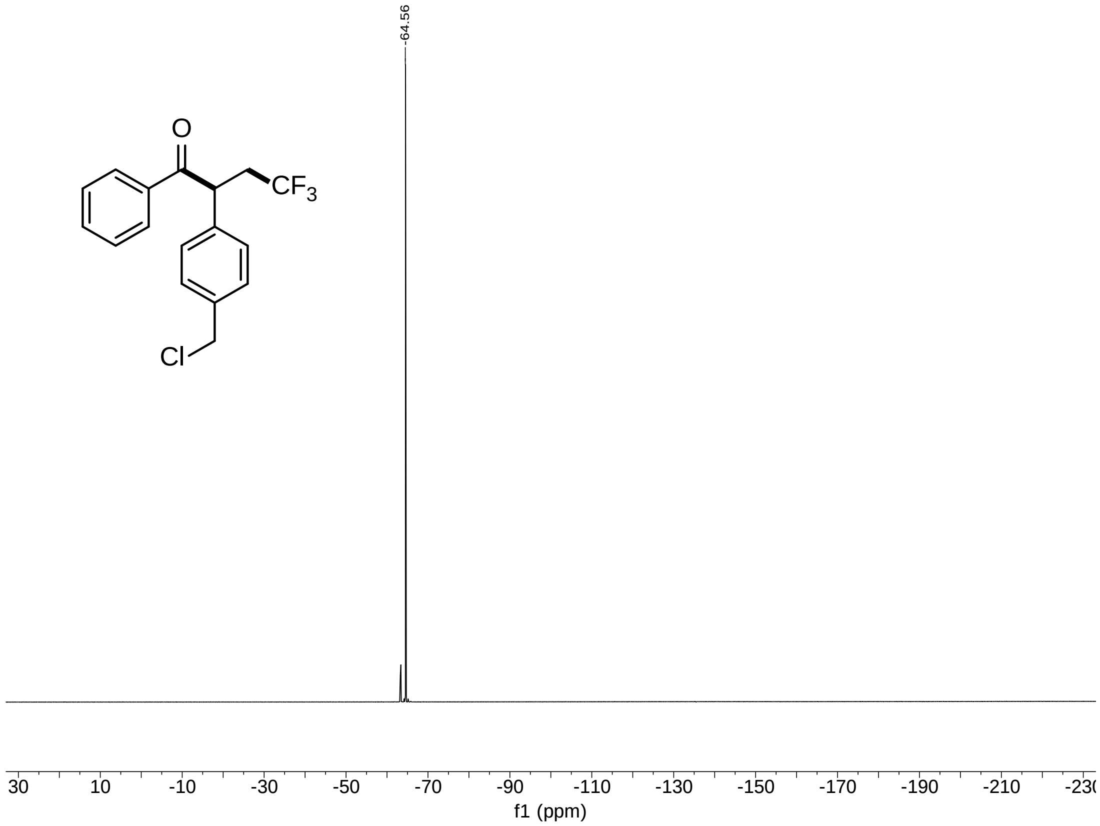

4,4,4-trifluoro-2-(4-fluorophenyl)-1-phenylbutan-1-one (3ga)

^{1}H NMR (300 MHz, $CDCl_3$)

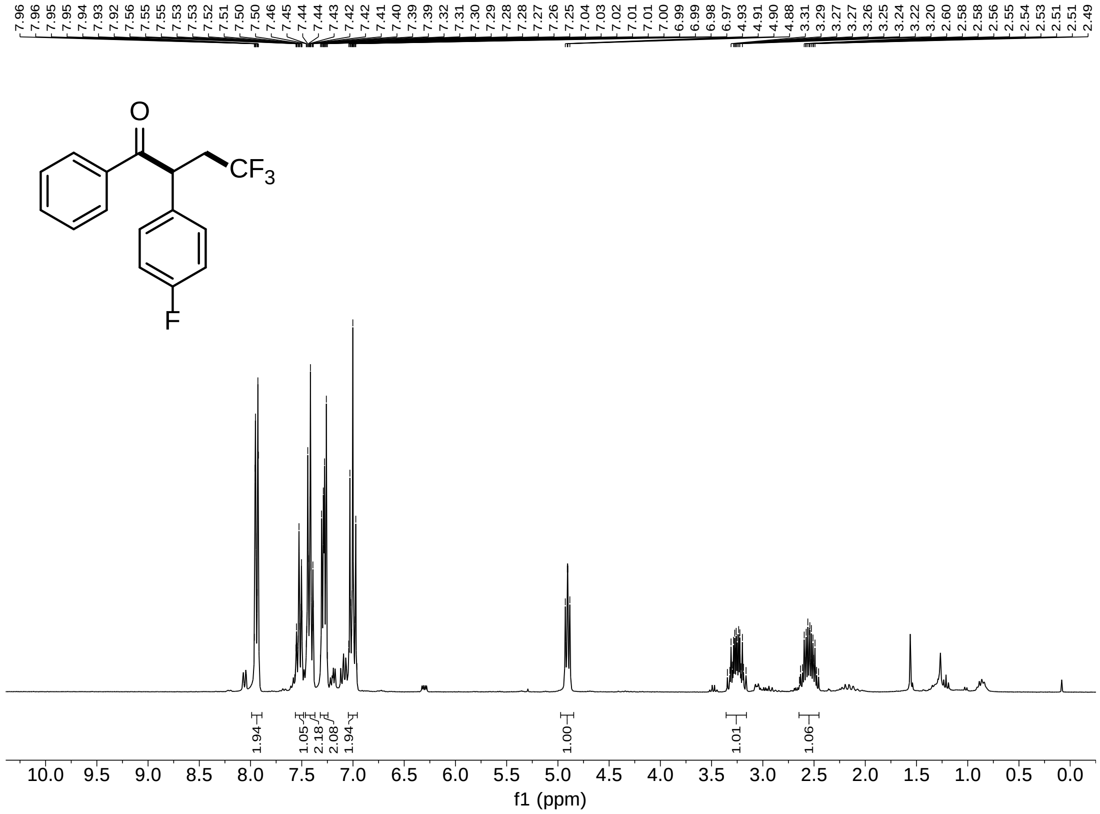

^{13}C NMR (75 MHz, $CDCl_3$)

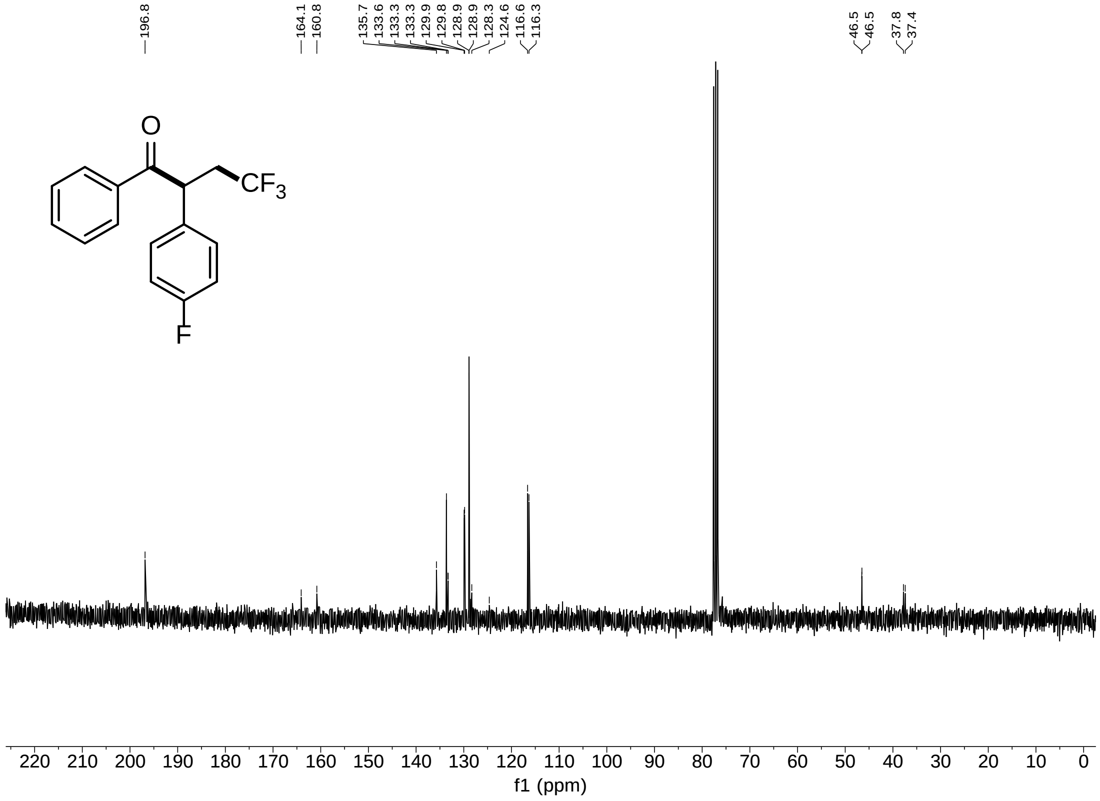

^{19}F {^{1}H} NMR (282 MHz, $CDCl_3$)

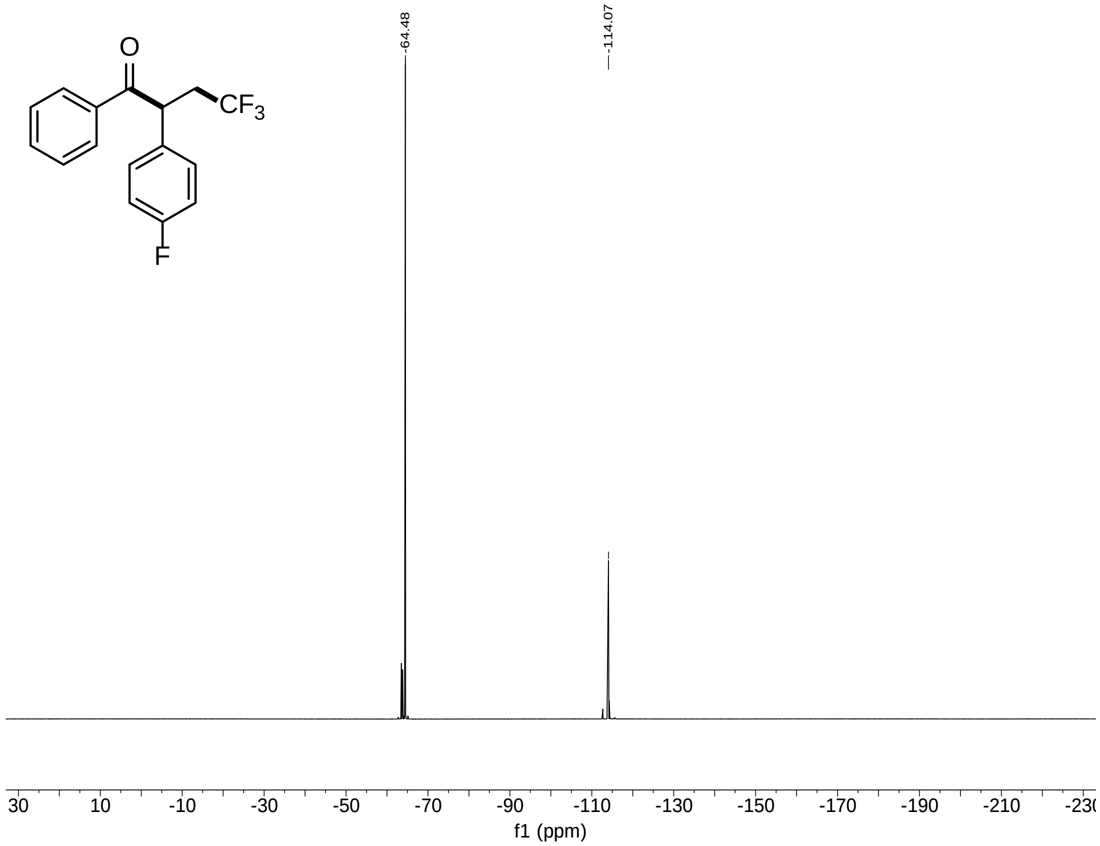

2-(4-chlorophenyl)-4,4,4-trifluoro-1-phenylbutan-1-one (3ha)

^{1}H NMR (300 MHz, $CDCl_3$)

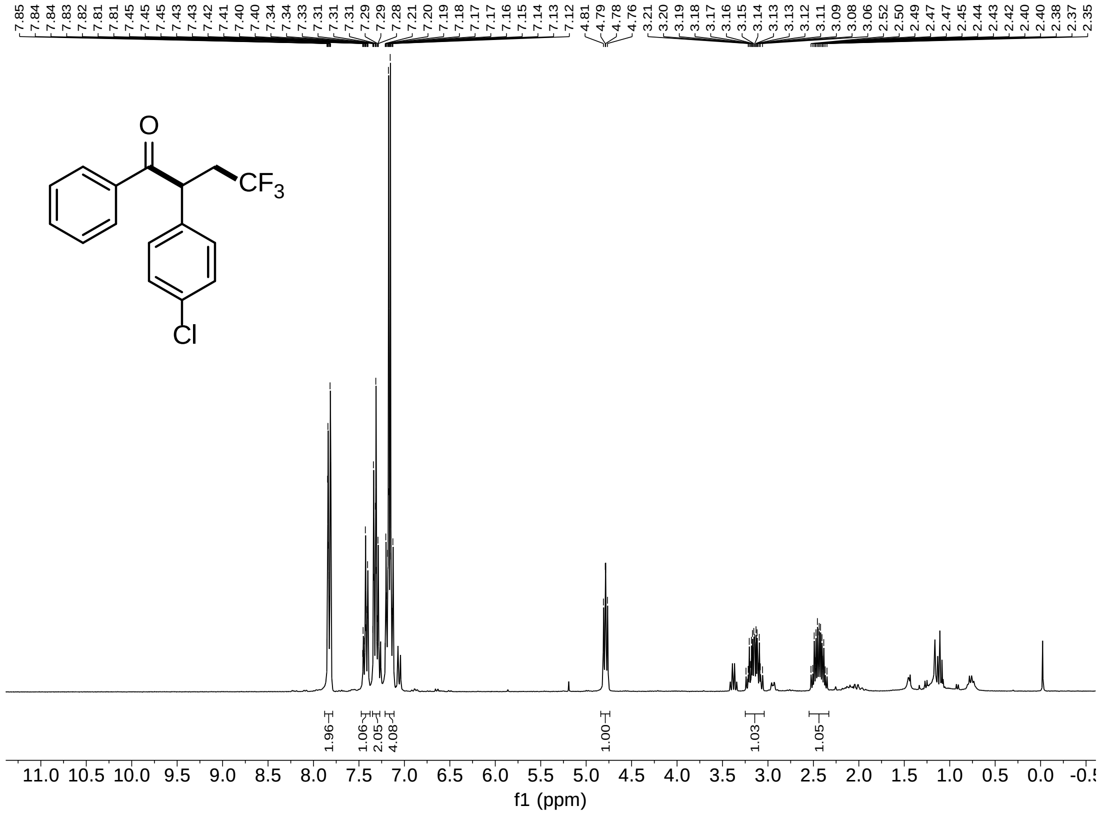

^{13}C NMR (75 MHz, $CDCl_3$)

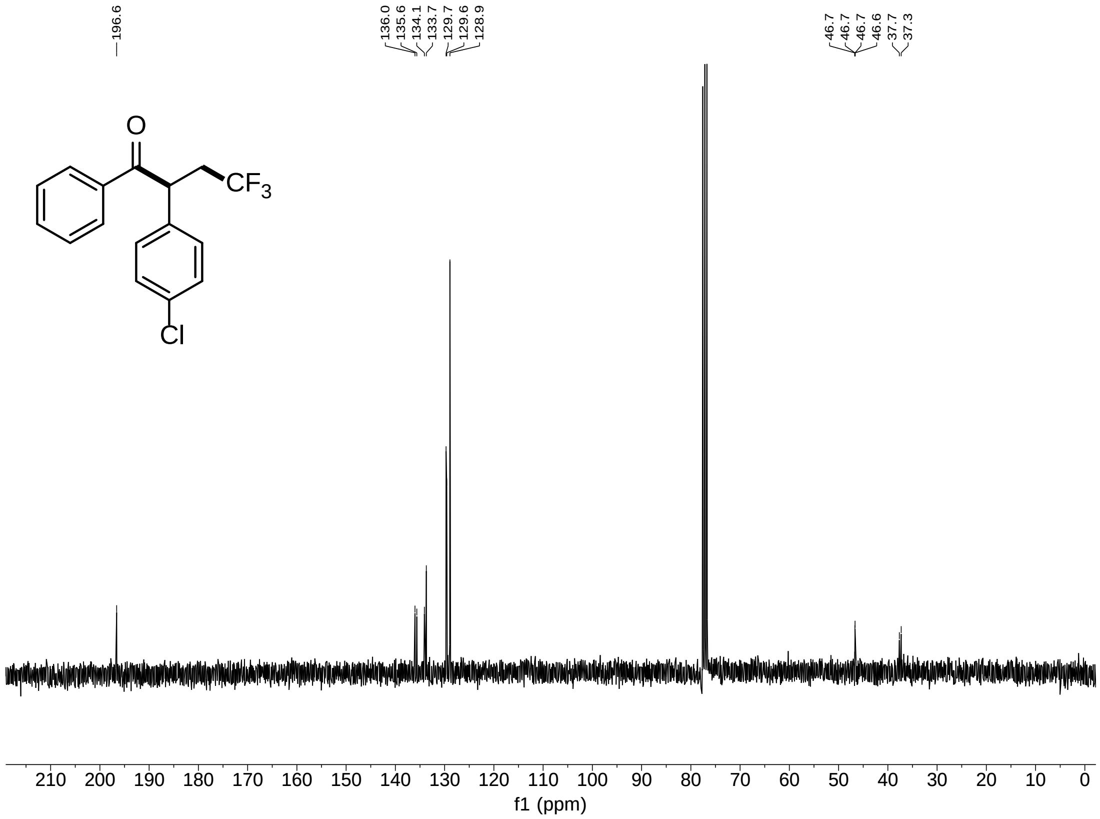

^{19}F {^{1}H} NMR (282 MHz, $CDCl_3$)

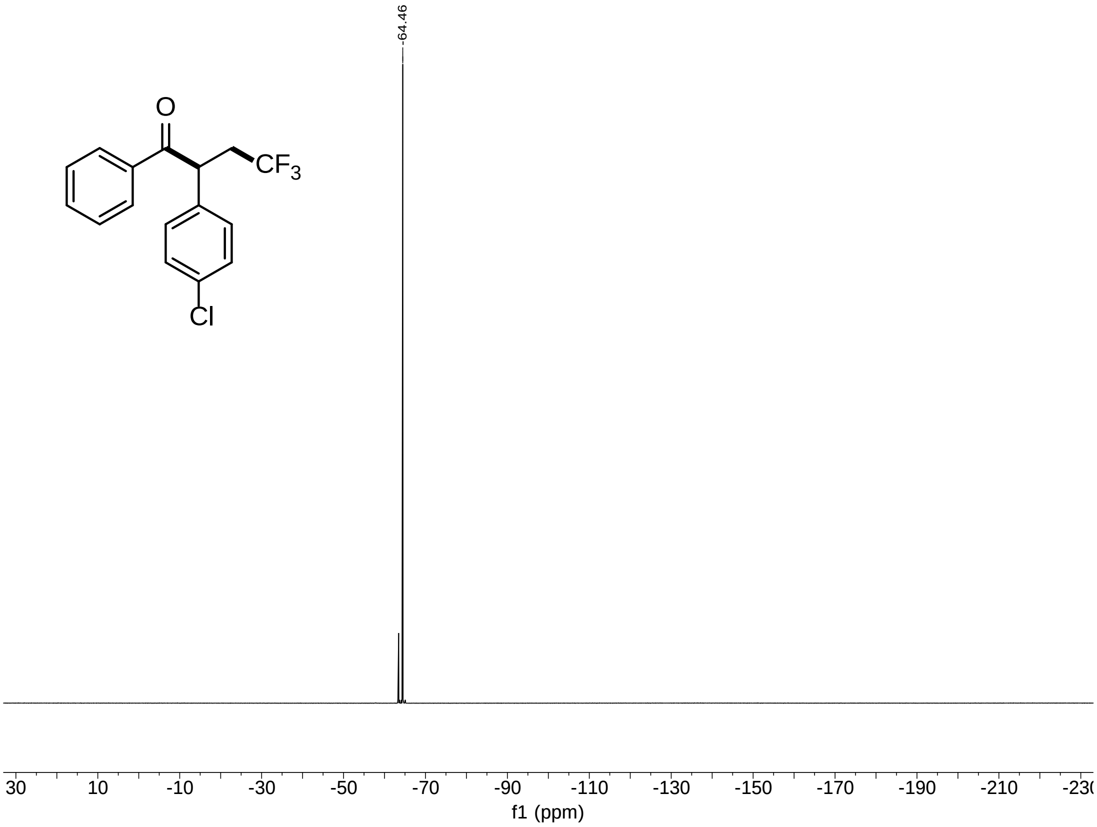

4,4,4-trifluoro-1-phenyl-2-(4-(trifluoromethyl)phenyl)butan-1-one (3ia)

^{1}H NMR (300 MHz, $CDCl_3$)

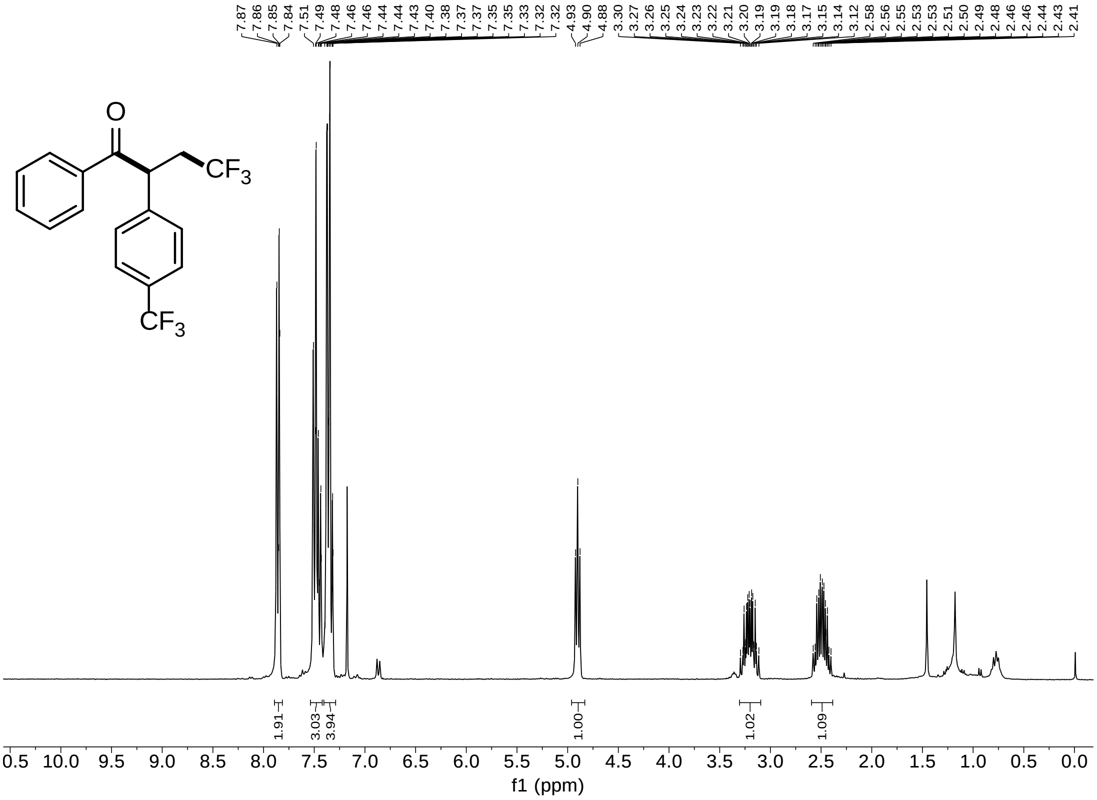

^{13}C {^{19}F} NMR (150 MHz, $CDCl_3$)

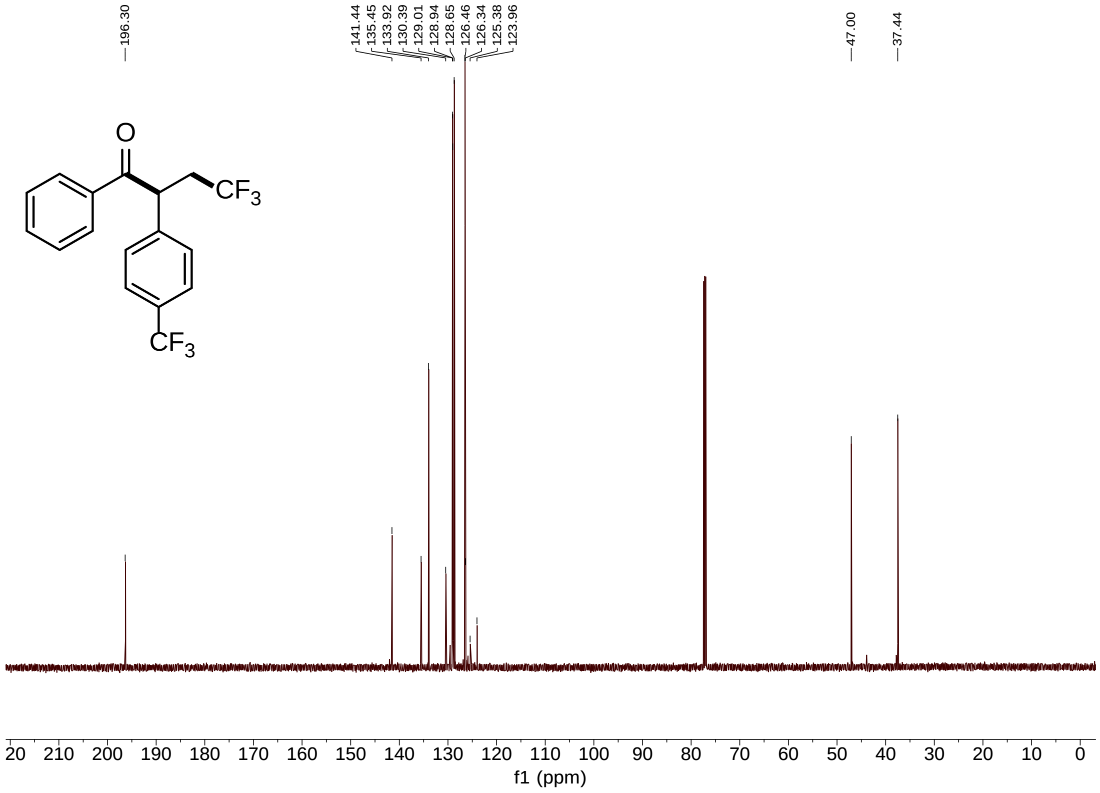

^{19}F {^{1}H} NMR (282 MHz, $CDCl_3$)

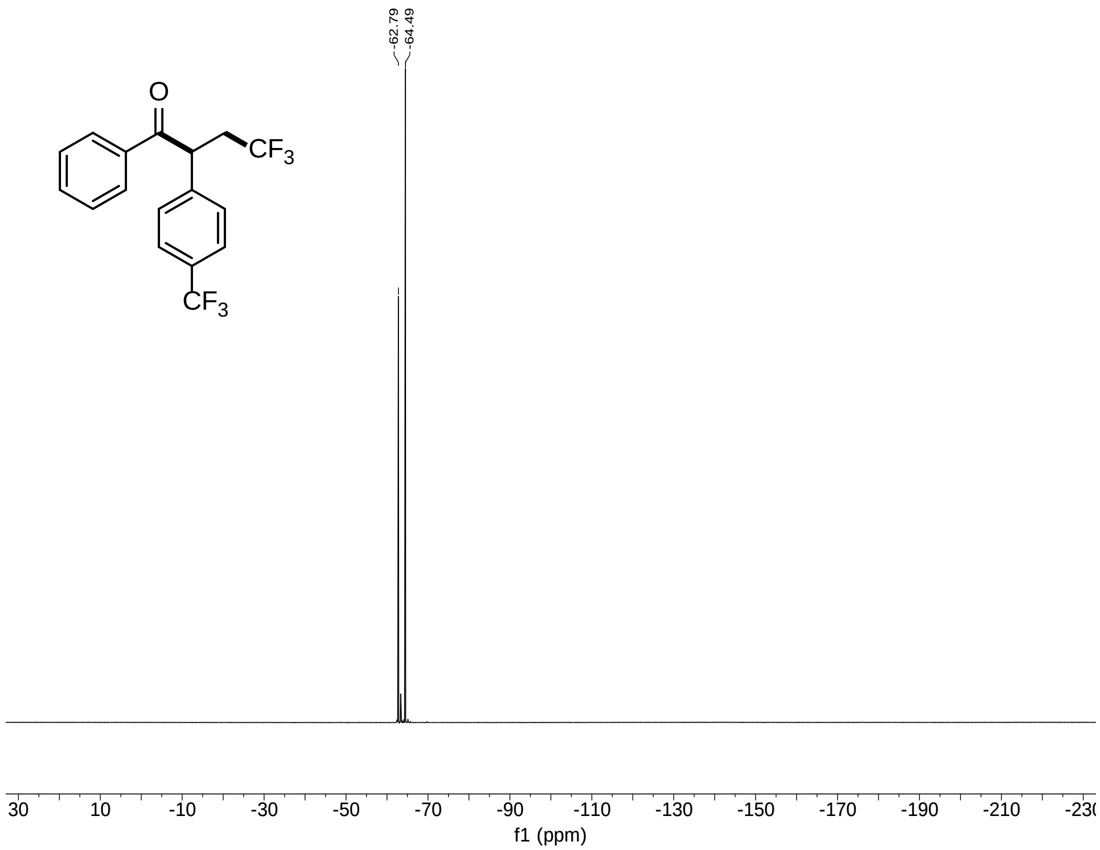

2-([1,1'-biphenyl]-4-yl)-4,4,4-trifluoro-1-phenylbutan-1-one (3ja)

^{1}H NMR (300 MHz, $CDCl_3$)

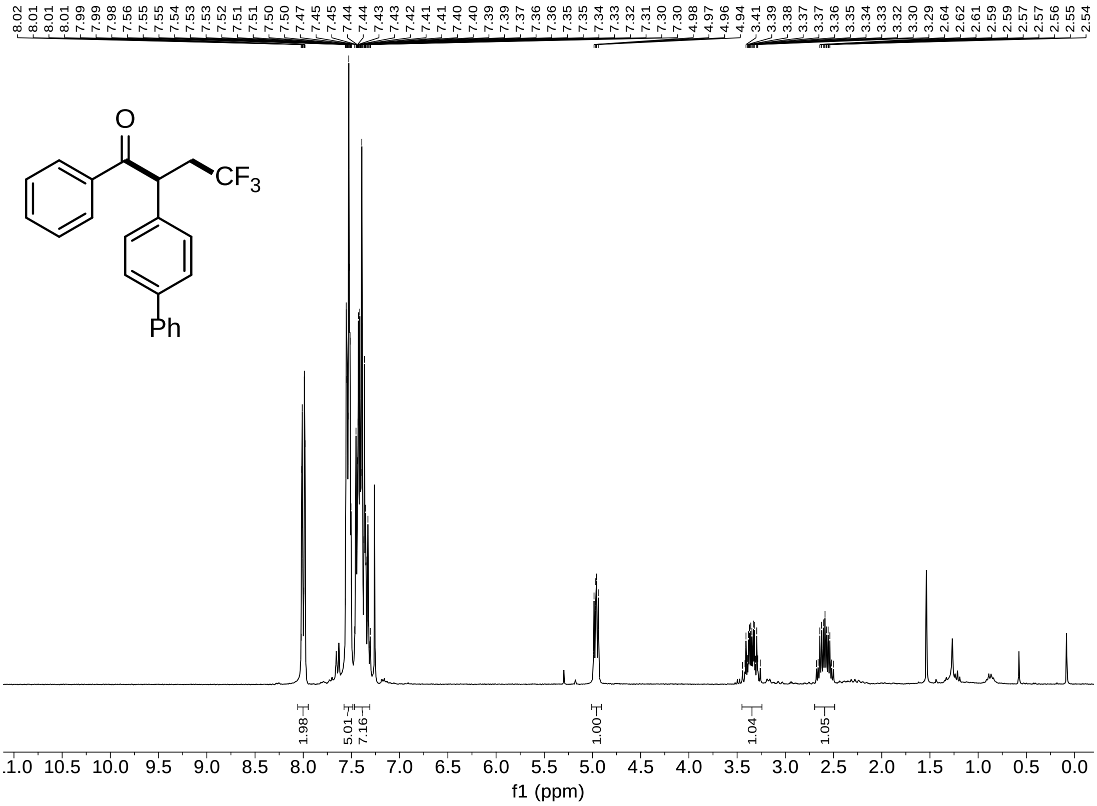

^{13}C NMR (75 MHz, $CDCl_3$)

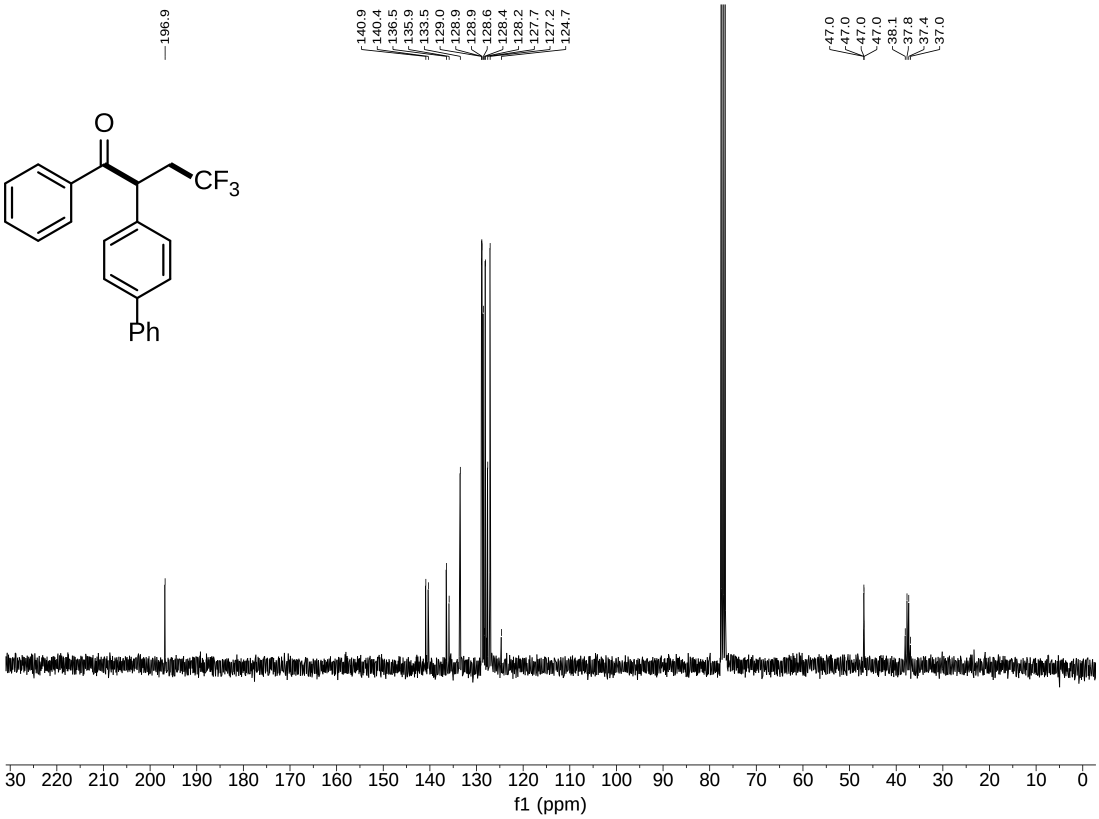

$^{19}F\ \{^{1}H\}$ NMR (282 MHz, $CDCl_3$)

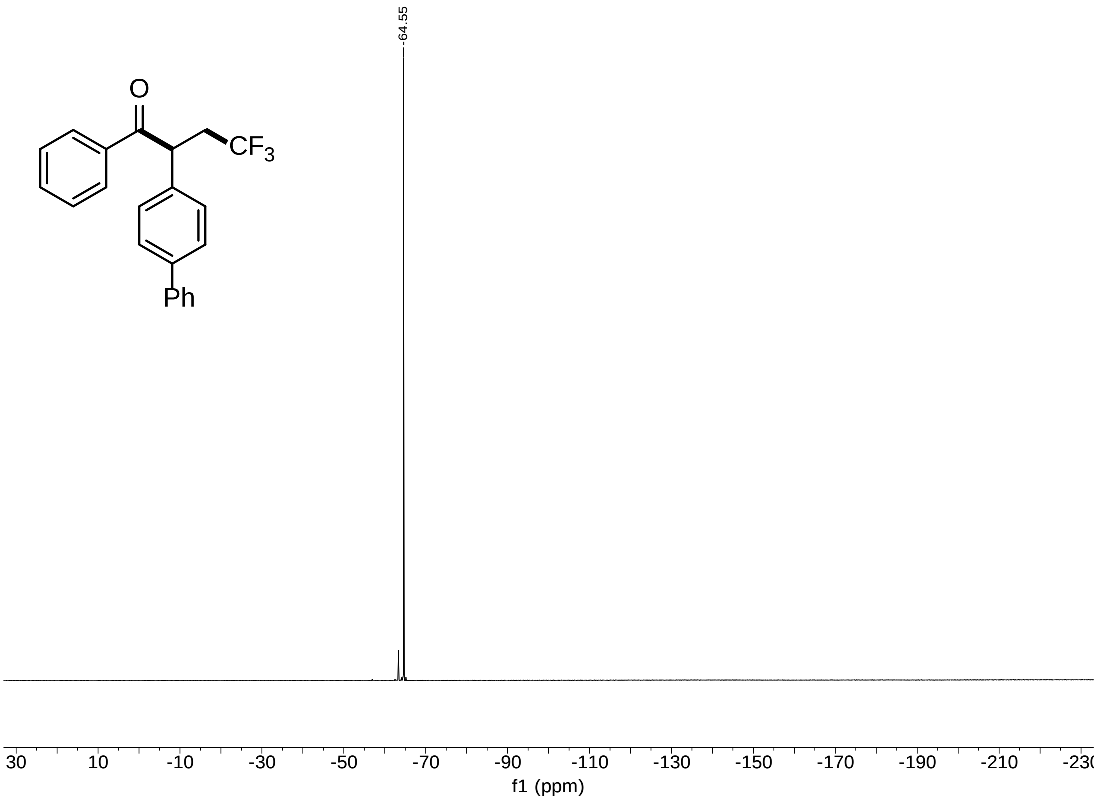

4,4,4-trifluoro-2-(naphthalen-2-yl)-1-phenylbutan-1-one (3ka)

^{1}H NMR (300 MHz, $CDCl_3$)

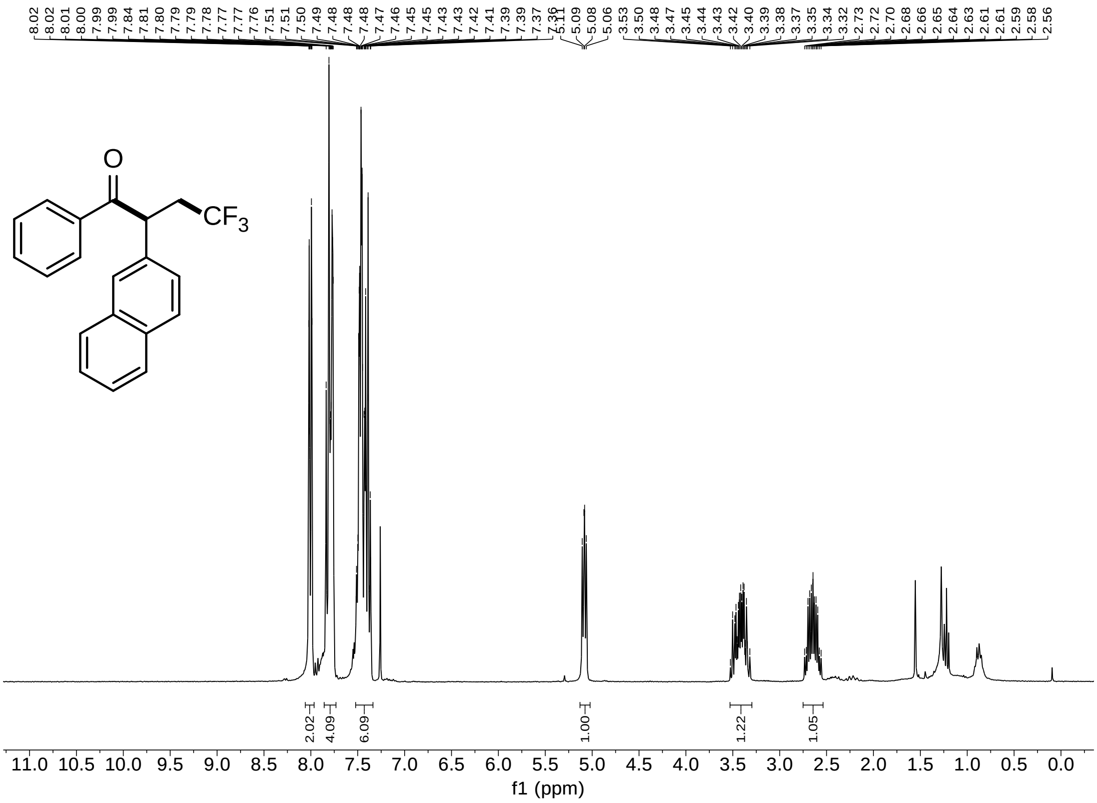

^{13}C NMR (75 MHz, $CDCl_3$)

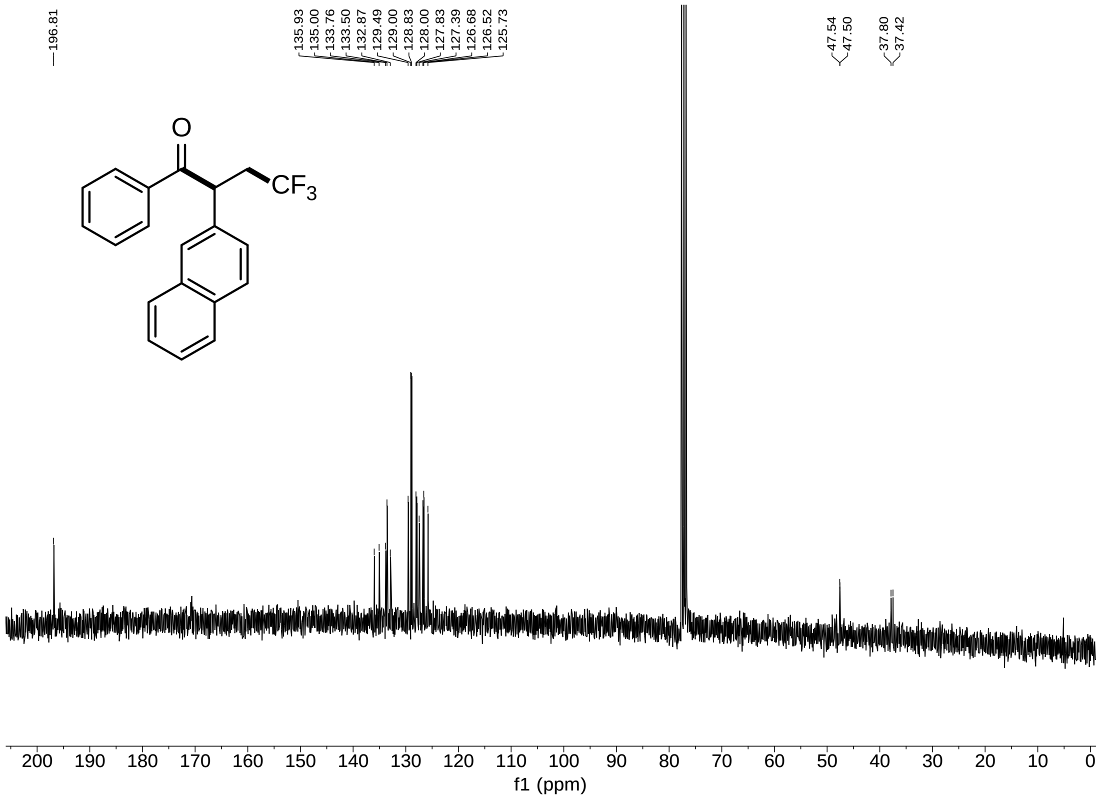

^{19}F {^{1}H} NMR (282 MHz, $CDCl_3$)

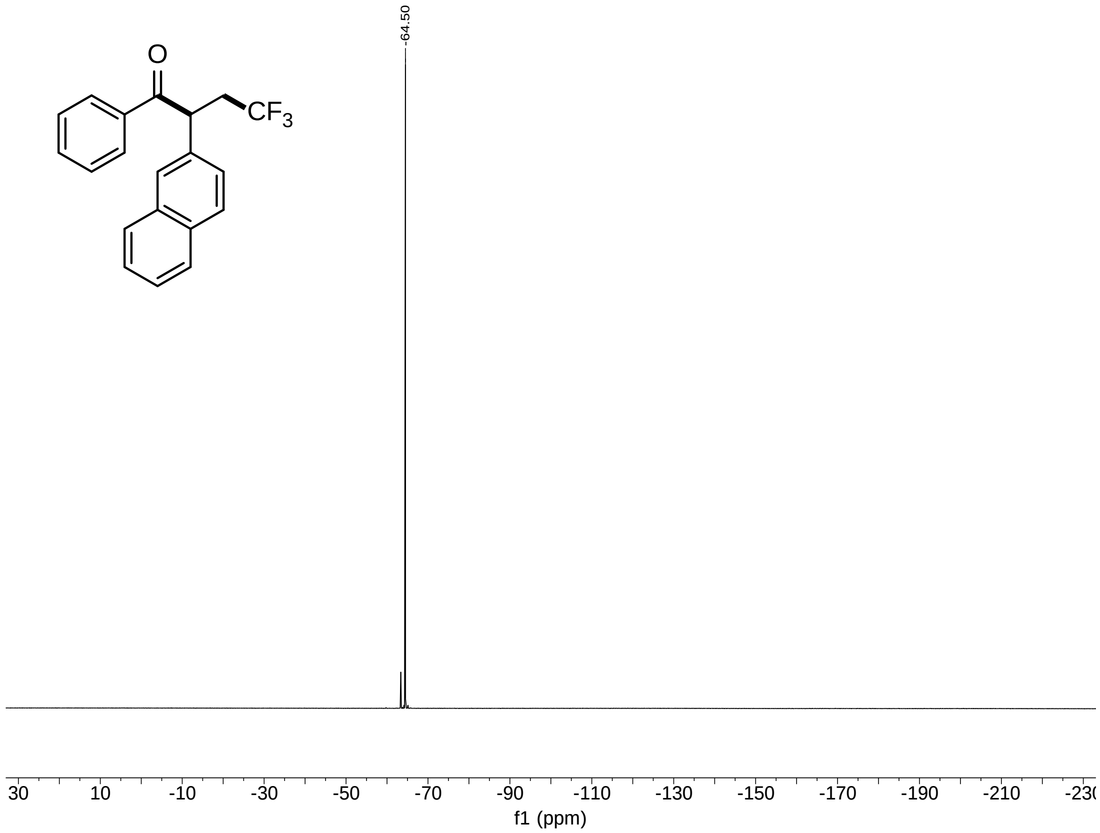

4,4,4-trifluoro-1-phenyl-2-(pyridin-2-yl)butan-1-one (3la)

^{1}H NMR (300 MHz, $CDCl_3$)

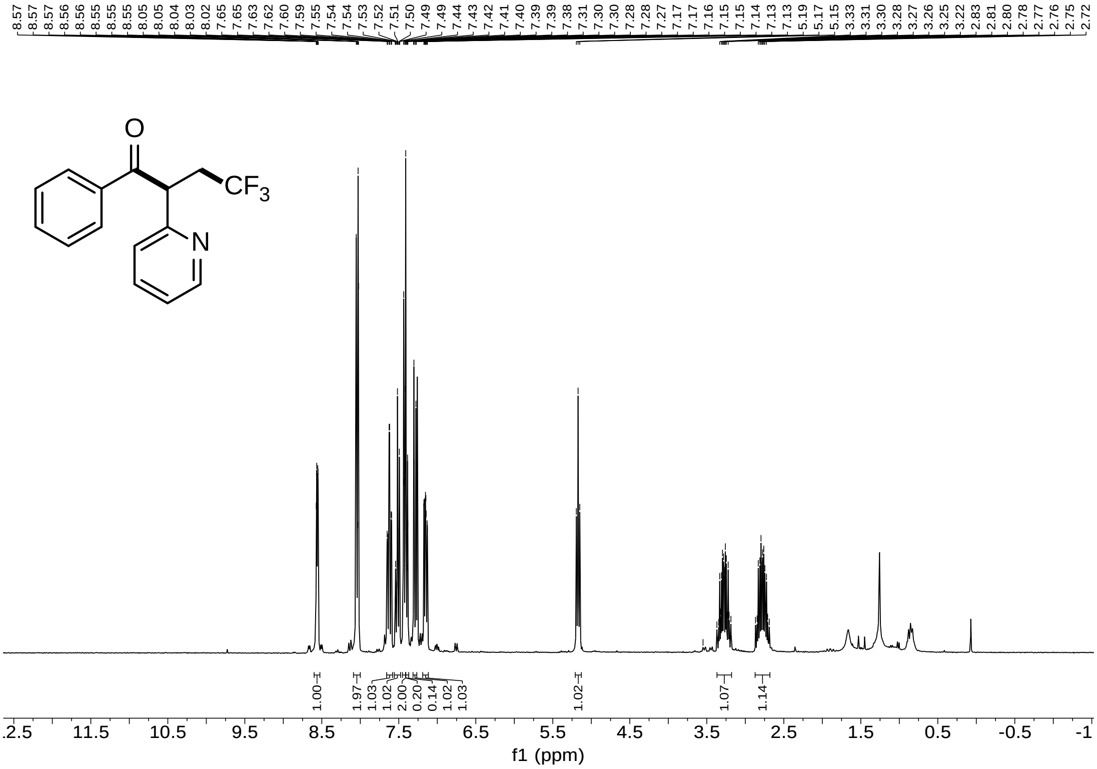

^{13}C NMR (75 MHz, $CDCl_3$)

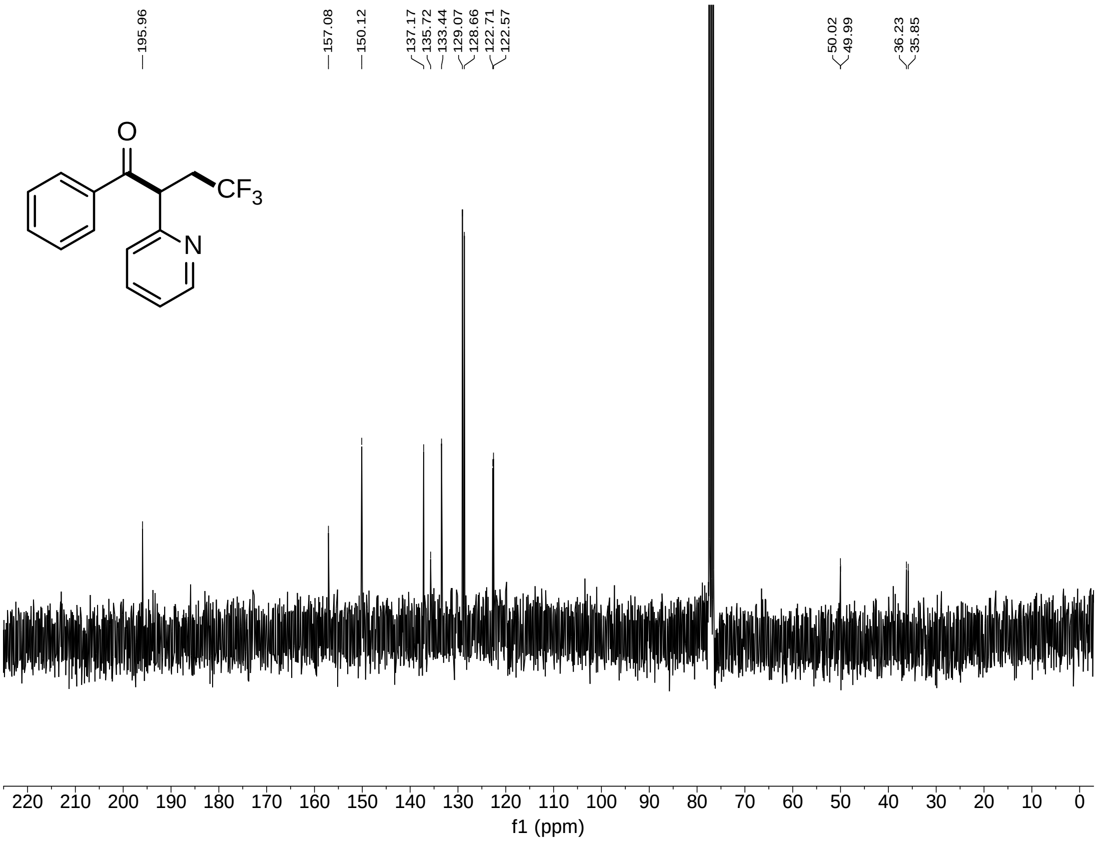

^{19}F {^{1}H} NMR (282 MHz, $CDCl_3$)

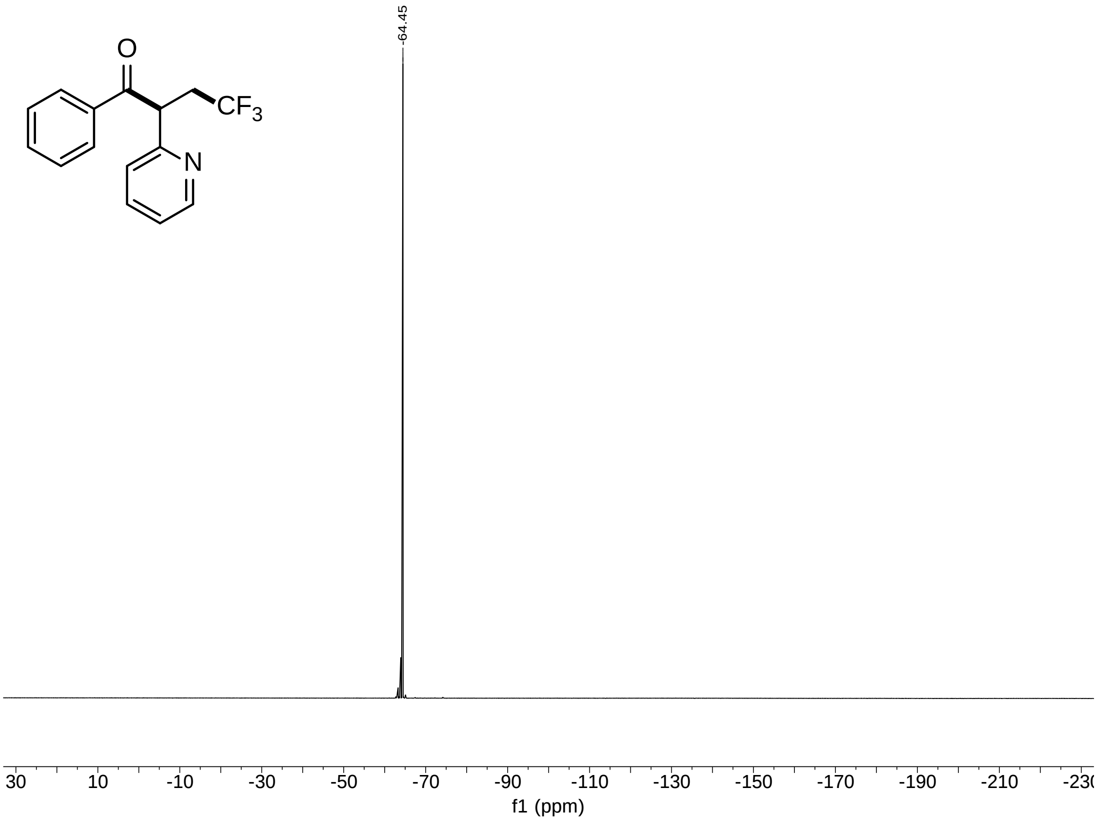

2-benzyl-4,4,4-trifluoro-1-phenylbutan-1-one (3ma)

^{1}H NMR (300 MHz, $CDCl_3$)

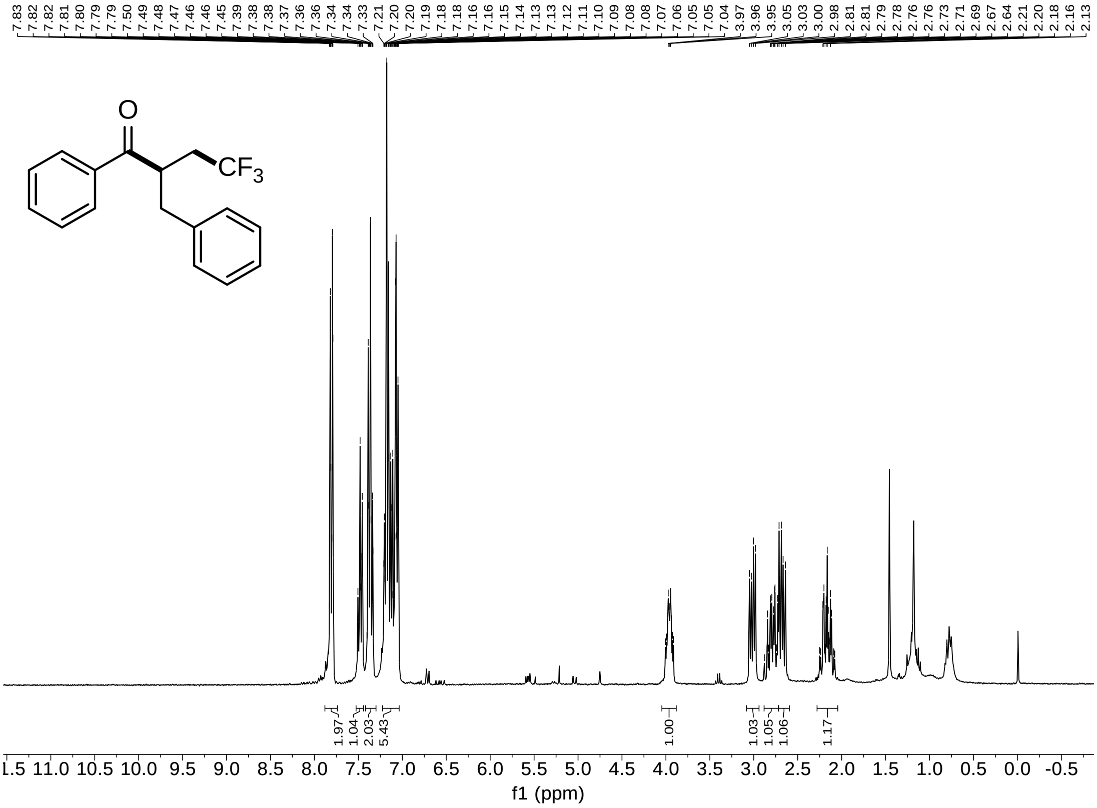

^{13}C NMR (75 MHz, $CDCl_3$)

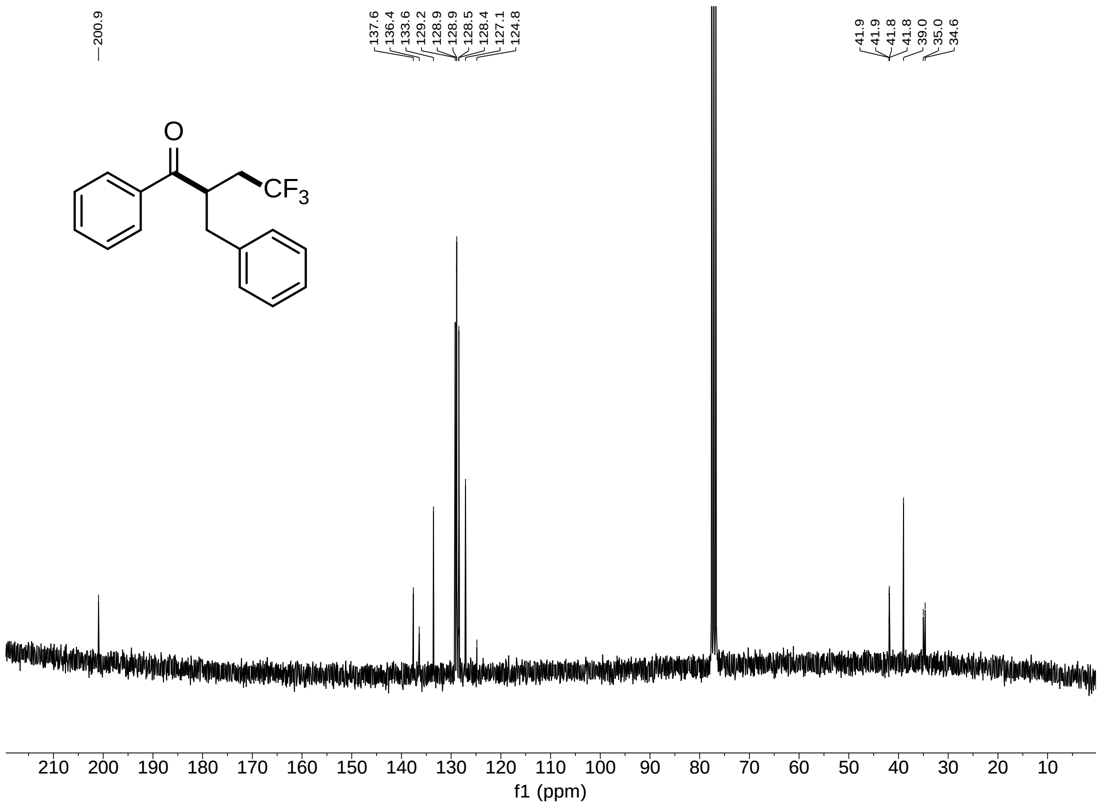

^{19}F {^1H} NMR (282 MHz, $CDCl_3$)

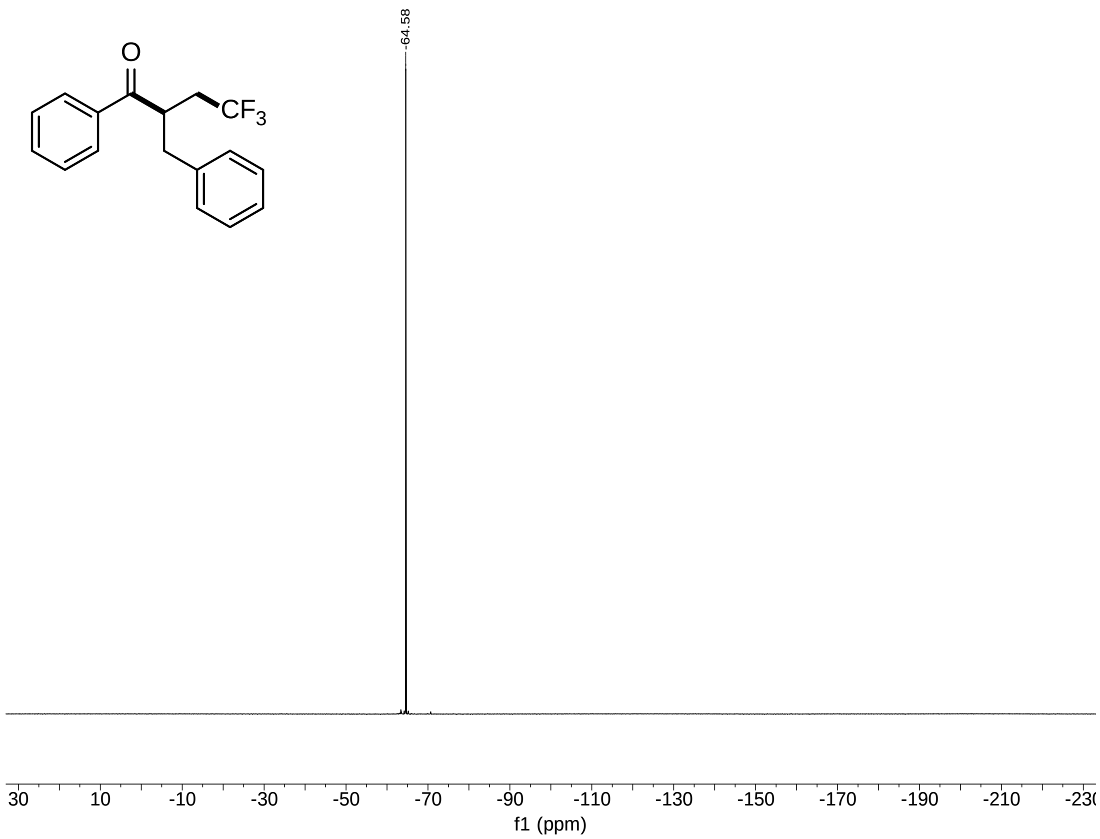

1-phenyl-2-(2,2,2-trifluoroethyl)octan-1-one (3na)

^{1}H NMR (500 MHz, $CDCl_3$)

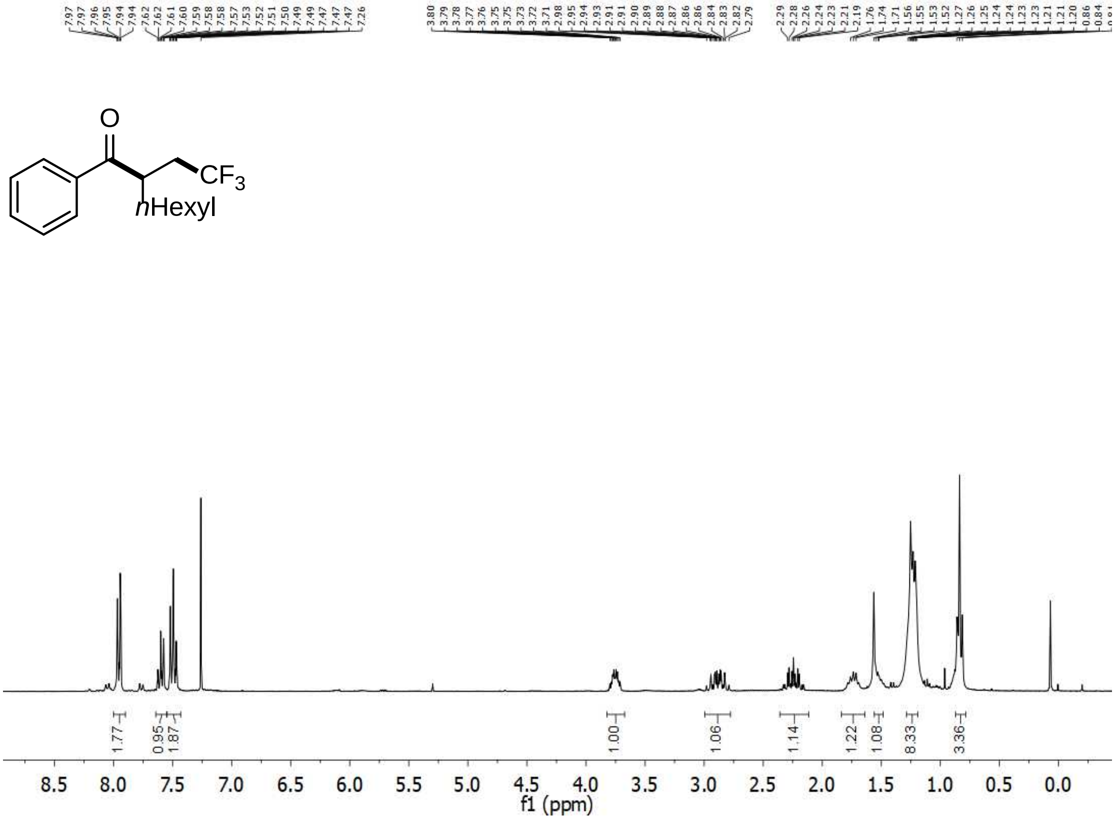

^{13}C {^{19}F} NMR (125 MHz, $CDCl_3$)

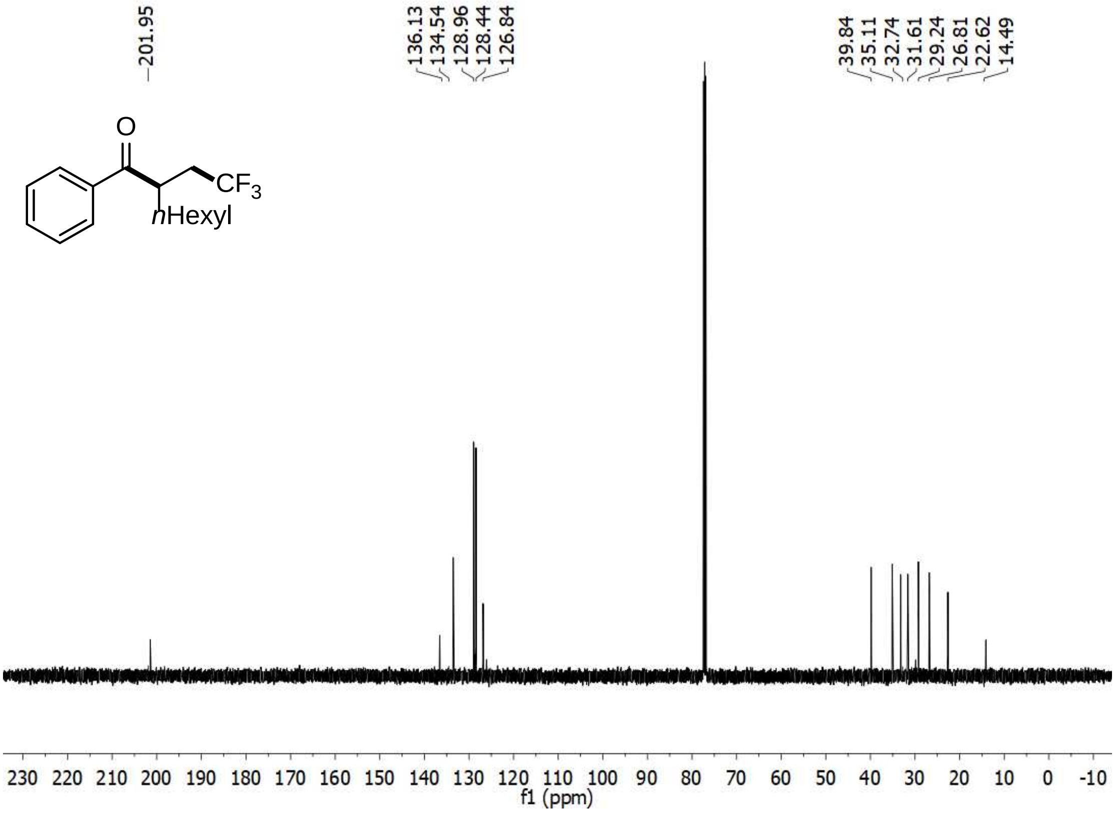

$^{19}F\{^{1}H\}$ NMR (470 MHz, $CDCl_3$)

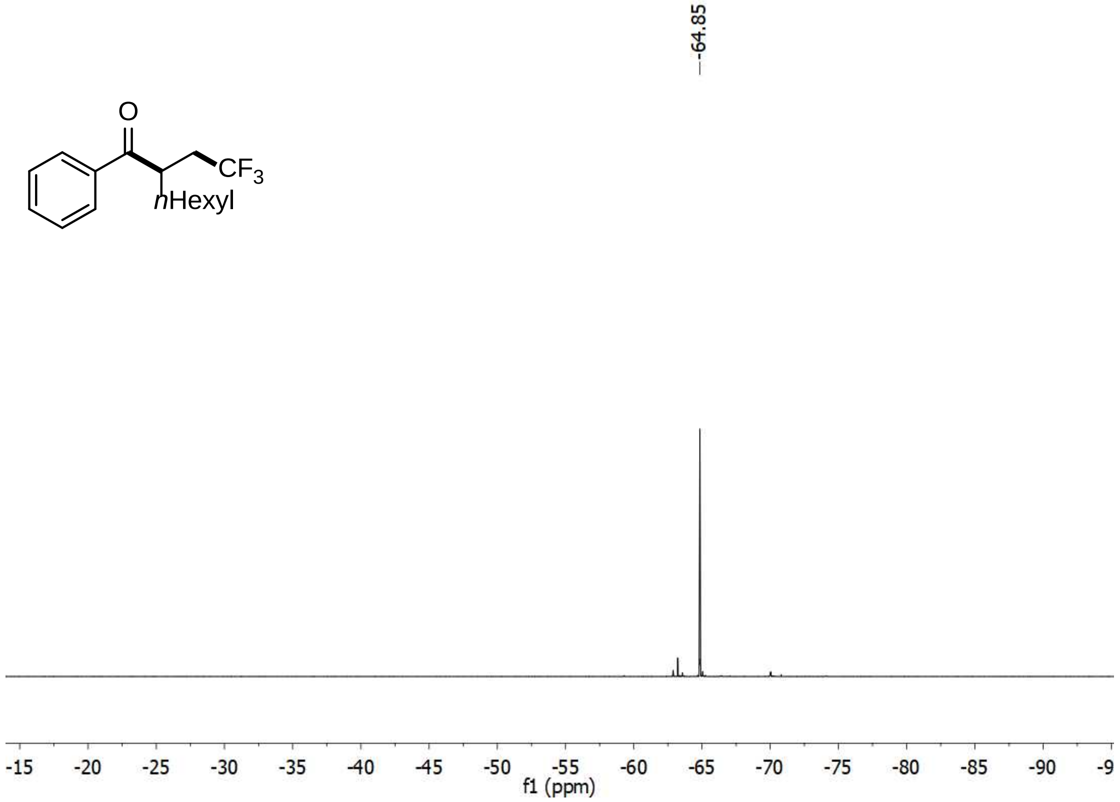

***trans*-phenyl(2-(trifluoromethyl)-2,3-dihydro-1H-inden-1-yl)methanone (*trans*-3oa)**

^{1}H NMR (300 MHz, $CDCl_3$)

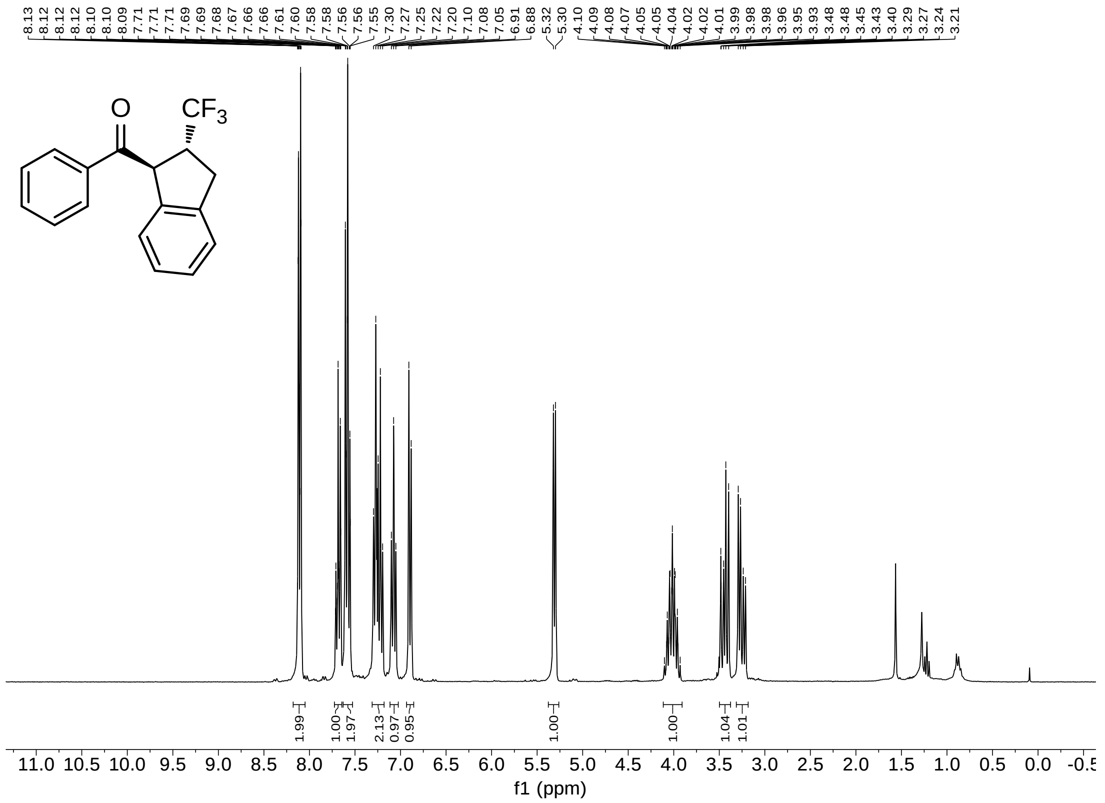

^{13}C NMR (75 MHz, $CDCl_3$)

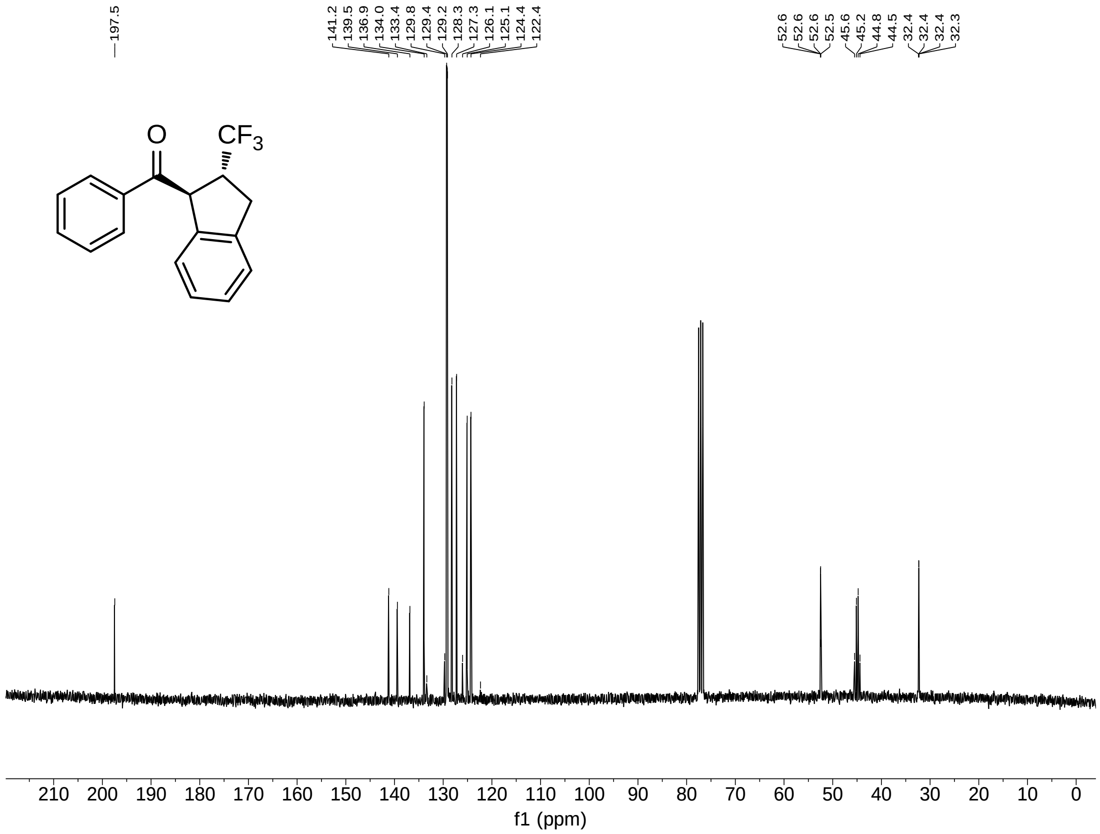

^{19}F {^{1}H} NMR (282 MHz, $CDCl_3$)

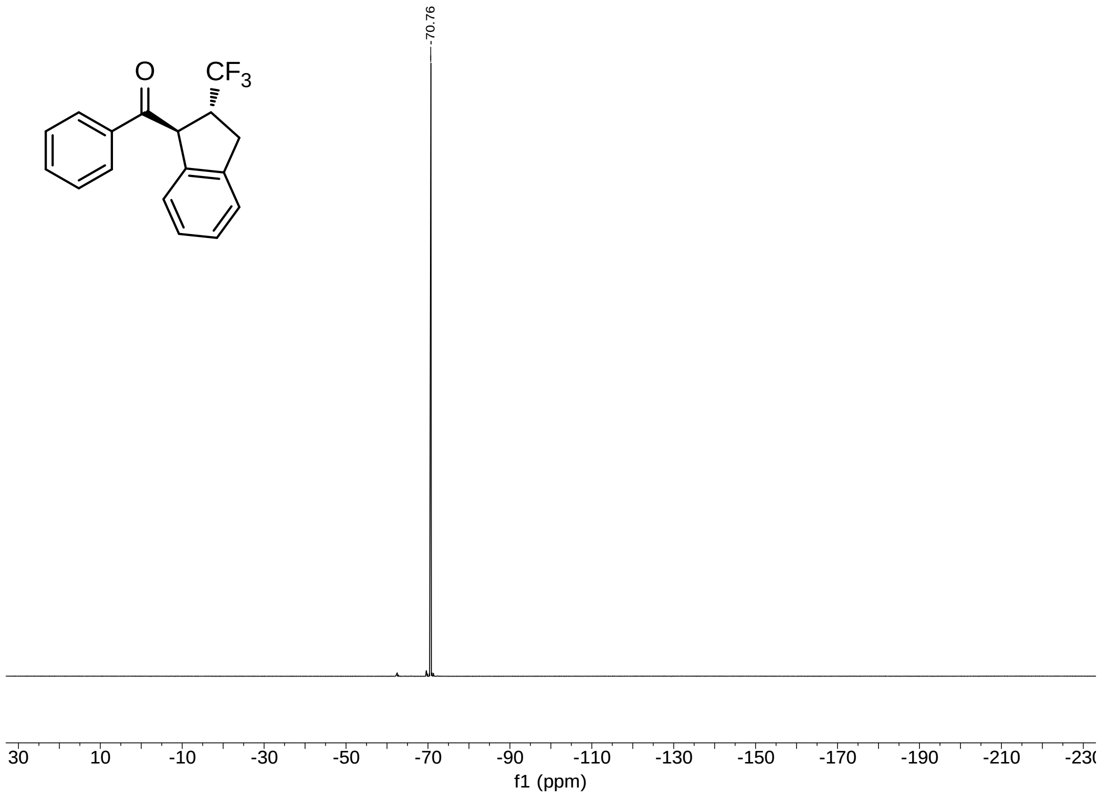

***trans*-phenyl(2-(trifluoromethyl)-2,3-dihydrobenzofuran-3-yl)methanone (*trans*-3pa)**

^{1}H NMR (600 MHz, $CDCl_3$)

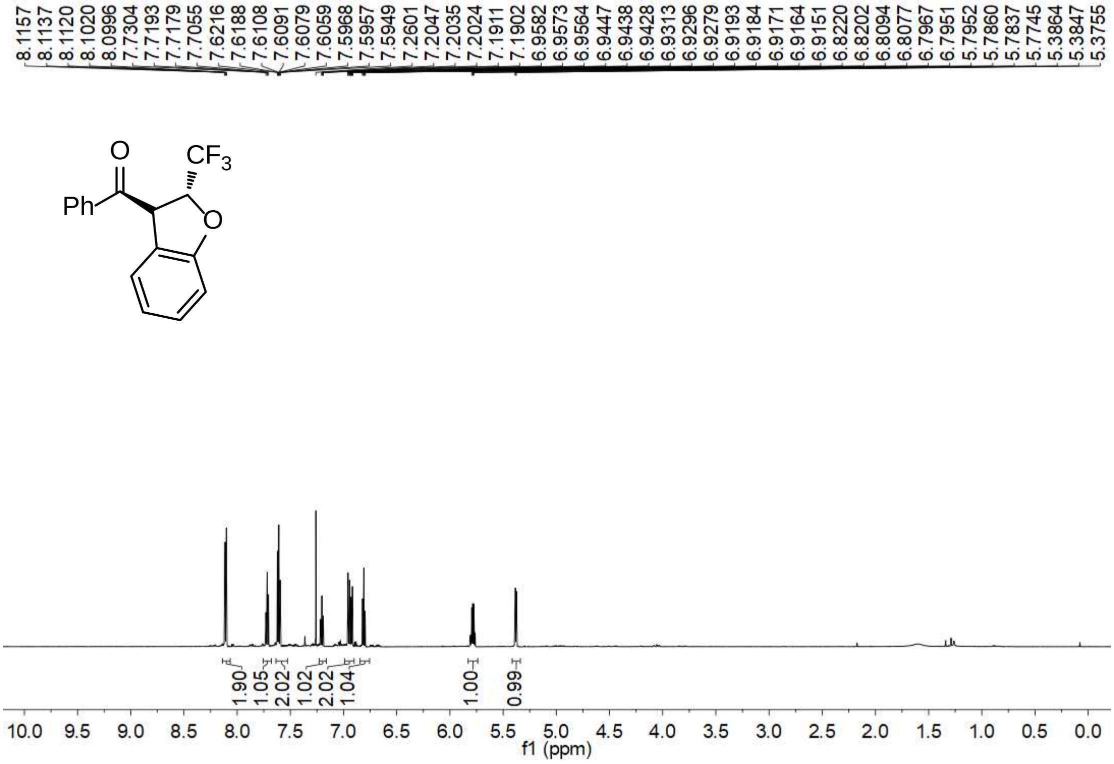

^{13}C NMR (150 MHz, $CDCl_3$)

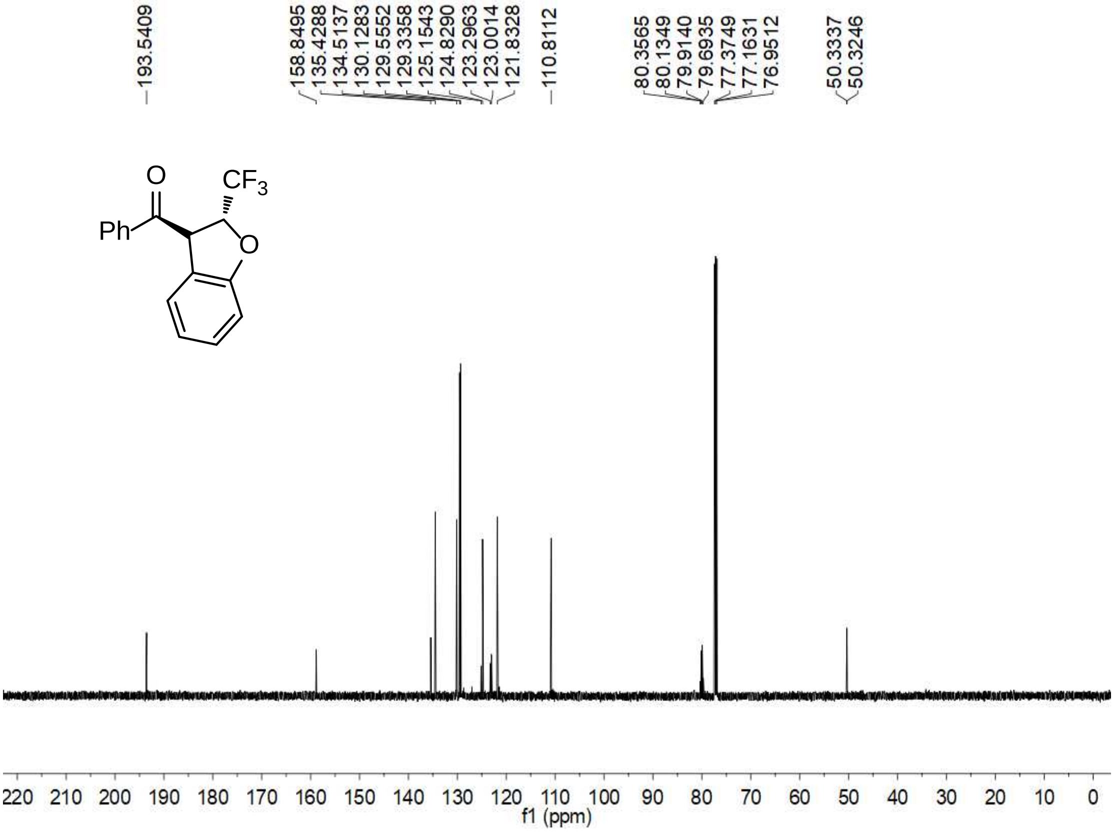

^{13}C NMR {^{19}F} (150 MHz, $CDCl_3$)

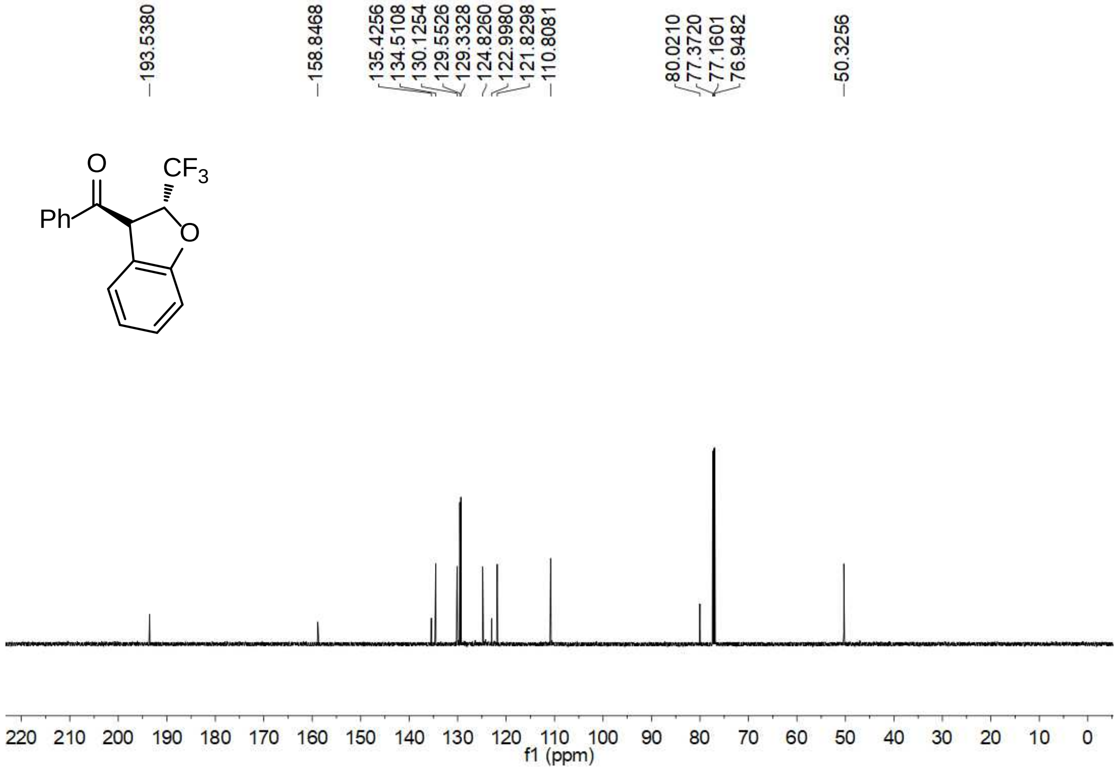

^{19}F {^{1}H} NMR (282 MHz, $CDCl_3$)

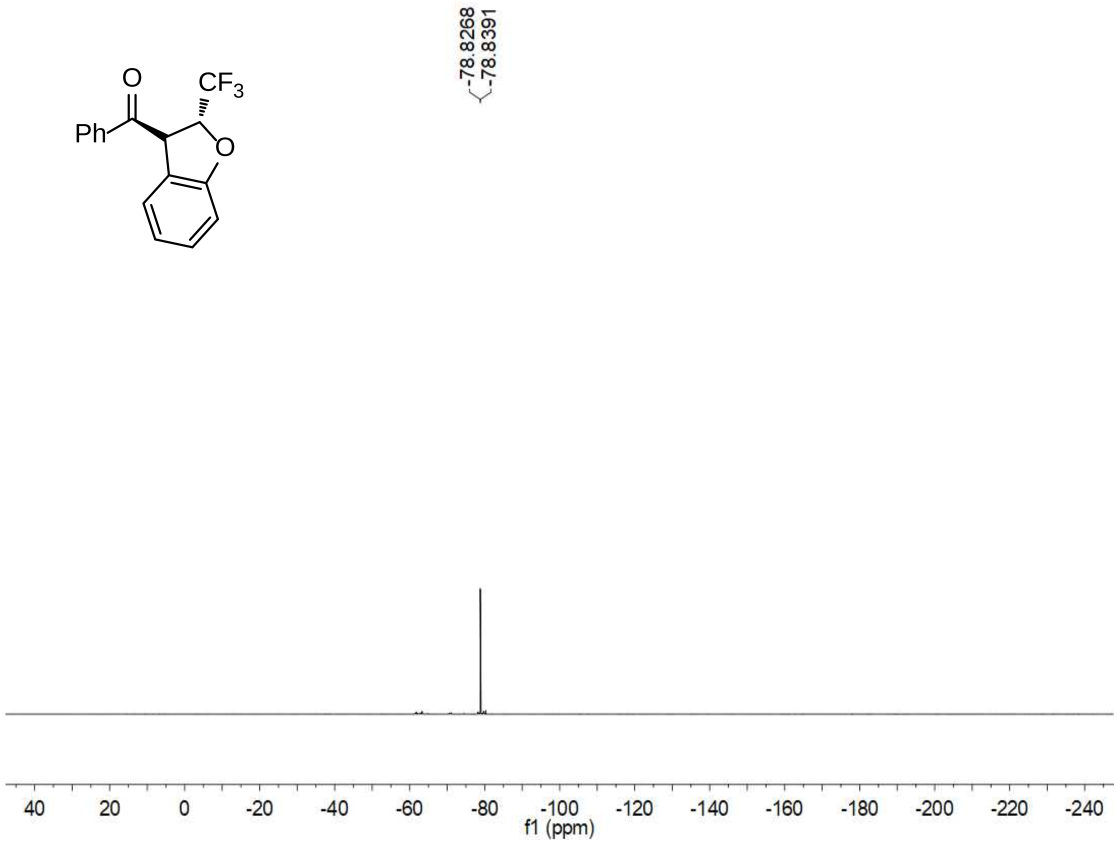

7,7,7-trifluoro-1,2-diphenylhept-4-en-1-one (*E*,*Z*-isomer, 7)

^{1}H NMR (*E*,*Z*-isomer, 300 MHz, $CDCl_3$)

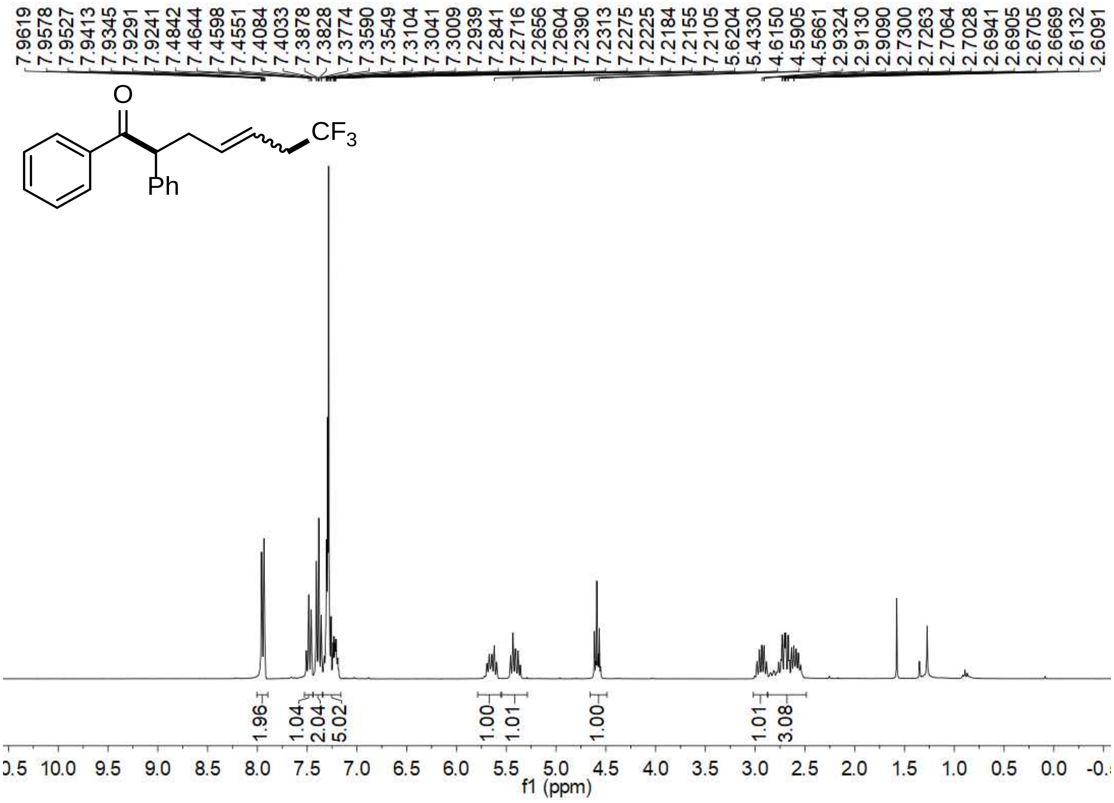

^{13}C NMR (*E*,*Z*-isomer, 75 MHz, $CDCl_3$)

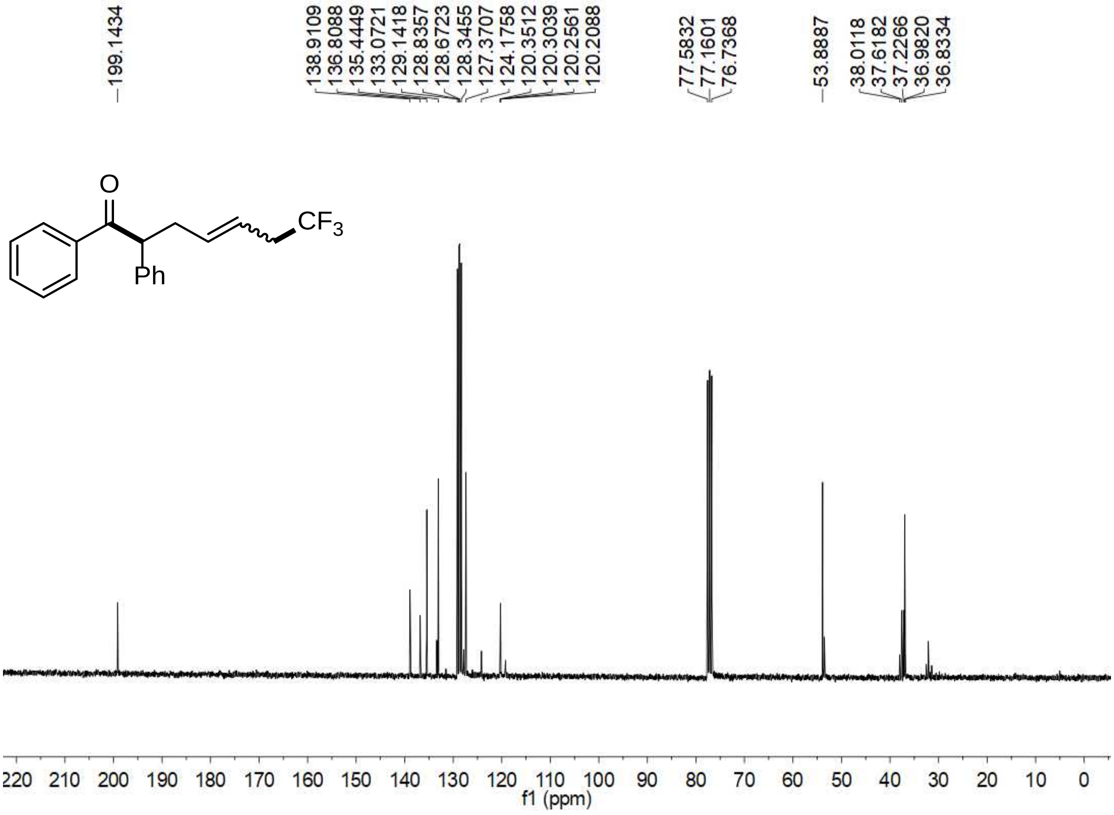

$^{19}F\ \{^1H\}$ NMR (*E*,*Z*-isomer, 282 MHz, $CDCl_3$)

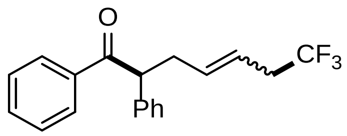

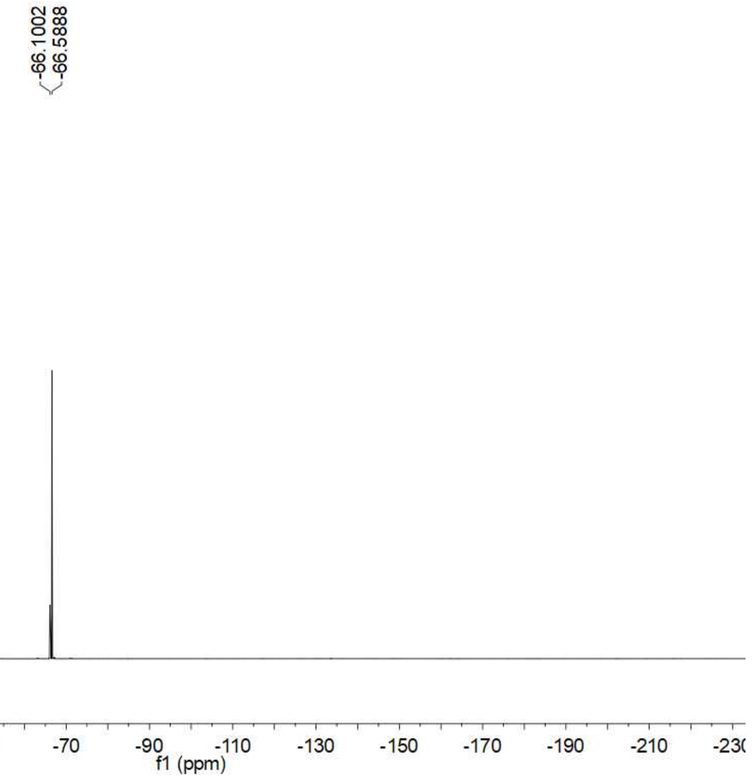

7. References

[1]. a) M. S. Kerr, J. Read de Alaniz, T. Rovis, *J. Org. Chem.* **2005**, *70*, 5725-5728; b) K. B. Ling, A. D. Smith, *Chem. Commun.* **2011**, *47*, 373-375.

[2]. G. Steiner, A. Krajete, H. Kopacka, K.-H. Ongania, K. Wurst, P. Preishuber-Pflügl, B. Bildstein, *Eur. J. Inorg. Chem.* **2004**, *2004*, 2827-2836.

[3]. A. Mavroskoufis, K. Rajes, P. Golz, A. Agrawal, V. Ruß, J. P. Götze, M. N. Hopkinson, *Angew. Chem. Int. Ed.* **2020**, *59*, 3190-3194.

[4]. S. Karthik, K. Muthuvel, T. Gandhi, *J. Org. Chem.* **2019**, *84*, 738-751.

[5]. E. Bergamaschi, F. Beltran, C. J. Teskey, *Chem. Eur. J.* **2020**, *26*, 5180-5184.

[6]. Z. Wang, X. Wang, Y. Nishihara, *Chem. Commun.* **2018**, *54*, 13969-13972.

[7]. J. A. Birrell, J.-N. Desrosiers, E. N. Jacobsen, *J. Am. Chem. Soc.* **2011**, *133*, 13872-13875.

[8]. T. Scattolin, K. Deckers, F. Schoenebeck, *Org. Lett.* **2017**, *19*, 5740-5743.

[9]. S. B. Munoz, H. Dang, X. Ispizua-Rodriguez, T. Mathew, G. K. S. Prakash, *Org. Lett.* **2019**, *21*, 1659-1663.

[10]. a) F. Gohier, A.-S. Castanet, J. Mortier, *Org. Lett.* **2003**, *5*, 1919-1922; b) S.-L. Cai, Y. Li, C. Yang, J. Sheng, X.-S. Wang, *ACS Catal.* **2019**, *9*, 10299-10304.

[11]. X. Wang, Z. Wang, Y. Asanuma, Y. Nishihara, *Org. Lett.* **2019**, *21*, 3640-3643.

[12]. J.-L. Li, Y.-Q. Liu, W.-L. Zou, R. Zeng, X. Zhang, Y. Liu, B. Han, Y. He, H.-J. Leng, Q.-Z. Li, *Angew. Chem. Int. Ed.* **2020**, *59*, 1863-1870; *Angew. Chem.* **2020**, *132*, 1879-1886.

[13]. H.-B. Yang, Z.-H. Wang, J.-M. Li, C. Wu *Chem. Commun*. **2020**, *56*, 3801-3804.
